# Endo-lysosomal proteins and ubiquitin CSF concentrations in Alzheimer’s and Parkinson’s disease

**DOI:** 10.1186/s13195-019-0533-9

**Published:** 2019-09-14

**Authors:** Simon Sjödin, Gunnar Brinkmalm, Annika Öhrfelt, Lucilla Parnetti, Silvia Paciotti, Oskar Hansson, John Hardy, Kaj Blennow, Henrik Zetterberg, Ann Brinkmalm

**Affiliations:** 1 Department of Psychiatry and Neurochemistry, Institute of Neuroscience and Physiology, the Sahlgrenska Academy at the University of Gothenburg, House V3, SU/Mölndal, SE-43180 Mölndal, Sweden; 2 Clinical Neurochemistry Laboratory, Sahlgrenska University Hospital, Mölndal, Sweden; 3 Laboratory of Clinical Neurochemistry, Neurology Clinic, University of Perugia, Perugia, Italy; 4 Department of Experimental Medicine, University of Perugia, Perugia, Italy; 5 Laboratory of Clinical Neurochemistry, Department of Medicine, University of Perugia, Perugia, Italy; 6 Clinical Memory Research Unit, Department of Clinical Sciences Malmö, Lund University, Lund, Sweden; 7 Memory Clinic, Skåne University Hospital, Malmö, Sweden; 8 Department of Molecular Neuroscience, University College London Institute of Neurology, Queen Square, London, UK; 9 UK Dementia Research Institute at UCL, London, UK

**Keywords:** Alzheimer’s disease, Biomarker, CSF, Mass spectrometry, Parkinson’s disease

## Abstract

**Background:**

Increasing evidence implicates dysfunctional proteostasis and the involvement of the autophagic and endo-lysosomal system and the ubiquitin-proteasome system in neurodegenerative diseases. In Alzheimer’s disease (AD), there is an accumulation of autophagic vacuoles within the neurons. In Parkinson’s disease (PD), susceptibility has been linked to genes encoding proteins involved in autophagy and lysosomal function, as well as mutations causing lysosomal disorders. Furthermore, both diseases are characterized by the accumulation of protein aggregates.

**Methods:**

Proteins associated with endocytosis, lysosomal function, and the ubiquitin-proteasome system were identified in the cerebrospinal fluid (CSF) and targeted by combining solid-phase extraction and parallel reaction monitoring mass spectrometry. In total, 50 peptides from 18 proteins were quantified in three cross-sectional cohorts including AD (*N* = 61), PD (*N* = 21), prodromal AD (*N* = 10), stable mild cognitive impairment (*N* = 15), and controls (*N* = 68).

**Results:**

A pilot study, including subjects selected based on their AD CSF core biomarker concentrations, showed increased concentrations of several targeted proteins in subjects with core biomarker levels indicating AD pathology compared to controls. Next, in a clinically characterized cohort, lower concentrations in CSF of proteins in PD were found compared to subjects with prodromal AD. Further investigation in an additional clinical study again revealed lower concentrations in CSF of proteins in PD compared to controls and AD.

**Conclusion:**

In summary, significantly different peptide CSF concentrations were identified from proteins AP2B1, C9, CTSB, CTSF, GM2A, LAMP1, LAMP2, TCN2, and ubiquitin. Proteins found to have altered concentrations in more than one study were AP2B1, CTSB, CTSF, GM2A, LAMP2, and ubiquitin. Interestingly, given the genetic implication of lysosomal function in PD, we did identify the CSF concentrations of CTSB, CTSF, GM2A, and LAMP2 to be altered. However, we also found differences in proteins associated with endocytosis (AP2B1) and the ubiquitin-proteasome system (ubiquitin). No difference in any peptide CSF concentration was found in clinically characterized subjects with AD compared to controls. In conclusion, CSF analyses of subjects with PD suggest a general lysosomal dysfunction, which resonates well with recent genetic findings, while such changes are minor or absent in AD.

**Electronic supplementary material:**

The online version of this article (10.1186/s13195-019-0533-9) contains supplementary material, which is available to authorized users.

## Introduction

**Fig. 1:**
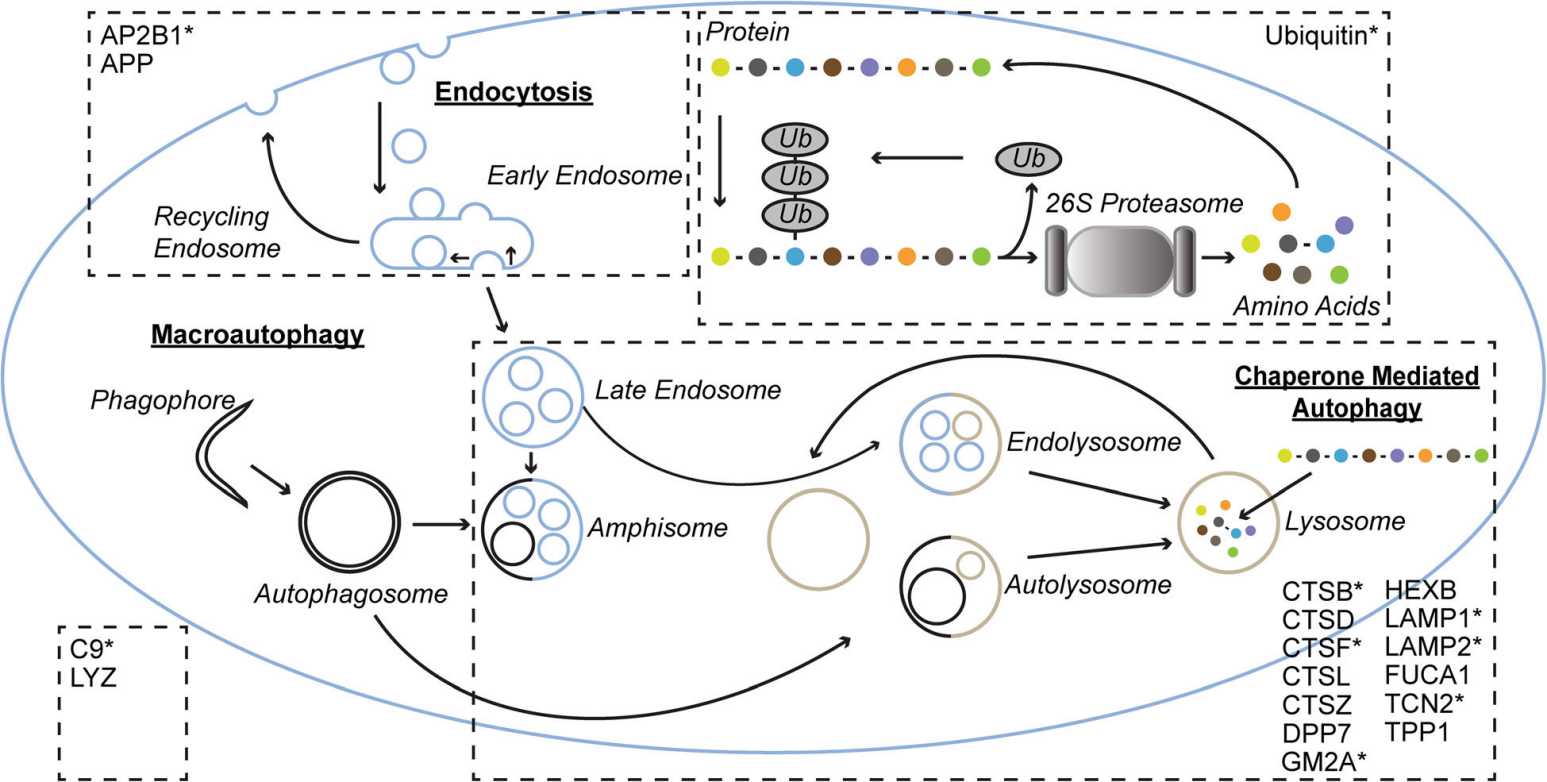
Cellular systems maintaining proteostasis. Overview of the autophagic and endo-lysosomal system and the ubiquitin-proteasome system. The proteins investigated in the present paper are shown in association with the parts and regions to which they are functionally associated or localized. Proteins with peptides found to be significantly altered in any of the three cohorts presented herein have been indicated with a star

Alzheimer’s disease (AD) [1] and Parkinson’s disease (PD) [2] are both neurodegenerative diseases characterized by the accumulation of protein aggregates. In AD, the aggregates are plaques and tangles containing aggregated amyloid β (Aβ) peptide [3] and hyperphosphorylated tau protein (P-tau) [4–6], respectively. In PD, Lewy bodies are composed of an aggregated form of α-synuclein protein [7]. Increasing evidence suggests dysfunctional proteostasis to play a central role in neurodegenerative diseases [8, 9]. Cellular proteostasis is maintained by the degradation of proteins and organelles by the autophagic and endo-lysosomal system [10, 11] and the ubiquitin-proteasome system [12] (see Fig. 1).

In AD, early pathological changes [13] have been identified by the observations of intraneuronal enlarged early endosomes [14] and an accumulation of pre-lysosomal autophagic vesicles [15]. Similarly, in PD, an accumulation of autophagic vacuoles has been seen in patients [16] and in models of disease [17], accompanied by a decrease in key player proteins in endo-lysosomal function, such as LAMP2 [18, 19] and CTSD [20]. Several lines of genetic evidence indicate the involvement of the autophagic and endo-lysosomal system in PD [2]. There is a conferred increased risk of developing PD by having mutations in the *GBA* gene [21, 22]. Loss of β-glucocerebrosidase, encoded by the *GBA* gene, cause Gaucher’s disease, a lysosomal storage disorder [23]. Recently, genetic variants in numerous lysosomal storage disorder genes were associated with PD susceptibility [24]. Similarly, genetic variants in numerous genes associated with the autophagic and endo-lysosomal system, in an aggregated manner, have been indicated to be associated with AD [25]. Indeed, genetic variants in the endocytic genes *BIN1* and *PICALM* have been associated with the susceptibility of AD [26].

In AD, the core cerebrospinal fluid (CSF) biomarkers Aβ (specifically the 42 amino acid long variant (Aβ_1–42_)), total tau protein (T-tau), and P-tau [27] have been shown to be potent in discriminating subjects with AD from controls and identify subjects with prodromal AD [28]. Some studies have also shown altered CSF concentrations in neurodegenerative diseases of proteins involved in the autophagic and endo-lysosomal system [29–31] and the ubiquitin-proteasome system [30, 32–35].

To date, there is no validated biomarker in PD. CSF α-synuclein concentrations have shown conflicting results, but often a slight decrease in PD compared to controls [36], while CSF ubiquitin concentration has been found to be unaltered [30, 34]. However, lower activity of β-glucocerebrosidase in CSF has been identified [37–39], which is further decreased in *GBA* gene mutation carriers [39]. Also, lysosomal membrane proteins have been shown to exist at lower concentrations in CSF [40]. Collectively, these findings might indicate dysfunctional lysosomes. A combination of CSF biomarkers, including the AD CSF core biomarkers (to exclude amyloid and tau pathology) and α-synuclein [41, 42], will likely have the highest diagnostic value in PD. However, longitudinal studies are lacking exploring biomarkers showing early alterations which are necessary for developing effective treatment strategies.

The aim of the current study was to explore potential CSF biomarkers associated with the autophagic and endo-lysosomal system and the ubiquitin-proteasome system in AD and PD. Identifying biomarkers reflecting dysfunctional proteostasis in AD [9] and PD [8] could add valuable information for future developments of diagnostics and treatments. Herein, an initial explorative phase was employed to identify and select the proteins of interest for quantification using solid-phase extraction (SPE) in combination with parallel reaction monitoring mass spectrometry (PRM-MS). For the purpose, we adapted a previously described method [43] holding the benefits ascribed to PRM-MS; multiplexing capabilities in complex samples [44, 45]. Using the method, one pilot cohort and two clinically characterized cohorts were investigated including subjects with AD (*N* = 62), PD (*N* = 21), prodromal AD (*N* = 10), stable mild cognitive impairment (MCI; *N* = 15), and controls (*N* = 69).

## Materials and methods

### Participants

Table 1 shows the demographics of the participants included in the cross-sectional studies of the present investigation. Participants designated as subjects having AD dementia and prodromal AD fulfilled the International Working Group biomarker criteria for research purposes [46] with a concentration of Aβ_1–42_ < 550 ng/L in combination with T-tau > 400 ng/L or P-tau phosphorylated at Thr181 (P-tau_181_) > 80 ng/L. One abnormal core biomarker was allowed for control and stable MCI subjects. No biomarker criteria were applied to subjects with PD. The pilot study consisted of subjects with concentrations of the AD CSF core biomarkers suggesting AD pathology (*N* = 19) or having normal core biomarker concentrations (controls; *N* = 24). The biomarker profile samples were collected after being analyzed in clinical routine at the Clinical Neurochemistry Laboratory, Mölndal, Sweden. In clinical study I, participants have been consecutively enrolled at the Center of Memory Disturbances of the University of Perugia. The cohort included patients affected by AD dementia (*N* = 6) diagnosed according to the National Institute on Aging-Alzheimer’s Association criteria [47, 48], patients diagnosed as PD (*N* = 10) according to the National Institute of Neurological Disorders and Stroke (NINDS) diagnostic criteria [49], and subjects diagnosed as MCI (*N* = 25) according to Petersen’s criteria [50]. Ten out of 25 developed AD (prodromal AD), while 15 remained stable for a median of 2 years follow-up (stable MCI). clinical study II consisted of a set of participants from the Swedish BioFINDER study [51] recruited at Skåne University Hospital, Sweden. The study included participants with AD dementia (*N* = 36), according to the National Institute of Neurological and Communicative Disorders and Stroke and the Alzheimer’s Disease and Related Disorders Association [52], and participants diagnosed with PD (*N* = 11), according to the NINDS diagnostic criteria [49]. Cognitively healthy volunteers who underwent cognitive testing and neurologic examination with no objective cognitive or parkinsonian symptoms were included in the control group (*N* = 44). In clinical studies I and II, clinical assessment included cognitive testing, psychiatric and neurological assessments in addition to brain imaging, and collection of CSF and blood. CSF sampling was performed after informed written consent by the patient or the caregiver.

**Table 1:** Cohort demographics

Cohort	Group	*N* (F/M)	Age, median (IQR)	*N*, *APOE* ε4 +/−^a^	Aβ_1–42_ ng/L, median (IQR)	P-tau_181_ ng/L, median (IQR)	T-tau ng/L, median (IQR)
Pilot study	Control	24 (8/16)	72 (62–74)	–	833 (714–998)	42 (36–51)	288 (218–320)
	AD	19 (9/10)	76 (71–81)^b^	–	390 (351–456)^c^	92 (81–104)^c^	940 (710–1090)^c^
Clinical study I	Stable MCI	15 (6/9)	67 (59–76)	–	1200 (880–1500)	54 (40–59)	230 (190–320)
	Prodromal AD	10 (7/3)	66 (59–74)	–	380 (270–480)^d,e^	96 (77–160)^f,g^	690 (510–1100)^f,g^
	AD	6 (2/4)	70 (57–79)	–	400 (330–460)^d^	98 (81–120)^g,h^	600 (540–860)^g,h^
	PD	10 (5/5)	60 (54–68)	–	850 (580–1000)	34 (24–48)	140 (110–180)
Clinical study II	Control	44 (30/14)	75 (68–78)	10/17	890 (660–1100)	45 (38–53)	320 (270–390)
	AD	36 (23/13)	73 (67–77)	25/10	380 (300–440)^i,j^	74 (65–100)^g,i^	680 (510–870)^g,i^
	PD	11 (6/5)	69 (68–76)	–	660 (430–910)	37 (33–47)	220 (170–320)

^a^*N* carriers of one or two *APOE* ε4 alleles (+) and non-carriers (−)

^b^*P* ≤ 0.01, vs control, Wilcoxon 2-sample rank sum test

^c^*P* ≤ 0.0001, vs control, Wilcoxon 2-sample rank sum test

^d^*P* ≤ 0.001, vs stable MCI, Kruskal-Wallis test with Dunn post hoc

^e^*P* ≤ 0.05, vs PD, Kruskal-Wallis test with Dunn post hoc

^f^*P* ≤ 0.01, vs stable MCI, Kruskal-Wallis test with Dunn post hoc

^g^*P* ≤ 0.001, vs PD, Kruskal-Wallis test with Dunn post hoc

^h^*P* ≤ 0.05, vs stable MCI, Kruskal-Wallis test with Dunn post hoc

^i^*P* ≤ 0.001, vs control, Kruskal-Wallis test with Dunn post hoc

^j^*P* ≤ 0.01, vs PD, Kruskal-Wallis test with Dunn post hoc

### CSF samples

CSF collection was performed as previously described [53, 54]. CSF was collected via lumbar puncture in polypropylene tubes, centrifuged at room temperature [54] or + 4 °C [53] for 10 min at 2000×*g*, and aliquoted. Tubes were frozen at − 80 °C pending analysis. CSF Aβ_1–42_, T-tau, and P-tau_181_ concentrations were assessed by using commercially available enzyme-linked immunosorbent assays for diagnostic purpose (INNOTEST βAmyloid1–42, hTAU-Ag, and p-TAU181Ag; Fujirebio Europe, Ghent, Belgium), according to the manufacturer’s instructions.

### Sample digestion and SPE

Digestion and SPE were performed as described previously [43] with minor modifications. In short, 100 μL of CSF from subject samples or a quality control CSF pool (QC) was mixed with 25 μL of internal standard mixture (Additional file 1: Supplementary Methods). Similarly, 100 μL of QC was mixed with 25 μL of six internal standard mixture reverse calibration curve point dilutions (Additional file 1: Supplementary Methods). Reduction and alkylation were performed in three consecutive steps by (1) the addition of 25 μL 30 mM 1,4-dithiothreitol (Sigma-Aldrich Co., Saint Louis, MO, USA) in 50 mM NH_4_HCO_3_ and shaking incubation at + 60 °C for 30 min, (2) incubation for 30 min at room temperature, and (3) addition of 25 μL 70 mM iodoacetamide (Sigma-Aldrich Co.) in 50 mM NH_4_HCO_3_ and incubation shaking at room temperature in the dark for 30 min. Digestion was performed by the addition of 25 μL 0.08 μg/μL sequencing grade modified trypsin (Promega Co., Madison, WI, USA) in 50 mM NH_4_HCO_3_ and incubation for 18 h shaking at + 37 °C. Digestion was ended by the addition of 25 μL 10% trifluoroacetic acid. SPE was performed using Oasis HLB 96-well μElution Plates (2 mg sorbent and 30 μm particle size; Waters Co., Milford, MA, USA) and a vacuum manifold station. The wells of the plate were conditioned by 2 × 300 μL methanol and equilibrated with 2 × 300 μL H_2_O. The digested samples were loaded followed by a wash of 2 × 300 μL H_2_O. Finally, the samples were eluted in 2 × 100 μL methanol and dried by vacuum centrifugation. The samples were frozen and stored at − 80 °C pending analysis.

### PRM-MS

The proteins of interest for quantification by PRM-MS are listed in Table 2. These proteins were identified as described in Additional file 1: Supplementary Methods. Digested samples were dissolved in 50 μL 50 mM NH_4_HCO_3_ shaking at room temperature for 1 h. Dissolved samples were injected (20 μL in half of the pilot study samples and in clinical study I, and 10 μL in the other half of the pilot study and in clinical study II) and separated using a Dionex UltiMate 3000 standard-LC system (Thermo Fisher Scientific Inc., Waltham, MA, USA) and a Hypersil GOLD HPLC C18 column (length 200 mm; inner diameter 2.1 mm; particle size 1.9 μm; Thermo Fisher Scientific Inc.). Mobile phases were as follow: A: 0.1% formic acid in H_2_O (*v*/*v*) and B: 0.1% formic acid and 84% acetonitrile in H_2_O (*v*/*v*). Separation was performed at a flow rate of 100 μL/min at + 40 °C with a gradient going from 2 to 6% B over 2 min, 6 to 20% B over 4 min, and finally 20 to 40% over 42 min. Micro-flow high-performance liquid chromatography was performed by online coupling to a hybrid Q Exactive mass spectrometer (Thermo Fisher Scientific Inc.). Electrospray ionization was performed in positive ion mode with a HESI-II ionization probe (Thermo Fisher Scientific Inc.) with the following settings: a spray voltage of + 4.1 kV, a heater temperature of + 300 °C, a capillary transfer tube temperature of + 320 °C, a sheath gas flow rate of 25, and an auxiliary gas flow rate of 10. Acquisition of single microscans was performed in PRM mode with an isolation window of *m/z* 3, a resolution setting of 70 k, an AGC target of 1 × 10^6^, a maximum injection time of 300 ms, and fragmentation with beam-type collision-induced dissociation (or “higher-energy collisional dissociation” (HCD) [71]). Targeting of peptides was made within a 2-min acquisition window where the retention time of the peptides was monitored in each experimental session using a mixture of stable isotope-labeled peptides. The peptides targeted and their mono-isotopic *m/z*, isolation *m/z*, and an example of retention time isolation windows are shown in Additional file 2: Table S1. The normalized collision energy, used for fragmentation with HCD, was optimized for each peptide by direct infusion of stable isotope-labeled peptide in solution and varying the energy while monitoring the intensity of the product ion in relation to the precursor ion (Additional file 2: Table S1).

**Table 2:** Protein targets

Protein name	UniProtKB accession	Gene	Function
AP-2 complex subunit beta	P63010	AP2B1	Subunit of the AP2 complex which is central in the formation of clathrin-coated vesicles in clathrin-mediated endocytosis [55].
Amyloid beta A4 protein	P05067	APP	Transmembrane protein with a number of suggested functions [56]. Processed by secretases into fragments, for example Aβ peptides [57].
Complement component C9	P02748	C9	Innate immunity component of the membrane attack complex [58].
Cathepsin B	P07858	CTSB	Lysosomal cysteine protease with carboxydipeptidase activity [59].
Cathepsin D	P07339	CTSD	Lysosomal aspartic protease [60].
Cathepsin F	Q9UBX1	CTSF	Lysosomal cysteine protease [59].
Cathepsin L1	P07711	CTSL	Lysosomal cysteine protease [59].
Cathepsin Z	Q9UBR2	CTSZ	Lysosomal cysteine protease with carboxymonopeptidase activity [59].
Dipeptidyl peptidase 2	Q9UHL4	DPP7	Lysosomal protease producing dipeptides from tripeptides [61].
Ganglioside GM2 activator	P17900	GM2A	Extracts lipids from membranes. Required for ganglioside GM2 degradation [62].
Beta-hexosaminidase subunit beta	P07686	HEXB	Lysosomal β-hexosaminidase A is a heterodimeric complex composed of Beta-hexosaminidase subunit beta and subunit alpha [63]. β-hexosaminidase A hydrolyses ganglioside GM2 with the aid of ganglioside GM2 activator [62].
Lysosome-associated membrane glycoprotein 1	P11279	LAMP1	Lysosomal transmembrane protein important in vesicle fusion [64], preserving lysosomal integrity and potentially lysosomal exocytosis [65].
Lysosome-associated membrane glycoprotein 2	P13473	LAMP2	Lysosomal transmembrane protein important in vesicle fusion [64], chaperone-mediated autophagy and preserving lysosomal integrity [65].
Lysozyme C	P61626	LYZ	Glycan cleaving polypeptide. Produced and found in secretory vesicles of neutrophils, monocytes, macrophages and epithelial cells [66].
Tissue alpha-L-fucosidase	P04066	FUCA1	Lysosomal fucosidase hydrolyzing fucose containing glycoconjugates [67].
Transcobalamin-2	P20062	TCN2	Delivers cobalamin to lysosomes where it is released [68].
Tripeptidyl-peptidase 1	O14773	TPP1	Lysosomal aspartic or serine protease producing tripeptides [69].
Ubiquitin	P0CG48	Ubiquitin^a^	Labels proteins for degradation by the proteasome [12]. Has a multitude of functions as a post-translational modification [70].

^a^Ubiquitin originate from several genes and is therefore simply referred to as ubiquitin

### Methodological evaluation

In five separate experiments, eight technical replicate QC samples were analyzed to determine experimental precision. Repeatability and intermediate precision were calculated according to ISO 5725-2 [72]. Additionally three separate reverse calibration curves were prepared and analyzed as duplicate technical replicates to investigate methodological linearity. The limit of quantification (LOQ) was set to the concentration range with a relative error ≤ 20% of calculated to nominal concentration, determined by least squares linear regression.

### Data analysis

Skyline v3.6 [73] was used for peak detection and peak area integration. Skyline was set to pick and integrate [M + H]^+^ y-ions from a data-independent acquisition method with a fixed isolation window of *m/z* 3. Evaluation of which product ions to include in the integration was performed by manual inspection of MS/MS spectra, inspecting individually extracted product ion chromatograms in Skyline and by an in-house software comparing product ion ratios. The evaluation enabled exclusion of product ions not reproducibly detected and product ions potentially afflicted by interferences. The ratio of the sum of the product ion areas of tryptic to isotope-labeled peptide was used for quantification. In the pilot study, the number of samples, approximately divided by half, was measured at different occasions. For the pilot study, the peptide ratio was normalized against the median peptide ratio of eight QC samples for each respective peptide at each occasion. The first occasion was normalized against a factor of 1 (median occasion 1/median occasion 1). The eight QC of the second occasion were used to normalize the ratio for each peptide against the first occasion using multiplication with a factor corresponding to median occasion 1 divided by median occasion 2. The clinical cohorts were analyzed coherently and thus no normalization was required.

### Statistical analysis

Normality was assessed by evaluating the distribution using histograms and boxplots as well as by using the Shapiro-Wilk *W* test of normality. The non-parametric Wilcoxon 2-sample rank sum test was used for comparing two groups and the Kruskal-Wallis test with Dunn’s test for multiple comparisons, comparing all pairs, for more than two groups. *P* ≤ 0.05 was considered statistically significant. Correlations were investigated using Spearman’s rank correlation where Spearman’s *ρ* ≥ 0.8 or ≤ − 0.8 and a *P* value ≤ 0.01 were considered to indicate a correlation. Statistics were calculated using JMP Pro v.13.2.1 (SAS Institute Inc., Cary, NC, USA), and graphs were created using GraphPad Prism v.8.2.0 (GraphPad Software, Inc., La Jolla, CA, USA). Calibration curves were created using least square linear regression and evaluated in GraphPad Prism (GraphPad Software, Inc.).

## Results

### Identification of target proteins and peptides

Initial exploration was performed to identify proteins of relevance to include in the panel. First three technical replicates, dissolved in 0.1% formic acid prior to injection, and then three technical replicates dissolved in 0.1% formic acid, 50 mM NH_4_HCO_3_, or 20% acetonitrile were analyzed. The proteins identified in steps 1 and 2 are shown in Additional file 3: Table S2. Proteotypic stable isotope-labeled peptides were acquired for method development. Ubiquitin in CSF was targeted in a previous study [35], and now two proteotypic ubiquitin peptides were selected for quantification. In total, 51 peptides from 19 proteins were included in the method, including added bovine serum albumin as a tool to monitor the status and variability of the method within and between experiments.

### LOQ and methodological precision

The LOQ was investigated for all peptides by creating a six-point reversed calibration curve by using least squares linear regression and was determined as the concentration range with a relative error of ≤ 20% of calculated to nominal concentration. By analyzing eight technical replicate QC samples at five separate time points, the repeatability and intermediate precision of the peptides were found to range between 3 and 22% and 7 and 38%, respectively. Three peptides, DPP7 (40–47), LAMP2 (153–161), and FUCA1 (114–130), were measured outside the LOQ; however, they displayed a precision in line with the precision of peptides measured within the LOQ. The peptide LOQs are shown in Additional file 2: Table S1 and Additional file 4: Figure S1. Information about approximate peptide concentrations in CSF and precision data are shown in Additional file 2: Table S1. Additionally, extracted ion chromatograms and MS/MS spectra for all peptides included in the method are shown in Additional file 5: Figure S2.

### CSF protein concentrations in the pilot study

**Fig. 2:**
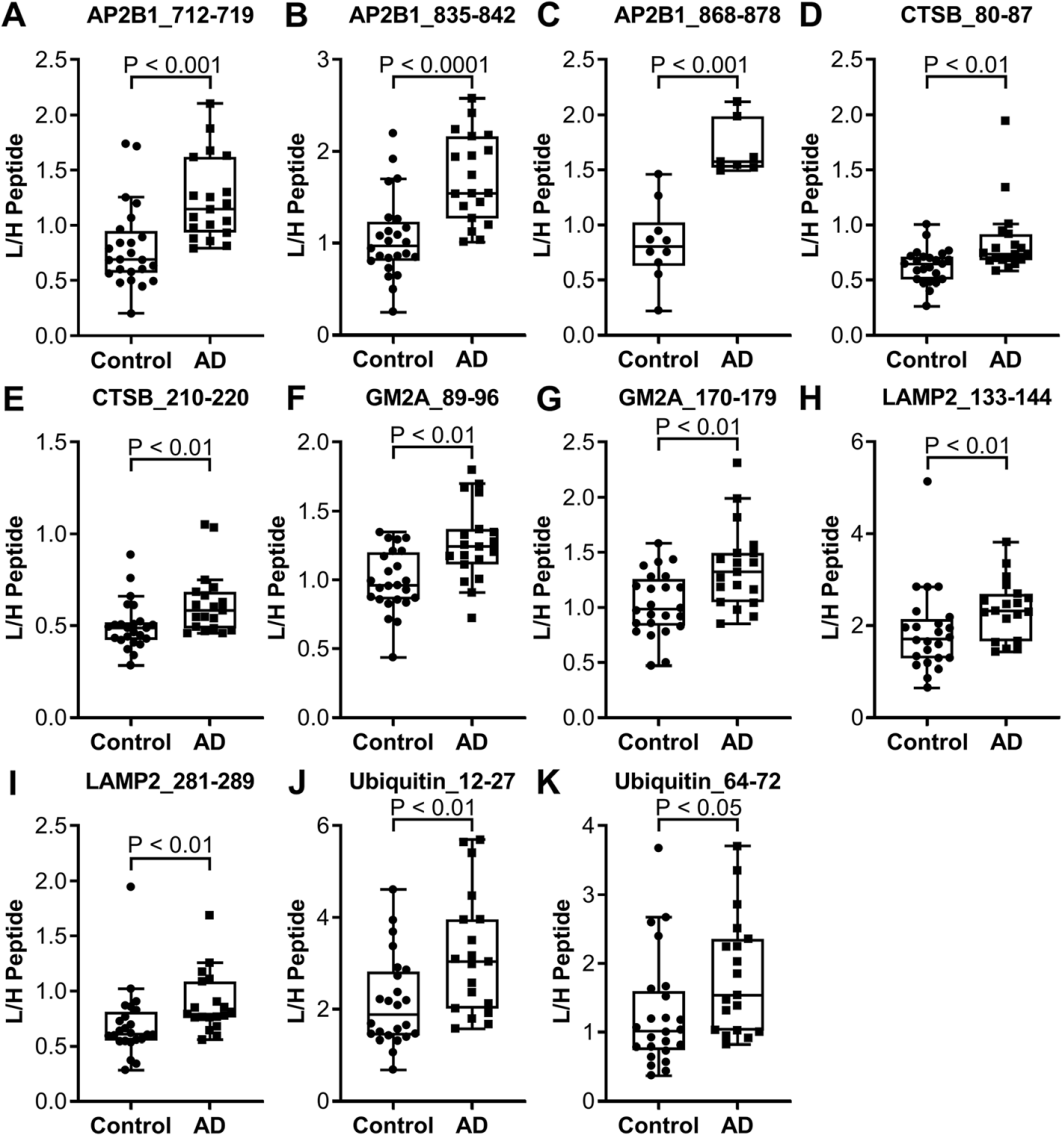
CSF endo-lysosomal proteins and ubiquitin concentrations in the pilot study. The pilot study included subjects designated as controls (*N* = 24) or AD (*N* = 19) based on their CSF AD core biomarker profile. Shown are peptides found to be significantly increased in AD compared to controls; **a** AP2B1_712–719 (*P* < 0.001), **b** AP2B1_835–842 (*P* < 0.0001), **c** AP2B1_868–878 (*P* < 0.001; peptide measured in: AD, *N* = 7, and control, *N* = 10), **d** CTSB_80–87 (*P* < 0.01), **e** CTSB_210–220 (*P* < 0.01), **f** GM2A_89–96 (*P* < 0.01), **g** GM2A_170–179 (*P* < 0.01), **h** LAMP2_133–144 (*P* < 0.01), **i** LAMP2_281–289 (*P* < 0.01), **j** Ubiquitin_12–27 (*P* < 0.01), and **k** Ubiquitin_64–72 (*P* < 0.05). Statistics were calculated using Wilcoxon 2-sample rank sum test and the graphs show Tukey boxplots

The pilot study, consisting of subjects having normal CSF concentrations of the AD CSF core biomarkers or concentrations suggesting AD pathology, was analyzed (control, *N* = 24; AD, *N* = 19). The CSF concentration of AP2B1, CTSB, GM2A, LAMP2, and ubiquitin was significantly increased in the AD group compared to that in the controls (see Fig. 2 and Table 3). All peptide measurements are presented in Additional file 6: Table S3. Furthermore, analyzing correlations in the pilot study, P-tau_181_ was found to correlate with one of the AP2B1 peptides (712–719) in the control group (see Additional file 7: Table S4). No further correlations were found between the AD CSF core biomarkers and the peptides measured, neither in the AD nor control group. Typically, but not for all peptides, there was a correlation between peptides originating from the same protein. Age was found to be significantly different between AD and controls (Table 1); however, there was no correlation between age and any of the peptides measured (Additional file 7: Table S4).

**Table 3:** Proteins and peptides showing significant differences

Cohort	Change	Protein (significant peptides^a^/peptides measured)
Pilot study	↑AD vs control	AP2B1 (3/3)^b^, CTSB (2/3), GM2A (2/2), LAMP2 (2/4), and ubiquitin (2/2)
Clinical study I	↓PD vs prodromal AD	AP2B1 (1/3)^b^, CTSF (1/4), LAMP1 (1/3), LAMP2 (3/4), and ubiquitin (2/2)
Clinical study II	↓PD vs control	AP2B1 (2/2)^b^, CTSF (2/4), and ubiquitin (1/2)
	↓PD vs AD	AP2B1 (2/2)^b^, C9 (1/5), CTSB (1/3), CTSF (4/4), GM2A (2/2), TCN2 (2/3), and ubiquitin (2/2)

^a^*P* value of ≤ 0.05 using Wilcoxon 2-sample rank sum test or Kruskal-Wallis test with Dunn post hoc

^b^Three peptides were measured in a subset of subjects in the pilot study and all subjects in clinical study I. Two peptides were measured in the rest of the pilot study subjects and clinical study II

### CSF protein concentrations in clinical cohorts

**Fig. 3:**
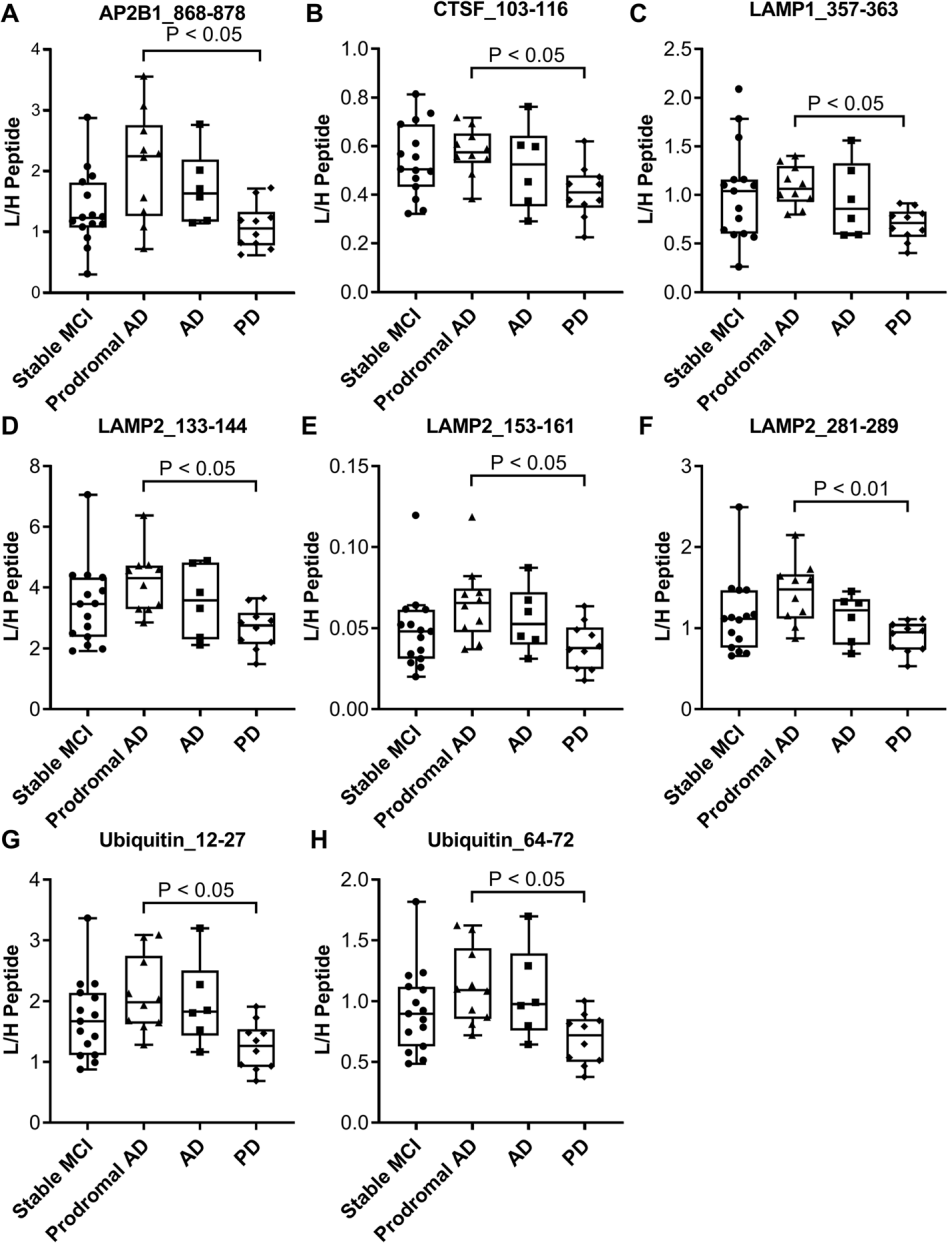
CSF endo-lysosomal proteins and ubiquitin concentrations in clinical study I. Clinical study I included cross-sectional samples from clinically characterized subjects with AD (*N* = 6) and PD (*N* = 10) as well as longitudinal samples from subjects with prodromal AD (*N* = 10) and stable MCI (*N* = 15). A significant decreased concentration of **a** AP2B1_868–878 (*P* < 0.05), **b** CTSF_103–116 (*P* < 0.05), **c** LAMP1_357–363 (*P* < 0.05), **d** LAMP2_133–144 (*P* < 0.05), **e** LAMP2_153–161 (*P* < 0.05), **f** LAMP2_281–289 (*P* < 0.01), **g** Ubiquitin_12–27 (*P* < 0.05), and **h** Ubiquitin_64–72 (*P* < 0.05) was identified in PD compared to prodromal AD. Statistics were calculated using Kruskal-Wallis test with Dunn’s test for multiple comparisons and the graphs show Tukey boxplots

In clinical study I, the investigation continued with measurements of CSF protein concentrations in CSF collected from subjects with prodromal AD (*N* = 10), stable MCI (*N* = 15), AD dementia (*N* = 6), and PD (*N* = 10). The level of several proteins was found to be significantly decreased in PD compared to prodromal AD (see Fig. 3 and Table 3). These included AP2B1, CTSF, LAMP1, LAMP2, and ubiquitin. No correlation was found between the AD CSF core biomarkers or age and the peptide concentrations in the stable MCI group (see Additional file 7: Table S4 for all correlation information). In the prodromal AD group, P-tau_181_ correlated with two AP2B1 peptides (712–719 and 835–842) and a LAMP2 peptide (153–161), and a correlation was also found between GM2A (89–96) and age. In the AD group, P-tau_181_ correlated with one APP peptide (289–301) and Aβ_1–42_ correlated with one C9 peptide (146–154). In the PD group, P-tau_181_ correlated with all AP2B1 peptides, one LAMP2 peptide (153–161), and all ubiquitin peptides. Also, in the PD group, Aβ_1–42_ correlated with a FUCA1 peptide (163–173).

**Fig. 4:**
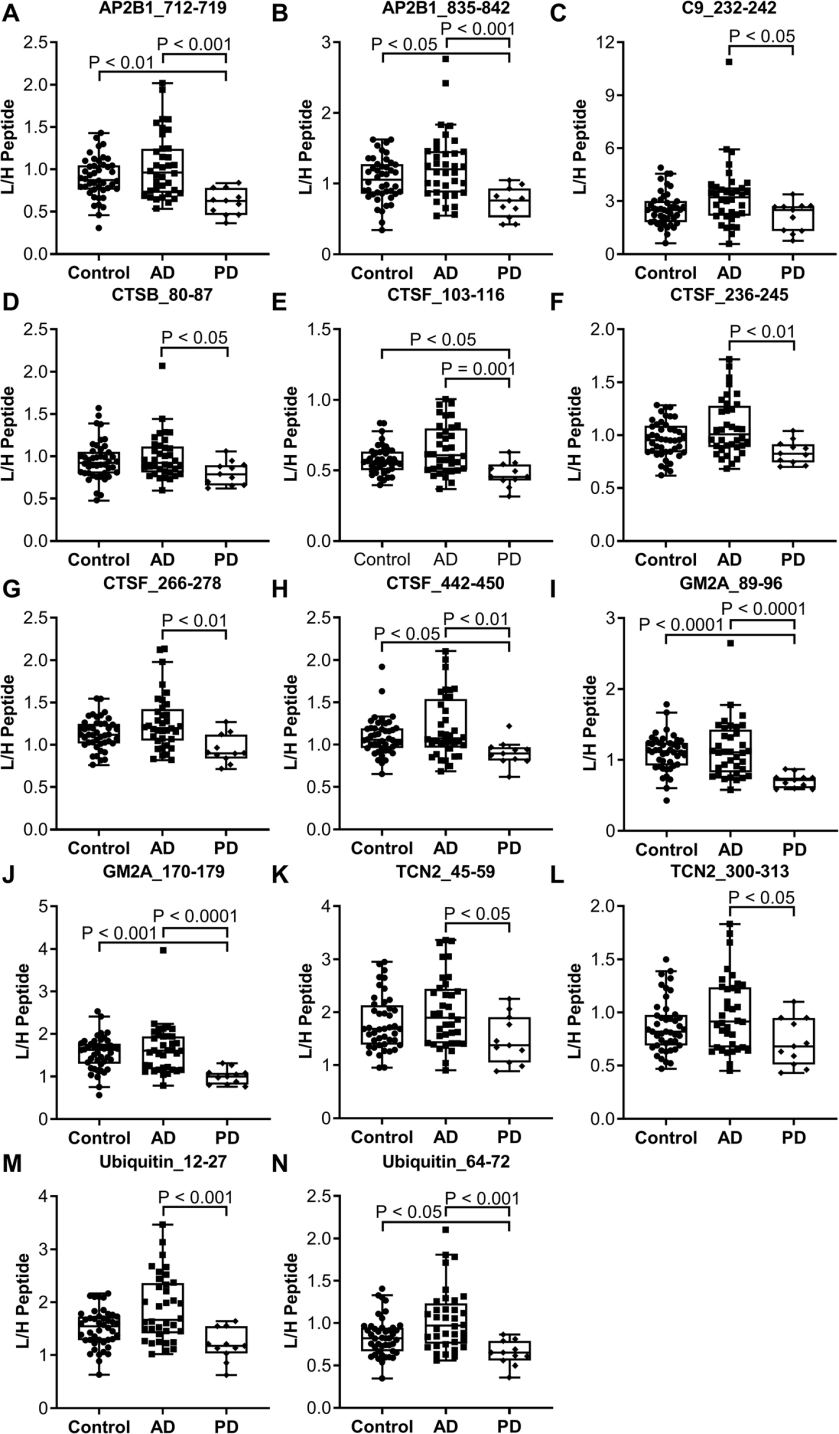
CSF endo-lysosomal proteins and ubiquitin concentrations in clinical study II. Clinical study II included cross-sectional samples from clinically characterized subjects with AD (*N* = 36) and PD (*N* = 11) as well as cognitively normal healthy controls (*N* = 44). A significant decreased concentration of **a** AP2B1_712–719 (*P* < 0.001), **b** AP2B1_835–842 (*P* < 0.001), **c** C9_232–242 (*P* < 0.05), **d** CTSB_80–87 (*P* < 0.05), **e** CTSF_103–116 (*P* = 0.001), **f** CTSF_236–245 (*P* < 0.01), **g** CTSF_266–278 (*P* < 0.01), **h** CTSF_442–450 (*P* < 0.01), **i** GM2A_89–96 (*P* < 0.0001), **j** GM2A_170–179 (*P* < 0.0001), **k** TCN2_45–59 (*P* < 0.05), **l** TCN2_300–313 (*P* < 0.05), **m** Ubiquitin_12–27 (*P* < 0.001), and **n** Ubiquitin_64–72 (*P* < 0.001) was found in PD compared to AD. Additionally, a significant decreased concentration of **a** AP2B1_712–719 (*P* < 0.01), **b** AP2B1_835–842 (*P* < 0.05), **e** CTSF_103–116 (*P* < 0.05), **h** CTSF_442–450 (*P* < 0.05), **i** GM2A_89–96 (*P* < 0.0001), **j** GM2A_170–179 (*P* < 0.001), and **n** Ubiquitin_64–72 (*P* < 0.05) was identified in PD compared to controls. Statistics were calculated using Kruskal-Wallis test with Dunn’s test for multiple comparisons and the graphs show Tukey boxplots

The finding in clinical study I that proteins had lower concentration in PD and no alterations in prodromal AD or AD dementia called for an additional investigation in a clinical cohort. Clinical study II consisted of subjects with AD dementia (*N* = 36), PD (*N* = 11), and cognitively normal healthy controls (*N* = 44). In clinical study II, the concentrations of AP2B1, CTSF, GM2A, and ubiquitin were significantly decreased in PD compared to AD and controls (Fig. 4 and Table 3). Also, C9, CTSB, CTSF, TCN2, and ubiquitin were found to be significantly decreased in PD compared to AD. All peptides with significantly different CSF concentrations in clinical studies I and II have been combined showing the results of both studies in Additional file 8: Figure S3. In clinical study II, correlations were identified between the AD CSF core biomarkers T-tau and P-tau_181_ and the AP2B1 and ubiquitin peptides in the control group (Additional file 7: Table S4). Moreover, both AP2B1 peptides, one LAMP2 peptide (281–289), and one ubiquitin peptide (12–27) correlated with P-tau_181_ in the AD group. Finally, both AP2B1 peptides, two LAMP2 peptides (133–144 and 153–161), and one ubiquitin peptide (12–27) correlated with P-tau_181_ in the PD group. A subset of subjects in clinical study II had a known *APOE* genotype. Being *APOE* ε4 positive by having one or two ε4 alleles had a significant effect on the CSF protein concentrations in the control group. The level of C9, CTSF, DPP7, and GM2A was decreased in controls being *APOE* ε4-positive carriers (*N* = 10) compared to non-carriers (*N* = 17) (see Additional file 9: Figure S4). There was no association between *APOE* ε4 and CSF protein concentrations in AD (carriers, *N* = 25; non-carriers, *N* = 10).

## Discussion

In the present study, an explorative approach has been used to investigate the CSF concentrations of proteins associated with endocytosis, lysosomal function, and the ubiquitin-proteasome system in AD and PD (see Fig. 1). A method quantifying 18 proteins by measuring 50 peptides, using 100 μL CSF, has been developed. The LOQ and precision have been described for each peptide. Initially, the pilot study, including subjects having an AD CSF core biomarker profile or a normal profile (controls), showed increased CSF concentrations of several proteins, AP2B1, CTSB, GM2A, LAMP2, and ubiquitin, in AD compared to controls. Exploration of a clinically characterized cohort, clinical study I, instead showed decreased CSF concentrations of AP2B1, CTSB, CTSF, LAMP1, LAMP2, and ubiquitin in PD compared to prodromal AD. A recent study, using a similar method as described herein, showed increased CSF concentrations of proteins involved in vesicular transport and synaptic function in prodromal AD compared to AD and controls [74]. Presently, further investigation in an additional clinically characterized cross-sectional cohort confirmed decreased CSF concentrations of several proteins in PD compared to AD and controls, including AP2B1, C9, CTSB, CTSF, GM2A, TCN2, and ubiquitin. Proteins repeatedly identified at altered CSF concentrations, in more than one of the four studies, were AP2B1, CTSB, CTSF, GM2A, LAMP2, and ubiquitin, which position them as the most potent markers of those investigated.

The importance of lysosomal function in PD has been indicated by an increased risk of developing the disease in carriers of *GBA* gene mutations [21, 22] and increased susceptibility having genetic variants in genes associated with lysosomal storage disorders [24]. β-glucocerebrosidase has been shown at decreased amounts [75] and activities [75, 76] in the *substantia nigra* of *GBA* gene mutation carriers and non-carriers. Lysosomal alterations in PD seem also to be measurable in CSF as shown by decreased activities of β-glucocerebrosidase in PD compared to controls [37–39]. However, unaltered β-glucocerebrosidase activity has also been indicated [77]. Additionally lower activities have been shown in dementia with Lewy bodies [78].

We measured primarily lysosomal proteins and did identify decreased CSF concentrations of several in PD. We found decreased concentrations of AP2B1, essential in clathrin-mediated endocytosis [55], in PD compared to prodromal AD, AD, and controls. To our knowledge, AP2B1 has not been previously investigated in CSF from subjects with AD or PD. Further, concentrations of CTSB and CTSF peptides were also decreased in PD compared to controls, prodromal AD, and AD dementia. The cathepsin peptides selected originated from the propeptide, propeptide-active chain cleavage site, and the active chains to provide functional information. However, no such information could be deduced from the present findings. No alterations of CSF concentrations of CTSD, CTSL, or CTSZ were found in PD. Previous investigations have revealed decreased [39] or unchanged [77] CTSD enzymatic activity in CSF in PD compared to controls. GM2A concentration, which in cooperation with β-hexosaminidase degrades ganglioside GM2 [62], was decreased in PD compared to AD and controls in clinical study II. Previously, the CSF concentration has been found increased in dementia with Lewy bodies but unaltered in PD compared to controls [30]. The LAMP1 and LAMP2 concentrations were decreased in PD compared to prodromal AD in clinical study I. Chu et al. [20] showed decreased levels of LAMP1 in α-synuclein containing neurons in the substantia nigra of subjects affected by PD. Furthermore, unaltered [30] or decreased [40] LAMP1 CSF concentrations have been indicated in PD compared to controls. Three isoforms of LAMP2 exists: A, B, and C [79]. The peptides measured in the present study are not isoform specific. LAMP2A is fundamental in chaperone-mediated autophagy (CMA) [80], in which it confers a rate-limiting component [81, 82]. Interestingly, CMA decreases with aging [82] and has been shown to be involved in the degradation of α-synuclein [83, 84]. Murphy et al. showed that the level of LAMP2A decreased and related to accumulating amounts of α-synuclein in early PD [19]. Also, the level of LAMP2A in substantia nigra pars compacta and amygdala has been shown to be decreased in PD [18]. The CSF concentration of LAMP2 has previously been indicated to be decreased in PD [40]. The CSF ubiquitin concentration in PD has been suggested to be unchanged [30, 34, 85]. However, in the present investigation, we identified a decreased concentration of ubiquitin in PD compared to prodromal AD, AD dementia, and controls. Previously, we found no significant difference in the CSF concentration of full-length ubiquitin in PD compared to controls or progressive supranuclear palsy [35]. Ubiquitin has been shown to be a component of Lewy bodies [86, 87]. How this would contribute to lower ubiquitin concentration in PD remains speculative. We found no differences in the CSF concentration of HEXB between groups. Previously, the CSF enzymatic activity of β-hexosaminidase has been shown to be either increased [38], unchanged [37, 77], or reduced [39] in PD compared to controls. We also identified significantly decreased concentration of TCN2 in PD compared to AD in clinical study II. TCN2 transports cobalamin to the lysosome, where it is released [68]. Disturbed lysosomal acidification and protease inhibition [88] seem to increase lysosomal cobalamin retention. Lysosomal dysfunction might thus cause retention of cobalamin as well as TCN2. C9 is a component of the innate immunity membrane attack complex [58]. We observed a significantly lower concentration of C9 (232–242) in PD compared to AD in clinical study II. C9 expression in the brain has been shown to be higher in AD compared to controls [89, 90].

In AD, there is an accumulation of intraneuronal enlarged early endosomes [14] and pre-lysosomal autophagic vesicles [15]. These alterations are accompanied by an increased expression and presence of endosomal and lysosomal proteins [91–94]. Additionally, endosomal, lysosomal, and autophagic proteins have shown elevated CSF concentrations in AD compared to controls [29]. We did not observe significant differences for any peptides in AD or prodromal AD compared to stable MCI in clinical study I or between AD dementia and controls in clinical study II. The pilot study consisted of biochemically characterized subjects suggesting AD pathology in the AD group. The subjects could have AD dementia, prodromal AD, or preclinical AD, alone or in combination with other comorbidities. Similarly, the pilot study controls are not necessarily healthy as they have sought medical advice with a neurological implication. This might in part explain the discrepancies in the results between the pilot study and the clinical studies. Nonetheless, we found elevated concentrations of AP2B1 in AD compared to non-AD controls in the pilot study. Musunuri et al. [95] have previously found decreased subunit α-1 AP2 levels in the temporal neocortex in AD compared to controls. Also, the CTSB CSF concentration was increased in AD compared to non-AD controls in the pilot study. Enzymatically active CTSB and CTSD have been identified as components of plaques [96] and shown to have increased cortical and hippocampal immunoreactivity in AD [91]. Increased CTSB plasma concentration has previously been shown in AD [97]. However, the CSF concentration in AD has been suggested to be unaltered [29, 97] whereas the proenzyme form of CTSL has exhibited increased CSF concentration in AD compared to controls [29]. However, we did not detect any significant differences for any of the CTSL peptides. Significantly increased concentrations of LAMP2 peptides were observed for AD compared to non-AD controls in the pilot study. Previous investigations have suggested increased CSF concentrations of LAMP2 [29, 31] as well as LAMP1 [29, 30] in AD. Recently, GM2A was found to have significantly increased CSF concentration in AD compared to controls [30]. We here show increased concentrations of GM2A peptides in AD compared to non-AD controls in the pilot study. The CSF concentration of ubiquitin has been shown to be increased in AD [30, 32–35], Creutzfeldt-Jakob disease [34, 98, 99], and dementia with Lewy bodies [30] compared to controls. Also, the CSF ubiquitin concentration in prodromal AD has been shown to be higher compared to subjects with stable MCI [100]. Herein, the CSF concentration of ubiquitin peptides was found increased in AD compared to non-AD controls in the pilot study. Previously, we have measured full-length ubiquitin [35]; however, the peptides measured in the current analysis could additionally originate from post-translationally modified substrates or polyubiquitin chains.

The CSF concentrations for APP, DPP7, LYZ, FUCA1, and TPP1 were not significantly altered. When recently measured, no difference in APP peptide concentration was found between AD and controls [43]. Furthermore, a recent meta-analysis showed no difference between the CSF levels of soluble α- and β-APP in AD compared to controls [28]. The activity of DPP7 in CSF and serum has been suggested to be increased in PD [101]. However, the frontal cortex DPP7 activity in PD and dementia with Lewy bodies has been indicated to be low compared to controls whereas no difference was detected in AD [102]. Concordant with our recent findings [43], no alterations in LYZ peptides was identified between AD and controls. The CSF concentration of LYZ has previously been suggested to be increased in AD compared to controls [103, 104]. To our knowledge, the CSF level of FUCA1 in AD or PD has not previously been investigated. FUCA1 is a lysosomal glycosidase [67], and mutations in *FUCA1* cause the lysosomal storage disorder fucosidosis [105].

ApoE is a lipid transporter of which three isoforms exists: E2, E3, and E4 (encoded by the ε2, ε3, and ε4 alleles, respectively, of the *APOE* gene) [106]. In a gene dose-dependent manner, the *APOE* ε4 allele is the most prominent genetic risk factor for AD susceptibility [107]. Previously, it has been shown that enlarged endosomes, as seen early on in AD, are accentuated and even larger in carriers of *APOE* ε4 [13]. Furthermore, apoE regulate Aβ production [108] which is amplified by the E4 isoform [108] and apoE4, in conjunction with Aβ, cause lysosomal leakage [109]. In this paper, we investigated an association between CSF protein levels and *APOE* ε4 carrier status and found significant differences between carriers and non-carriers in cognitively healthy controls. The protein concentrations of C9, CTSF, DPP7, and GM2A were decreased in the *APOE* ε4 carriers. No difference in protein expression was identified between ε4 carriers and non-carriers with AD; the differences were confined to the non-AD groups, a finding that needs to be confirmed in additional studies because of the relatively small sample size. As the increased risk of sporadic AD having an *APOE* ε4 genotype also relates to an earlier age of onset [107] additional investigation in a longitudinal cohort including subjects with prodromal AD would be preferable.

The present exploration includes the analysis of a large number of analytes in a restricted number of participants which might increase the risk of identifying false positives. However, four independent cohorts were analyzed resulting in the identification of a few repeatedly altered CSF protein concentrations. Not all protein concentrations were altered suggesting no general alteration in protein concentrations in any of the disorders. For proteins with altered concentrations between groups, in most cases, not all peptides originating from that protein had altered concentrations. There are several possible explanations for this discrepancy, for example, peptides belonging to different protein domains, endogenous protein fragments [110], or peptides being subjected to post-translational modifications. Also, no protein in the current investigation was specifically of neuronal origin.

The limited number of participants likely also affects the interpretation of the correlation analyses. For tryptic peptides originating from the same protein, correlations were typically high (Additional file 7: Table S4). A GM2A peptide (89–96) correlated with age in clinical study 1; however, for this peptide, there was no significant difference in concentration between groups. For the proteins identified at significantly altered levels between groups; AP2B1, LAMP2, and ubiquitin peptides correlated with P-tau. Ubiquitin is a component of tangles [86, 111, 112] and CSF P-tau does correlate with the amount of tangles in the brain [113]. How AP2B1 and LAMP2 relate to tangle formation remains elusive. We also found AP2B1 and ubiquitin to correlate with T-tau. The CSF concentration of T-tau is considered to reflect neuronal and axonal degeneration [114], a degeneration which might also explain a leakage of AP2B1 and ubiquitin. While T-tau and P-tau were found to correlate with AP2B1, LAMP2, and ubiquitin which might relate to the differences observed between prodromal AD and AD compared to controls and PD, the association with tau pathology is less clear for the non-correlating proteins CTSB, CTSF, and GM2A. Also, the association with tau pathology does not explain the differences observed between controls and PD (Fig. 4), for which there was no difference in the CSF concentration of T-tau and P-tau_181_ (Table 1). In PD, lower levels of lysosomal proteins in regions with α-synuclein pathology [18–20] might in part explain decreased concentrations in CSF. A pathway for intracellular endo-lysosomal proteins to reach the CSF might be facilitated by lysosomal exocytosis [115], exosomes [116], and degenerating and dying neurons. Lysosomal exocytosis [115] serves a purpose in membrane repair [117], myelination [118], and neurite outgrowth [119]. Lysosomal exocytosis [117] and neurite outgrowth [119] might act as a repair response in damaged neurons. By lysosomal exocytosis, through fusion with the plasma membrane, lysosomal hydrolases and contents are expelled into the extracellular milieu [115]. In the CSF, there are a great number of proteins [120, 121] and endogenous peptides [110]. Considering a fairly rigid and controlled path of CSF production at the main site, the *choroid plexus* [122], it might seem odd to find these proteins in the CSF. An additional source of CSF production is the drainage of parenchymal interstitial fluid into the CSF through interstitial fluid bulk flow [122, 123], a pathway that is however thought to be a slow process for diffusion of larger molecules [122, 123]. Additional pathways for soluble proteins in the brain parenchyma to reach the CSF might still be discovered. Extracellular vesicles in CSF, including exosomes, have been shown to be associated with several proteins investigated herein: APP, C9, CTSB, CTSD, CTSF, CTSL, GM2A, LYZ, TCN2, and ubiquitin [124]. Recently, neuronally derived exosomes in blood were found to contain elevated concentrations of CTSD, LAMP1, and ubiquitinated proteins already at a preclinical stage of AD, compared to controls and subjects with frontotemporal lobar degeneration [125].

## Conclusion

The present investigation revealed altered CSF concentrations of several proteins associated with the endo-lysosomal system and the ubiquitin-proteasome system in PD. Potentially, these altered concentrations reflect dysfunctional proteostasis, which has been suggested to be a pathological feature of PD [8]. Future studies are required to examine the biomarker evolvement during PD onset and progression, as well as how they relate to genetics. From a biomarker perspective, the involvement of lysosomal dysfunction in AD pathogenesis appears limited, at least as compared to PD.

## Additional files

Additional file 1: Supplementary Methods. (PDF 157 kb)

Additional file 2: **Table S1.** Proteins and peptides targeted using a combination of SPE and PRM-MS. (XLS 55 kb)

Additional file 3: **Table S2.** Proteins identified in CSF using tandem mass spectrometry. (XLS 2418 kb)

Additional file 4: **Figure S1.** Limit of quantification. (PDF 246 kb)

Additional file 5: **Figure S2.** Extracted ion chromatograms and MS/MS spectra. (PDF 4086 kb)

Additional file 6: **Table S3.** Demographics and biomarker data. (XLS 259 kb)

Additional file 7: **Table S4.** Spearman’s correlation analysis. (XLS 1583 kb)

Additional file 8: **Figure S3.** Significant peptides in clinical studies I and II. (PDF 218 kb)

Additional file 9: **Figure S4.** CSF protein concentrations in *APOE* ε4 carriers. (PDF 193 kb)

## Data Availability

The data that support the findings of this paper are available in the published article and its additional files as well as in publically available data repositories. The explorative mass spectrometry proteomics data have been deposited to the ProteomeXchange Consortium via the PRIDE [126] partner repository with the dataset identifier PXD012851 (http://proteomecentral.proteomexchange.org/cgi/GetDataset?ID=PXD012851). Quantitative data have received the dataset identifier PXD013451 (http://proteomecentral.proteomexchange.org/cgi/GetDataset?ID=PXD013451) and been made available at Panorama Public [127] (https://panoramaweb.org/PRM-MS_EndoLys_ADPD.url).

## Abbreviations

Aβ: Amyloid β peptide
Aβ_1–42_: 42 amino acid long amyloid β peptide
AD: Alzheimer’s disease
CSF: Cerebrospinal fluid
HCD: Higher-energy collisional dissociation
LOQ: Limit of quantification
MCI: Mild cognitive impairment
MS/MS: Tandem mass spectrometry
NINDS: National Institute of Neurological Disorders and Stroke
PD: Parkinson’s disease
PRM-MS: Parallel reaction monitoring mass spectrometry
P-tau: Hyperphosphorylated tau protein
P-tau_181_: Tau protein phosphorylated at Thr181
SPE: Solid-phase extraction
T-tau: Total tau protein
QC: Quality control CSF pool
