# Supplementary material for: Endo-lysosomal proteins and ubiquitin CSF concentrations in Alzheimer’s and Parkinson’s disease
Source: Alzheimers Res Ther. 2019 Sep 14;11:82. doi: 10.1186/s13195-019-0533-9 (PMC6745076; doi:10.1186/s13195-019-0533-9)
Supplement: Supplementary file 1 — Supplementary Methods. (PDF 157 kb) [file 13195_2019_533_MOESM1_ESM.pdf]

Additional File 1

Endo-Lysosomal Proteins and Ubiquitin CSF Concentrations in Alzheimer's and Parkinson's Disease

Simon Sjödin[1,2], Gunnar Brinkmalm[1,2], Annika Öhrfelt[1,2], Lucilla Parnetti[3], Silvia Paciotti[4], Oskar Hansson[5,6], John Hardy[7], Kaj Blennow[1,2], Henrik Zetterberg[1,2,7,8], Ann Brinkmalm[1,2]

[1]Department of Psychiatry and Neurochemistry, Institute of Neuroscience and Physiology, the Sahlgrenska Academy at the University of Gothenburg, Mölndal, Sweden

[2]Clinical Neurochemistry Laboratory, Sahlgrenska University Hospital, Mölndal, Sweden

[3]Neurology Clinic, University of Perugia, Perugia, Italy

[4]Department of Experimental Medicine, University of Perugia, Perugia, Italy

[5]Clinical Memory Research Unit, Department of Clinical Sciences Malmö, Lund University, Lund, Sweden

[6]Memory Clinic, Skåne University Hospital, Malmö, Sweden

[7]Department of Molecular Neuroscience, University College London Institute of Neurology, Queen Square, London, UK

[8]UK Dementia Research Institute at UCL, London, United Kingdom

Corresponding Author: Simon Sjödin, Department of Psychiatry and Neurochemistry, Institute of Neuroscience and Physiology, the Sahlgrenska Academy at the University of Gothenburg, House V3, SU/Mölndal, SE-43180, Mölndal, Sweden. simon.sjodin@neuro.gu.se.

Content

Supplementary Methods

Supplementary Methods

Protein and Stable Isotope-Labeled Standards

Crude stable isotope-labeled tryptic peptides, labeled with C-terminal heavy Lys or Arg ($^{13}C/^{15}N$ labeled) and modified by Cys carbamidomethylation, were purchased from JPT Peptide Technologies GmbH (SpikeTides L; Berlin, Germany) and Thermo Fisher Scientific Inc. (FasTrack 1; Waltham, MA, USA). The peptides are listed in Supplementary Table 1. Peptides from JPT Peptide Technologies GmbH were dissolved in 10% acetonitrile to a concentration of 13 μM. Peptides from Thermo Fisher Scientific Inc. were dissolved accordingly in H_2O; BSA_421-433 (bovine serum albumin; 170 μM), C9_146-154 (290 μM), C9_186-194 (450 μM), C9_232-242 (170 μM), C9_473-483 (240 μM), LAMP2_133-144 (76 μM), LAMP2_153-161 (280 μM) or in 23% acetonitrile and 0.76% formic acid; C9_497-508 (240 μM). All stable isotope-labeled peptides were frozen and stored at −20° C. Also bovine serum albumin (BSA; full length, average mass 66430 Da; 100% purity by agarose electrophoresis; Sigma-Aldrich Co. Saint Louis, MO, USA) and ^{13}C labeled ubiquitin (^{13}C-ubiquitin; average mass 8940 Da; >90% protein purity by SDS electrophoresis and >98% isotope enrichment purity; Silantes, GmbH, München, Germany) were dissolved in H_2O to a concentration of 200 μM and 100 μM, respectively. BSA and ^{13}C-ubiquitin were frozen and stored at −20° C and −80° C, respectively. An internal standard mixture of stable isotope-labeled peptides, BSA and ^{13}C-ubiquitin was prepared in 50 mM NH_4HCO_3 for the addition to CSF samples. The concentration of peptides and proteins in this mixture is shown in Supplementary Table 1. The standard solution was aliquoted, frozen and stored at −80° C. Similarly, a mixture of stable isotope-labeled peptides and ^{13}C-ubiquitin was prepared in 50 mM NH_4HCO_3 for reverse calibration curves by serially diluting the mixture. After dilution BSA was added to all reverse calibration point dilutions to a final concentration of 330 nM BSA. The mixtures of reverse calibration points were frozen and stored at −80° C.

Protein Identification by MS/MS

Sample digestion and SPE was performed as described in the manuscript with a minor modification. Alkylation was performed with the addition of 25 µL of 14 mM iodoacetamide (Sigma-Aldrich Co.) in 50 mM NH_4HCO_3 followed by 30 min shaking incubation at room temperature in dark.

Digested samples were dissolved in 100 µL of 50 mM NH_4HCO_3, 0.1% formic acid or 20% acetonitrile by shaking at room temperature for 1 h. Six microliters of dissolved samples were injected and separated using a Dionex UltiMate 3000 nano-LC system (Thermo Fisher Scientific Inc.) with an Acclaim PepMap 100 nanoViper C18 trap column (length, 20 mm; inner diameter, 75 µm; particle size, 3 µm; Thermo Fisher Scientific Inc.) and an Acclaim PepMap RSLC nanoViper C18 column (length, 500 mm; inner diameter, 75 µm; particle size, 2 µm; Thermo Fisher Scientific Inc.). Mobile phases used were; A: 0.1 % formic acid in water (v/v); and B: 0.1 % formic acid and 84 % acetonitrile in water (v/v). Liquid chromatography was performed in online mode coupled to a tribrid Orbitrap Fusion mass spectrometer (Thermo Fisher Scientific Inc.) operating in positive mode with a nano-spray probe (NSI). The samples were analyzed on two different occasions applying slightly different methodological approaches.

First, triplicate samples dissolved in 0.1% formic acid was separated at a flow rate of 150 nL/min, at +40° C, going from 5% to 40% B over 230 min. The spray voltage was set to 1.7 kV and the capillary temperature to +275° C. A top speed method with 3 sec cycle times using the orbitrap as analyzer was employed. Survey scans were acquired at a resolution setting of 60 k, using a scan range of *m/z* 350-1400, an AGC target of 2×10^5, a maximum injection time of 50 ms, collecting single microscans and a dynamic exclusion for 50 sec. This was followed by inclusion of the *most* intense precursor ions with an intensity greater than 5×10^4 and a charge state range of *z* 2-8 for tandem mass spectrometry (MS/MS). MS/MS was

achieved by acquisition of single microscans using an isolation window of *m/z* 3, higher-energy collisional dissociation (HCD) fragmentation with an normalized collision energy (NCE) setting of 29%, a resolution setting of 15 k, a scan range of *m/z* 350-1000, an AGC target of 5×10^4 and a maximum injection time of 60 ms. Second, triplicate samples dissolved in 50 mM NH_4HCO_3, 0.1% formic acid or 20% acetonitrile were separated at a flow rate of 150 nL/min, at +60° C, going from 5% to 40% B over 230 min. The spray voltage was set to 1.7 kV and the capillary temperature to +250° C. Similarly as described above, a top speed method with 3 sec cycle times and using the orbitrap as analyzer was used. Following survey scans, the *most* intense ions with an intensity greater than 1×10^4 and a charge state range of *z* 2-8 were targeted for MS/MS using the same settings as described above. To enhance data coverage, a second injection of the very same samples were performed with the inclusion for MS/MS of the *least* intense ions with an intensity greater than 1×10^4 using the settings already described.

Bioinformatic Analysis

Acquired spectra were searched using an in-house Mascot database server v2.6.1 (Matrix Science Ltd. London, UK), facilitated by Thermo Proteome Discoverer v2.1.1.21 (Thermo Fisher Scientific Inc.). The following search parameters were used: database, SwissProt (2017-11-07, sequences 42153, residues 24298601); taxonomy, *homo sapiens*; enzyme, trypsin; maximum missed cleavages, one; dynamic modification, methionine oxidation; static modification, cysteine carbamidomethylation; instrument type/fragmentation type, 1+, 2+, b- and y-ions; peptide mass tolerance, 10 ppm; and fragment mass tolerance, 20 mmu. Percolator was used for scoring peptide matches using a false discovery rate threshold of 1%. Samples injected twice, using the *most* intense and the *least* intense ions method, respectively, were combined using MudPit scoring.
