# Supplementary material for: Endo-lysosomal proteins and ubiquitin CSF concentrations in Alzheimer’s and Parkinson’s disease
Source: Alzheimers Res Ther. 2019 Sep 14;11:82. doi: 10.1186/s13195-019-0533-9 (PMC6745076; doi:10.1186/s13195-019-0533-9)
Supplement: Supplementary file 4 — Figure S1. Limit of quantification. (PDF 246 kb) [file 13195_2019_533_MOESM4_ESM.pdf]

Additional File 4

Endo-Lysosomal Proteins and Ubiquitin CSF Concentrations in Alzheimer's and Parkinson's Disease

Simon Sjödin[1,2], Gunnar Brinkmalm[1,2], Annika Öhrfelt[1,2], Lucilla Parnetti[3], Silvia Paciotti[4], Oskar Hansson[5,6], John Hardy[7], Kaj Blennow[1,2], Henrik Zetterberg[1,2,7,8], Ann Brinkmalm[1,2]

[1]Department of Psychiatry and Neurochemistry, Institute of Neuroscience and Physiology, the Sahlgrenska Academy at the University of Gothenburg, Mölndal, Sweden

[2]Clinical Neurochemistry Laboratory, Sahlgrenska University Hospital, Mölndal, Sweden

[3]Neurology Clinic, University of Perugia, Perugia, Italy

[4]Department of Pharmaceutical Sciences, University of Perugia, Perugia, Italy

[5]Clinical Memory Research Unit, Department of Clinical Sciences Malmö, Lund University, Lund, Sweden

[6]Memory Clinic, Skåne University Hospital, Malmö, Sweden

[7]Department of Molecular Neuroscience, University College London Institute of Neurology, Queen Square, London, UK

[8]UK Dementia Research Institute at UCL, London, United Kingdom

Corresponding Author: Simon Sjödin, Department of Psychiatry and Neurochemistry, Institute of Neuroscience and Physiology, the Sahlgrenska Academy at the University of Gothenburg, House V3, SU/Mölndal, SE-43180, Mölndal, Sweden. simon.sjodin@neuro.gu.se.

Content

Figure S1

Figure S1. Limit of quantification. Six points reversed calibration curves were created by diluting stable isotope labeled peptides and adding these dilutions to a quality control CSF pool sample. The limit of quantification was calculated using least square linear regression and determined as the concentration range with a relative error of calculated to nominal concentration ≤20%. The concentration range is shown in the figure for all peptides analyzed, A-Ay, and a line has been fitted using least square linear regression. Duplicate calibration curves were analyzed in three experiments. The mean of the duplicates are plotted showing the mean and SD for the three experiments.

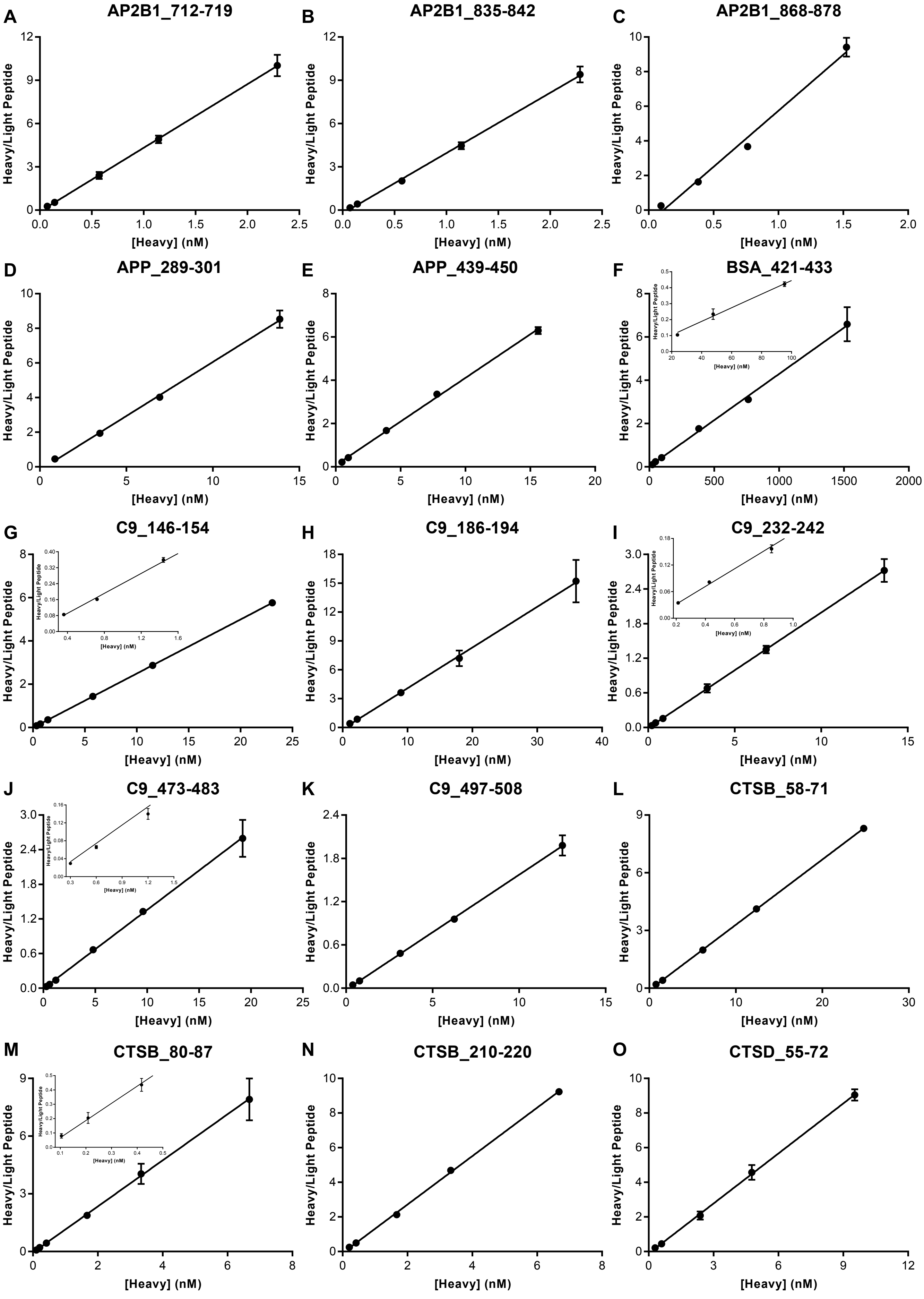

A
AP2B1_712-719
B
AP2B1_835-842
C
AP2B1_868-878
D
APP_289-301
E
APP_439-450
F
BSA_421-433
G
C9_146-154
H
C9_186-194
I
C9_232-242
J
C9_473-483
K
C9_497-508
L
CTSB_58-71
M
CTSB_80-87
N
CTSB_210-220
O
CTSD_55-72
Heavy/Light Peptide
[Heavy] (nM)

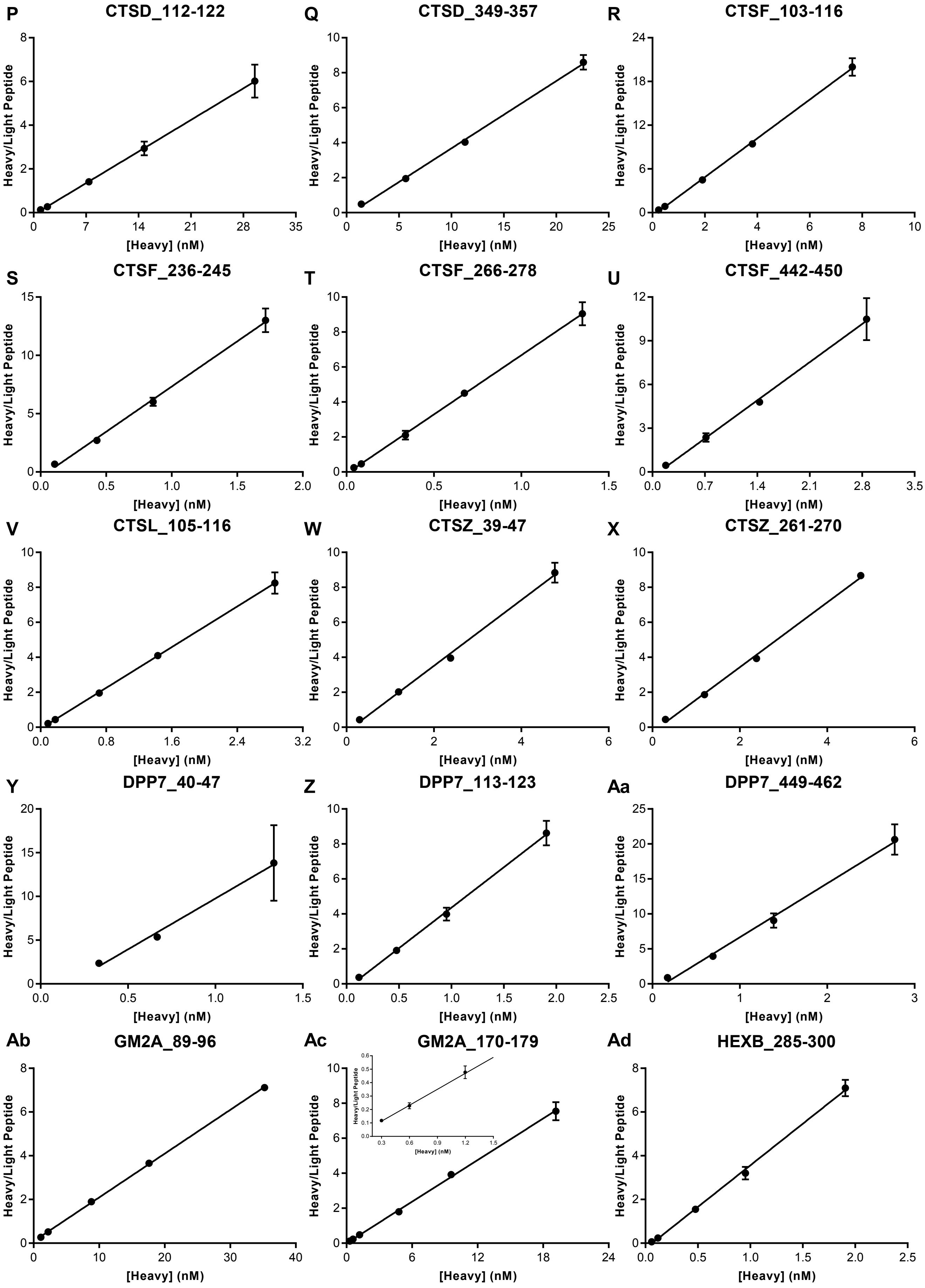
P
CTSD_112-122
Q
CTSD_349-357
R
CTSF_103-116
S
CTSF_236-245
T
CTSF_266-278
U
CTSF_442-450
V
CTSL_105-116
W
CTSZ_39-47
X
CTSZ_261-270
Y
DPP7_40-47
Z
DPP7_113-123
Aa
DPP7_449-462
Ab
GM2A_89-96
Ac
GM2A_170-179
Ad
HEXB_285-300
Heavy/Light Peptide
[Heavy] (nM)

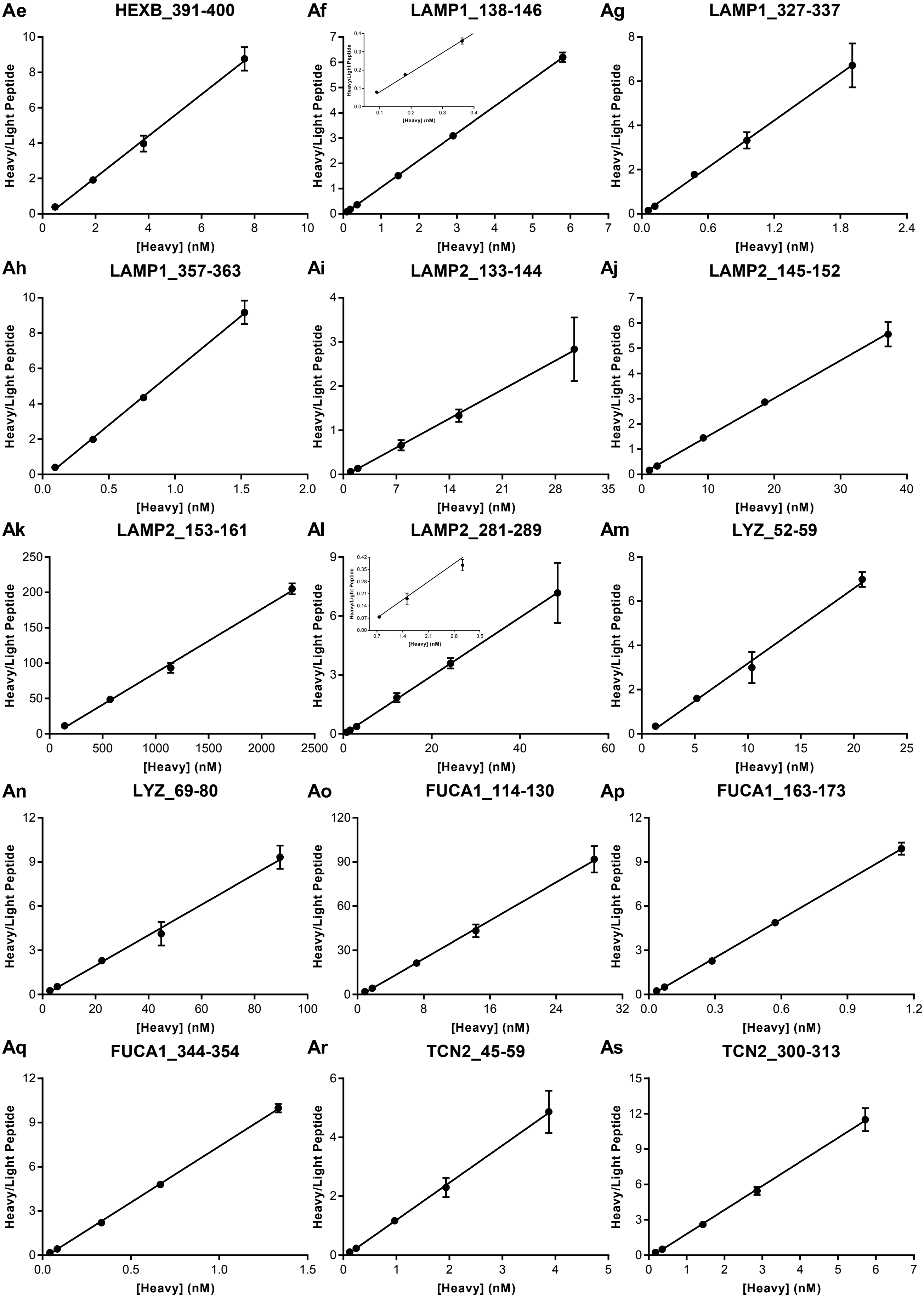

Ae
HEXB_391-400
Af
LAMP1_138-146
Ag
LAMP1_327-337
Ah
LAMP1_357-363
Ai
LAMP2_133-144
Aj
LAMP2_145-152
Ak
LAMP2_153-161
Al
LAMP2_281-289
Am
LYZ_52-59
An
LYZ_69-80
Ao
FUCA1_114-130
Ap
FUCA1_163-173
Aq
FUCA1_344-354
Ar
TCN2_45-59
As
TCN2_300-313
Heavy/Light Peptide
[Heavy] (nM)

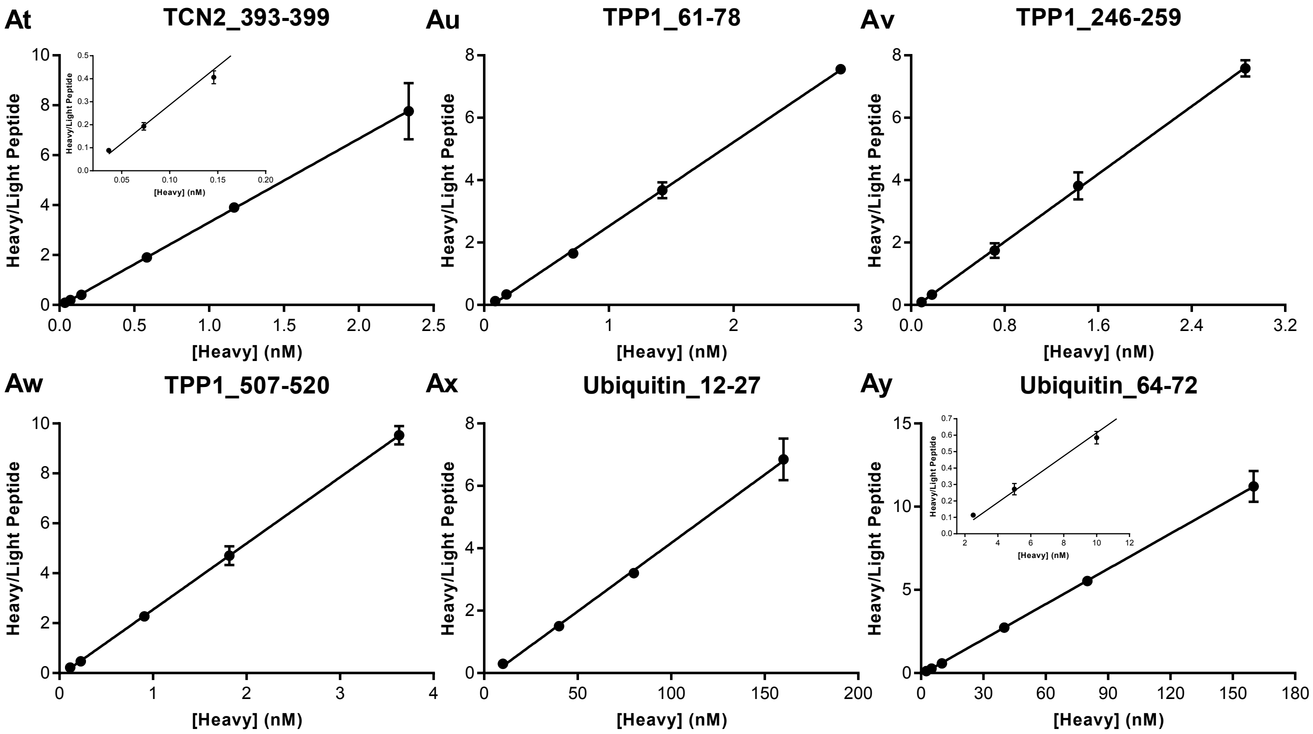

At
TCN2_393-399
Au
TPP1_61-78
Av
TPP1_246-259
Aw
TPP1_507-520
Ax
Ubiquitin_12-27
Ay
Ubiquitin_64-72
Heavy/Light Peptide
[Heavy] (nM)
