# Supplementary material for: Endo-lysosomal proteins and ubiquitin CSF concentrations in Alzheimer’s and Parkinson’s disease
Source: Alzheimers Res Ther. 2019 Sep 14;11:82. doi: 10.1186/s13195-019-0533-9 (PMC6745076; doi:10.1186/s13195-019-0533-9)

Additional File 5

Endo-Lysosomal Proteins and Ubiquitin CSF Concentrations in Alzheimer's and Parkinson's Disease

Simon Sjödin[1,2], Gunnar Brinkmalm[1,2], Annika Öhrfelt[1,2], Lucilla Parnetti[3], Silvia Paciotti[4], Oskar Hansson[5,6], John Hardy[7], Kaj Blennow[1,2], Henrik Zetterberg[1,2,7,8], Ann Brinkmalm[1,2]

[1]Department of Psychiatry and Neurochemistry, Institute of Neuroscience and Physiology, the Sahlgrenska Academy at the University of Gothenburg, Mölndal, Sweden

[2]Clinical Neurochemistry Laboratory, Sahlgrenska University Hospital, Mölndal, Sweden

[3]Neurology Clinic, University of Perugia, Perugia, Italy

[4]Department of Pharmaceutical Sciences, University of Perugia, Perugia, Italy

[5]Clinical Memory Research Unit, Department of Clinical Sciences Malmö, Lund University, Lund, Sweden

[6]Memory Clinic, Skåne University Hospital, Malmö, Sweden

[7]Department of Molecular Neuroscience, University College London Institute of Neurology, Queen Square, London, UK

[8]UK Dementia Research Institute at UCL, London, United Kingdom

Corresponding Author: Simon Sjödin, Department of Psychiatry and Neurochemistry, Institute of Neuroscience and Physiology, the Sahlgrenska Academy at the University of Gothenburg, House V3, SU/Mölndal, SE-43180, Mölndal, Sweden. simon.sjodin@neuro.gu.se.

Content

Figure S2

Figure S2. Extracted ion chromatograms and MS/MS spectra. Shown are examples of extracted ion chromatograms (top panels) and MS/MS spectra (bottom panels) acquired by PRM-MS for all peptides targeted, A-Ay. Light indicate the tryptic peptide of endogenous origin and Heavy is the added stable isotope-labeled peptide.

A; AP2B1_712-719; AVWLPAVK

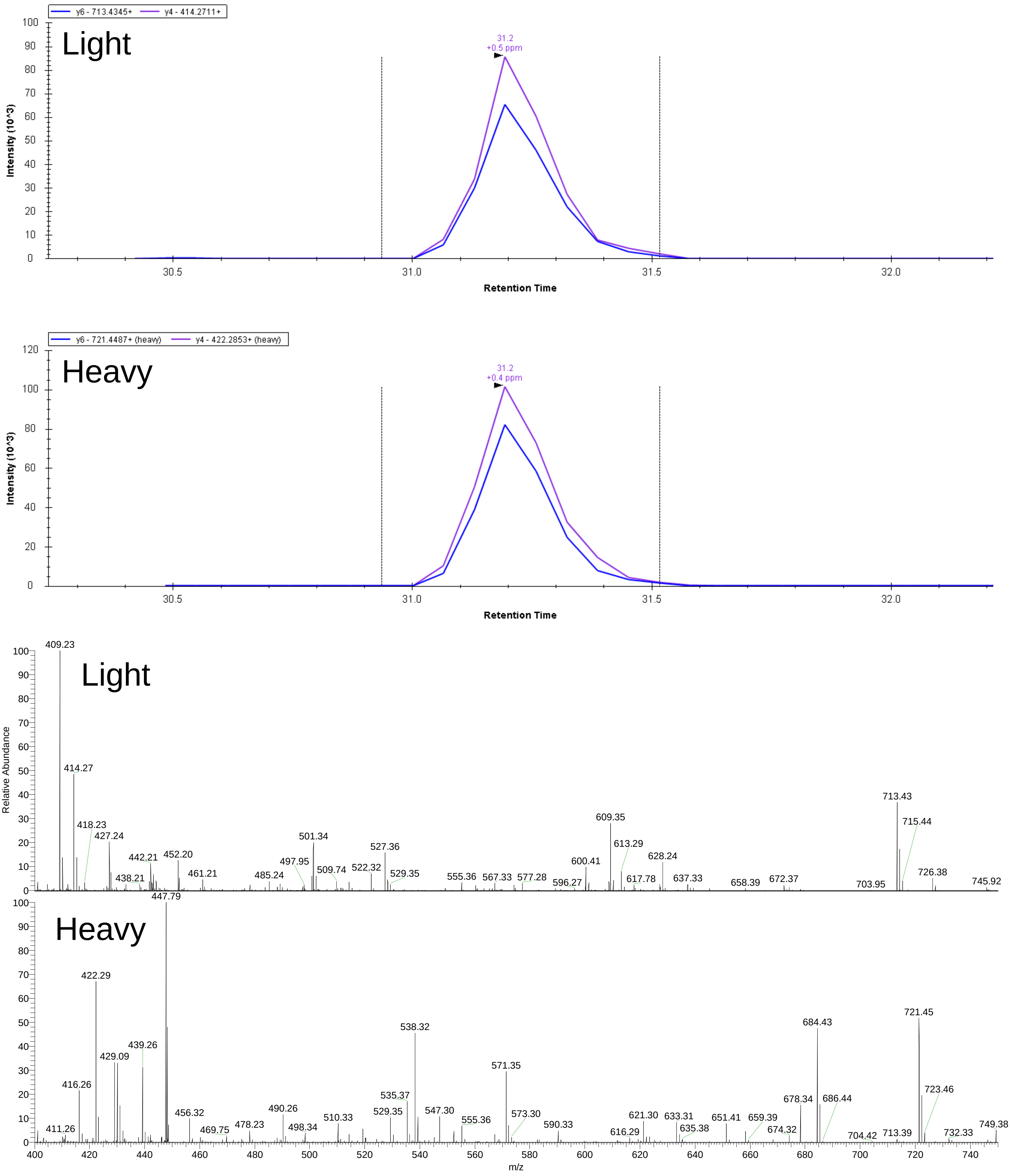

B; AP2B1_835-842; QVFLATWK

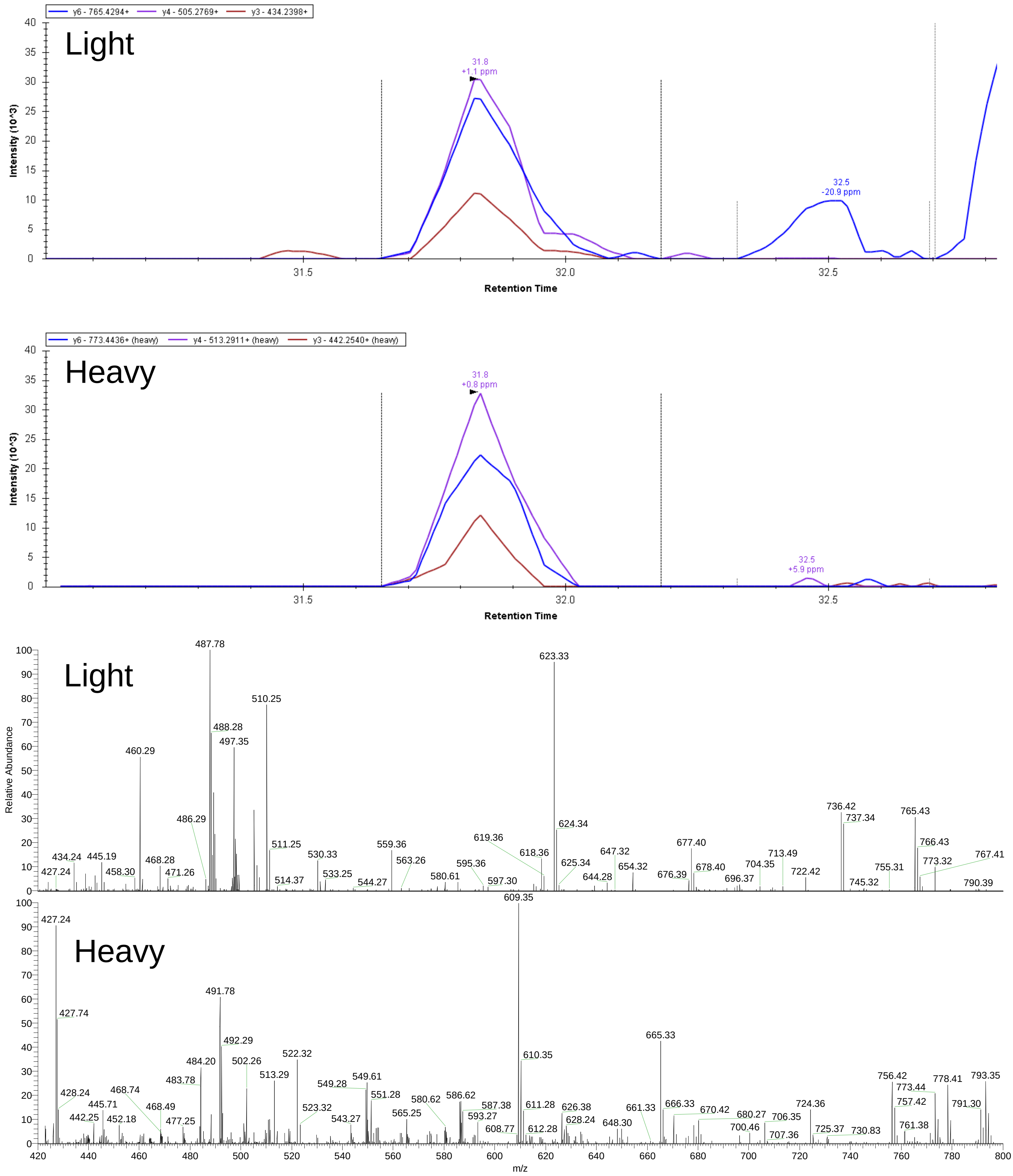

C; AP2B1_868-878; LQNNNVYTIAK

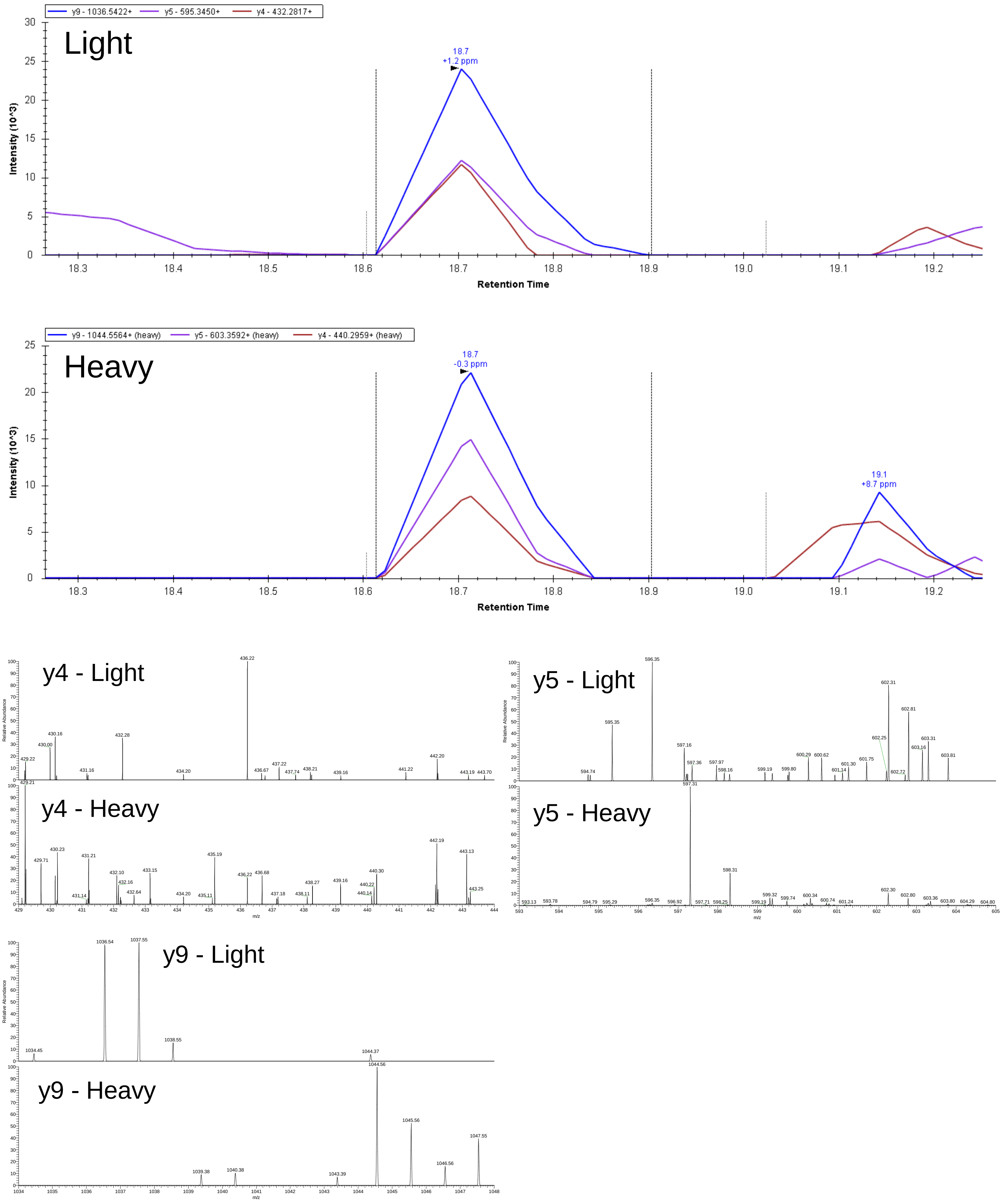

D; APP_289-301; EVcSEQAETGPcR

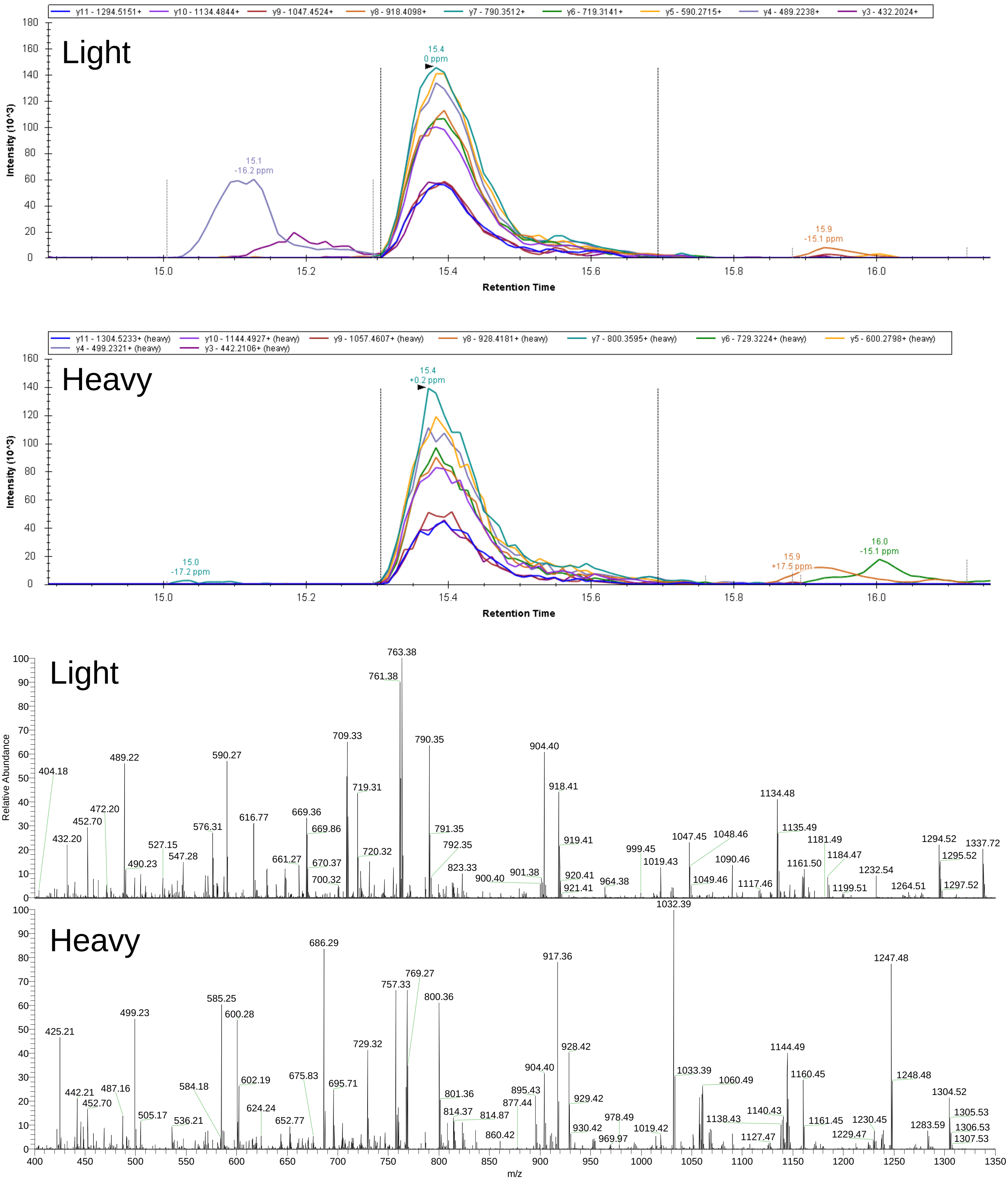

E; APP_439-450; VESLEQEAANER

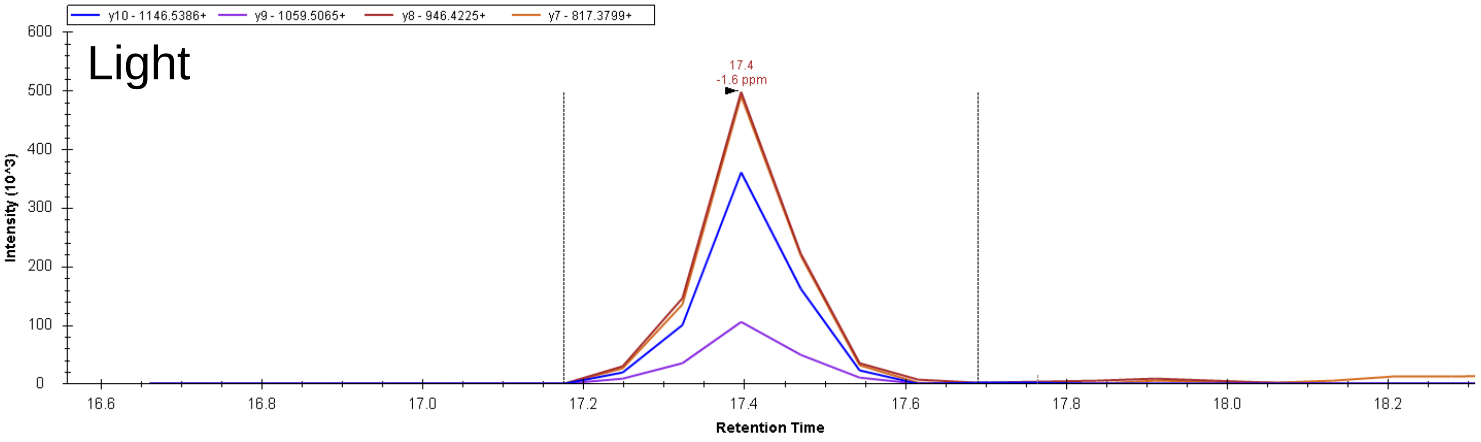

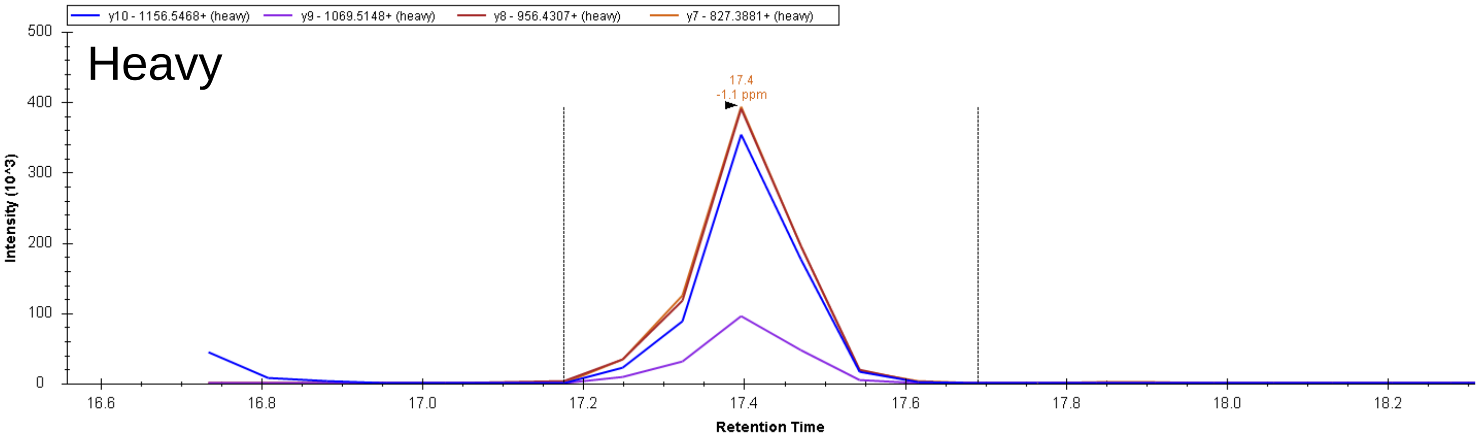

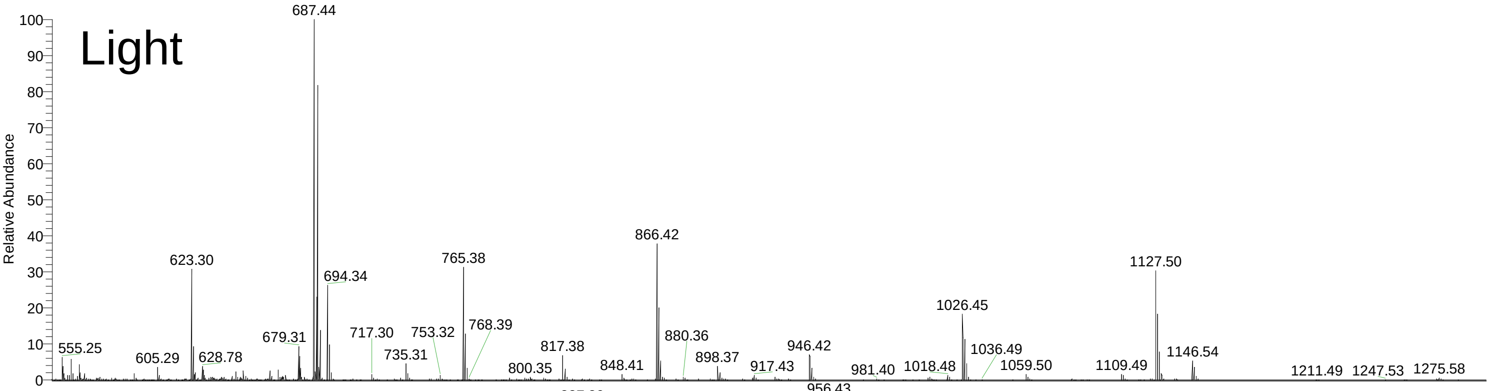

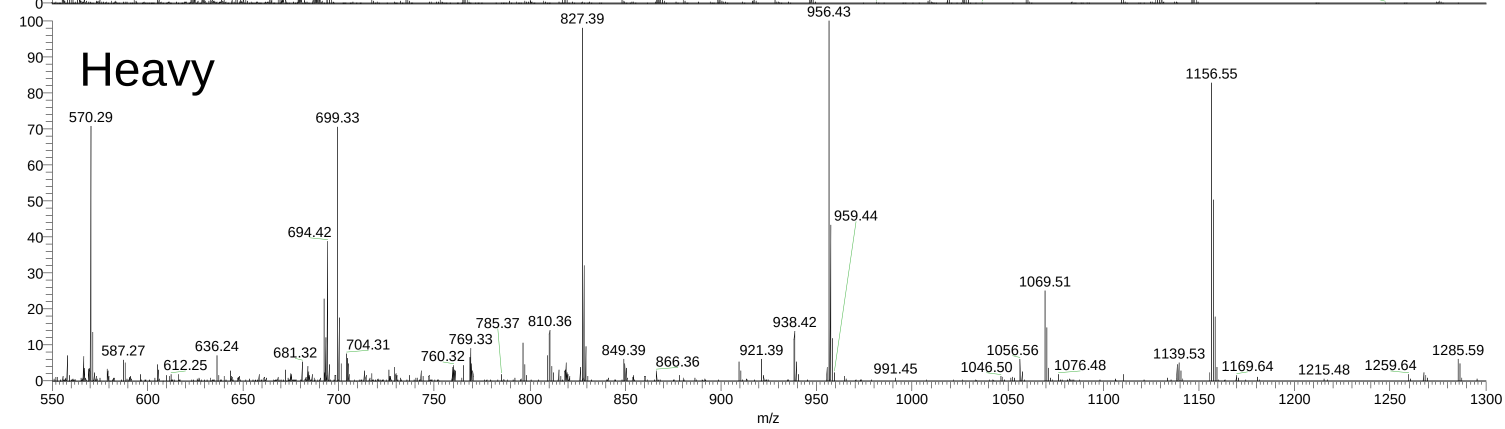

F; BSA_421-433; LGEYGFQNALIVR

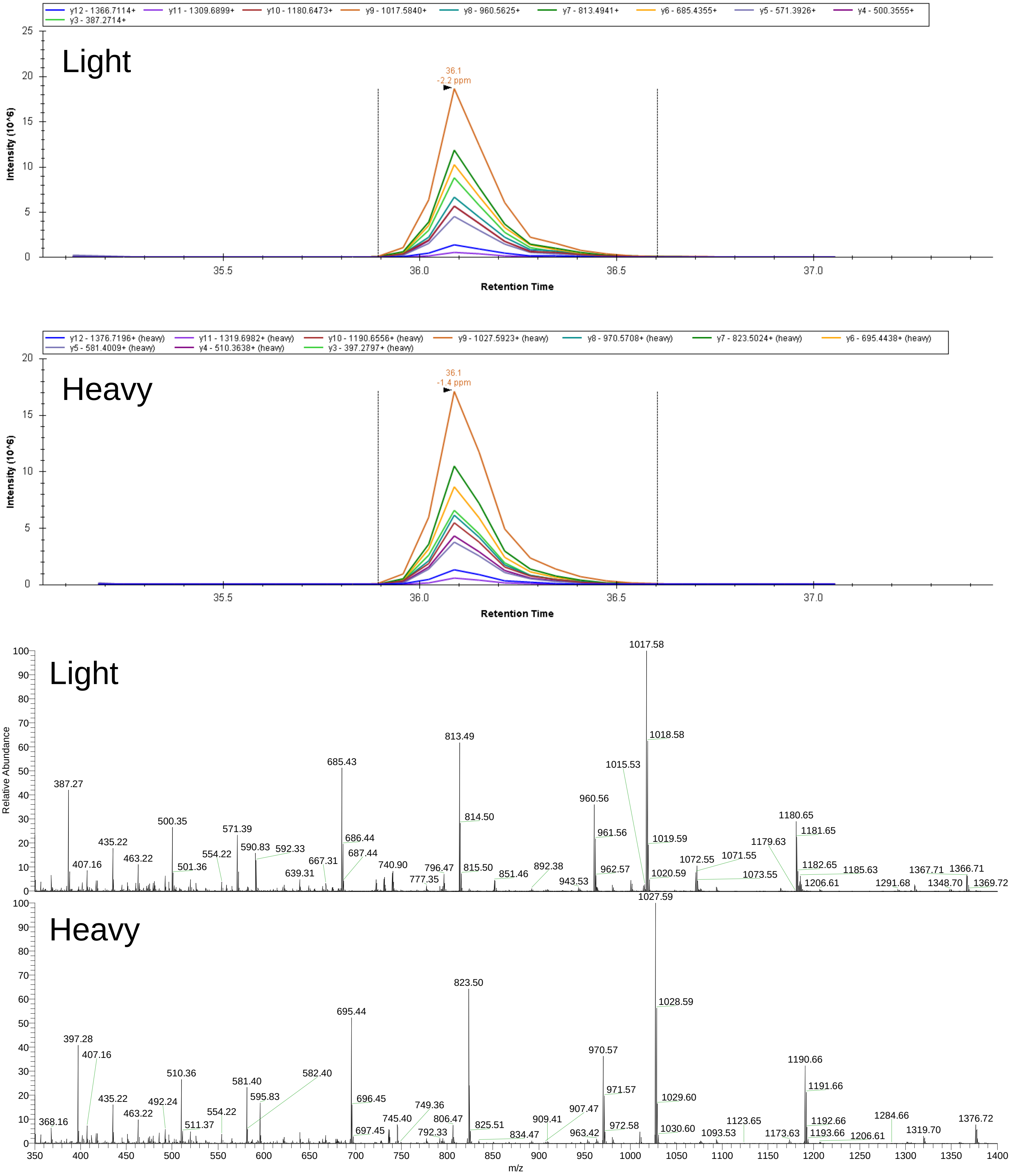

G; C9_146-154; VVEESELAR

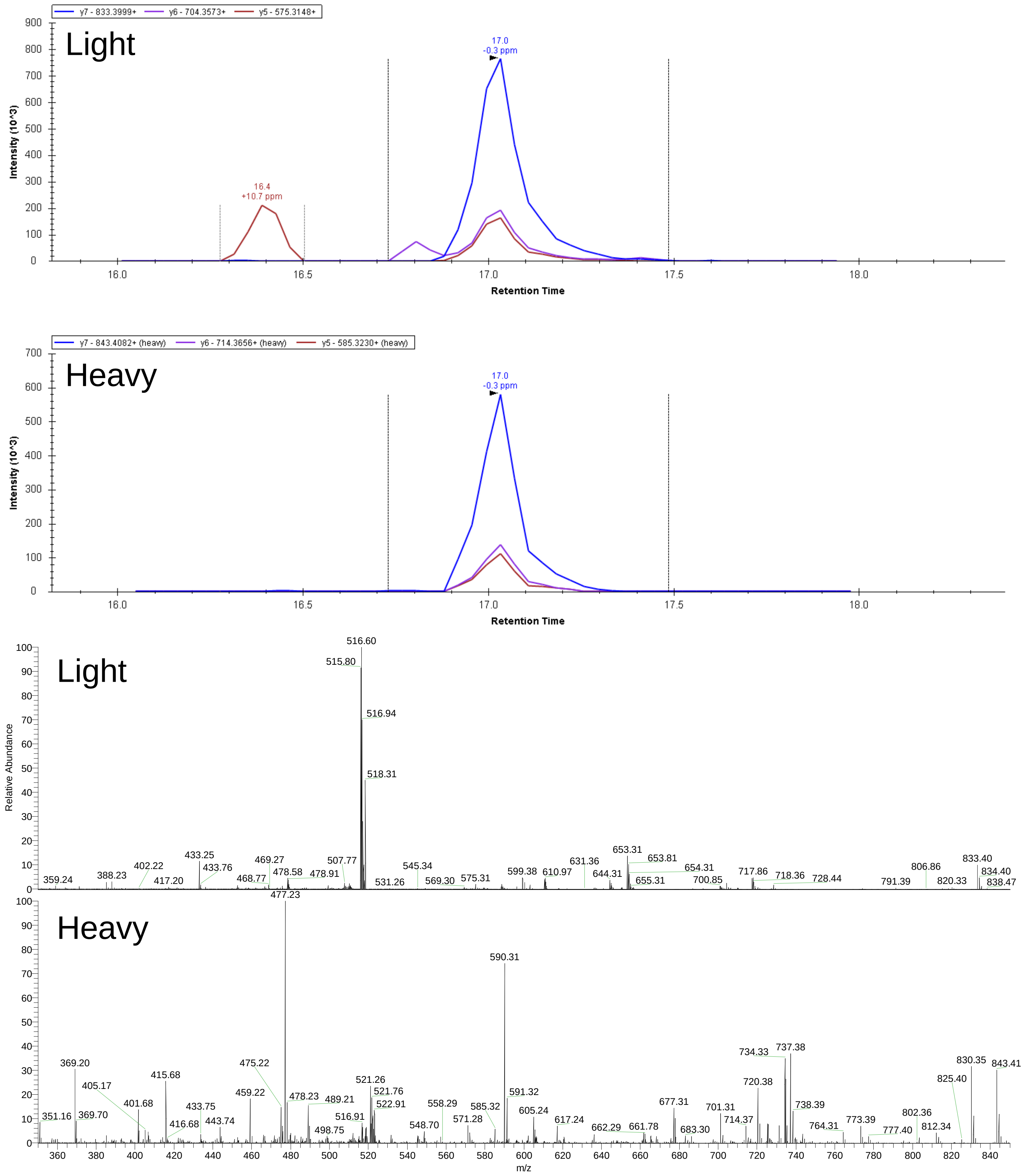

H; C9_186-194; DGNTLTYYR

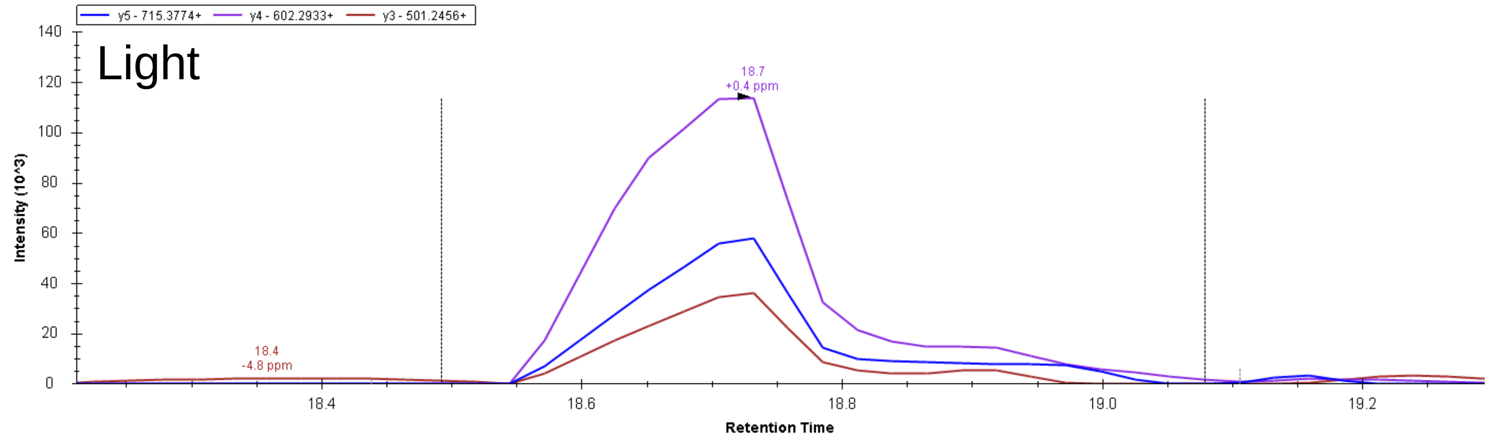

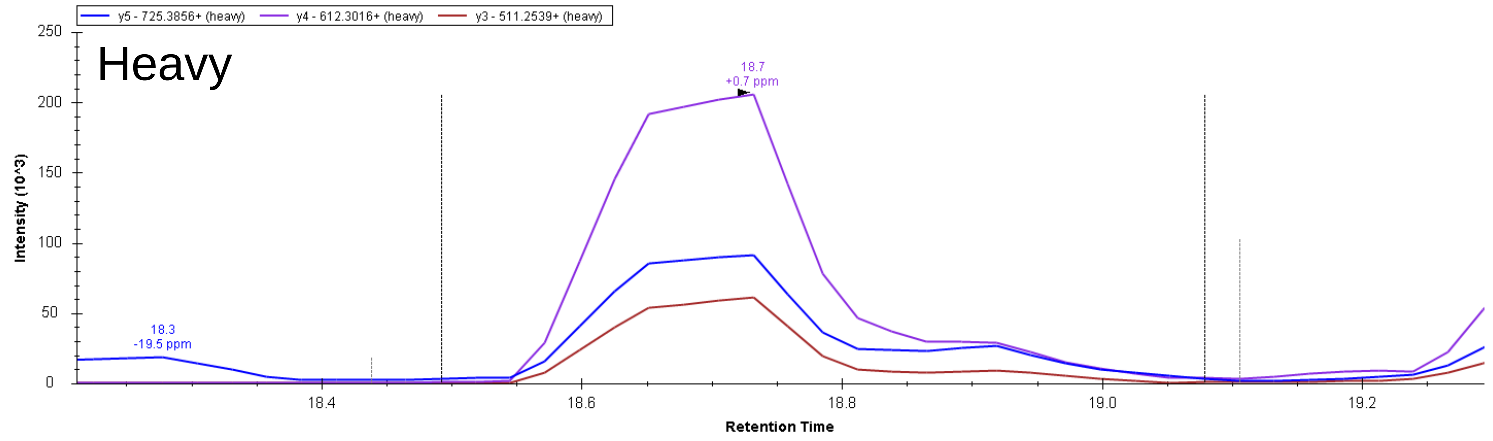

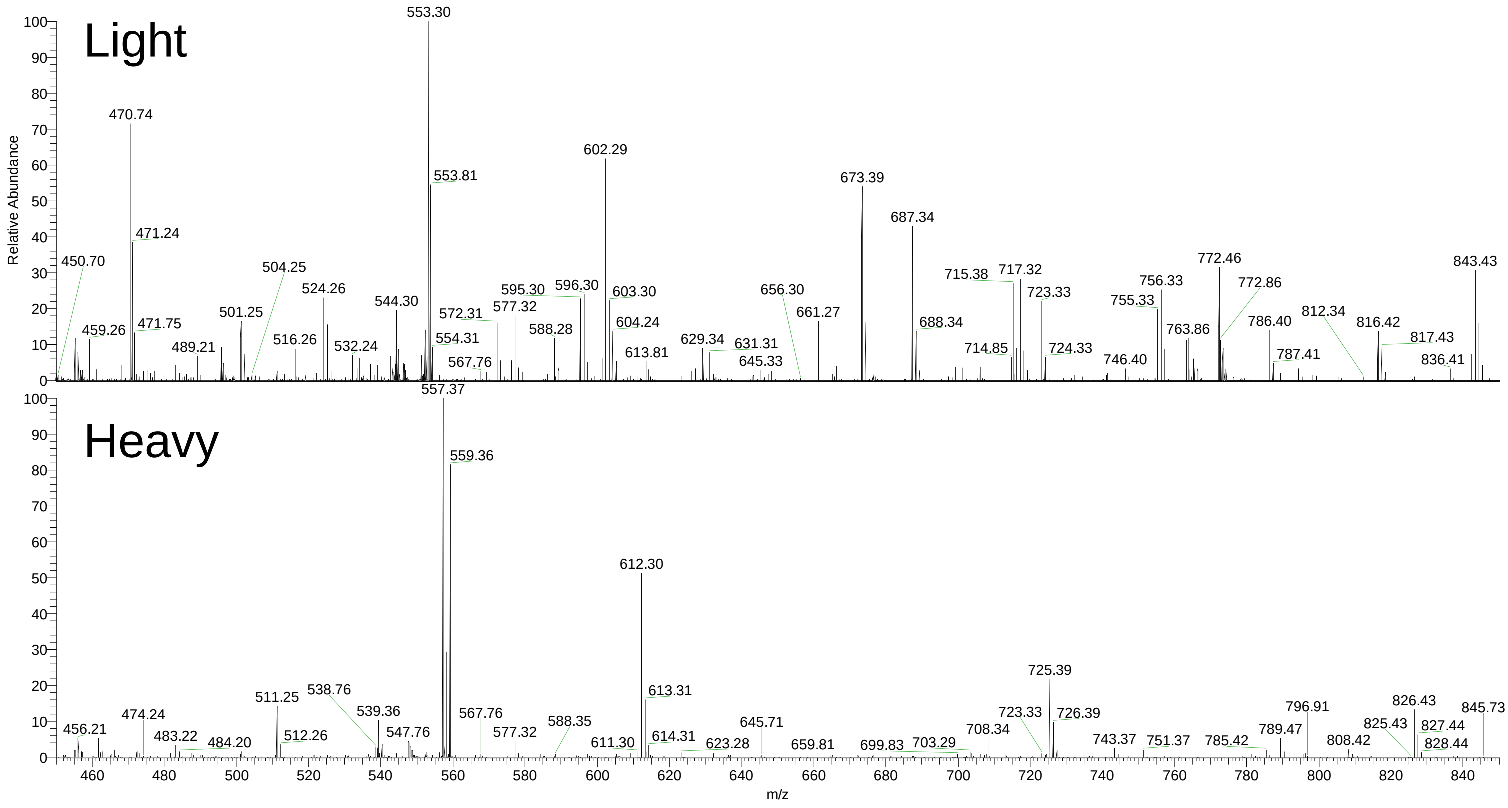

I; C9_232-242; TSNFNAAISLK

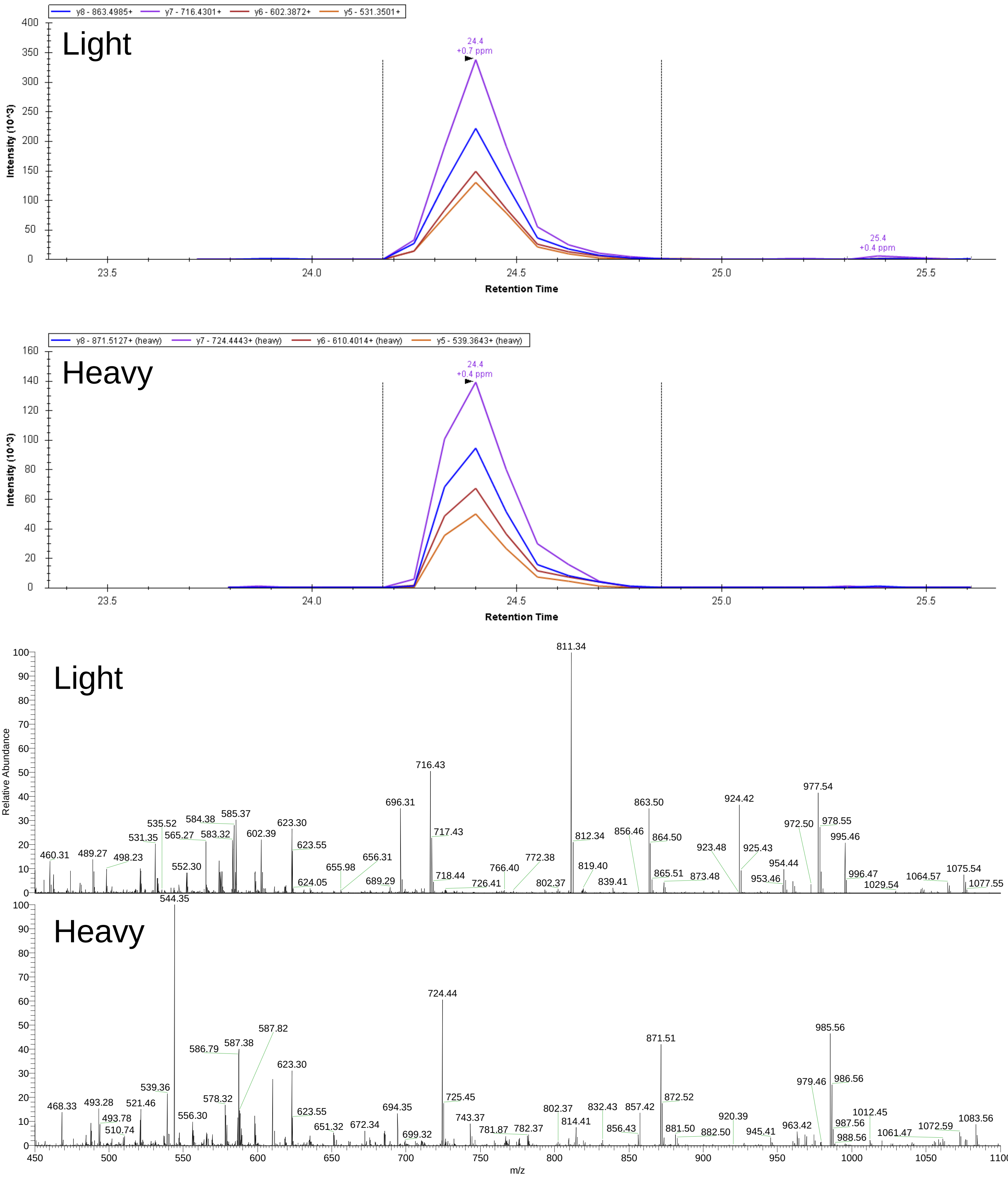

J; C9_473-483; LSPIYNLVPVK

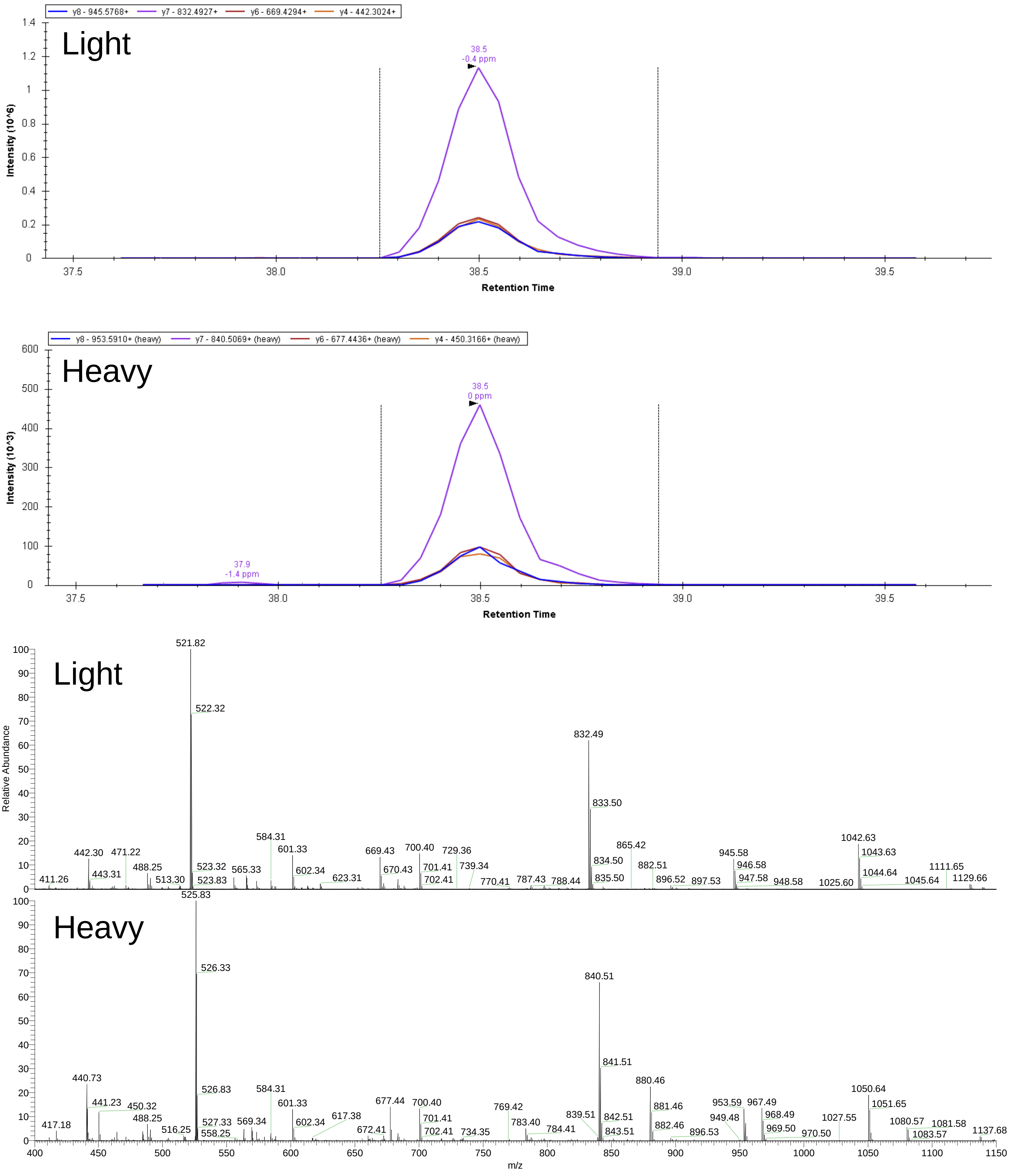

K; C9_497-508; AIEDYINEFSVR

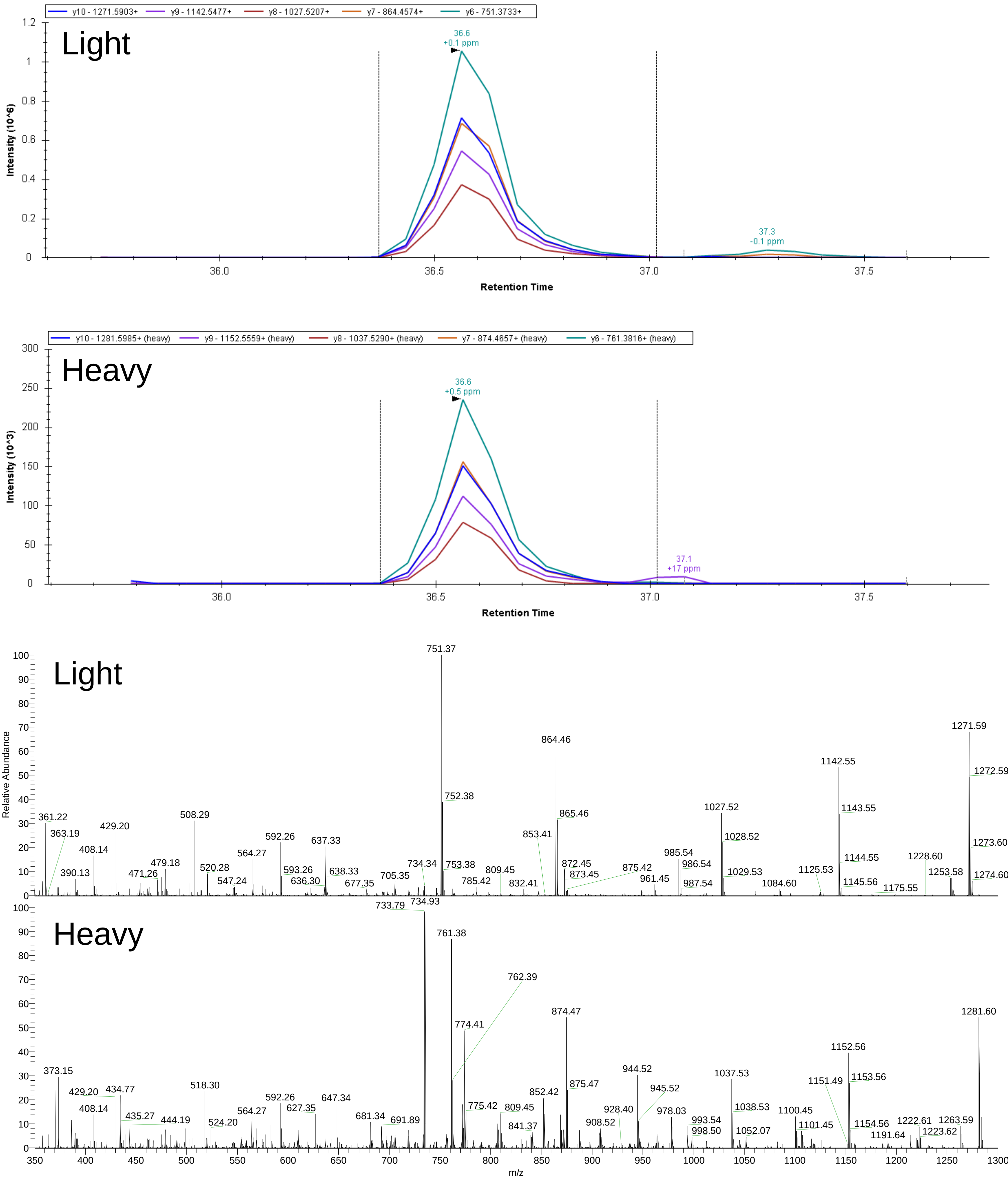

L; CTSB_58-71; LcGTFLGGPKPPQR

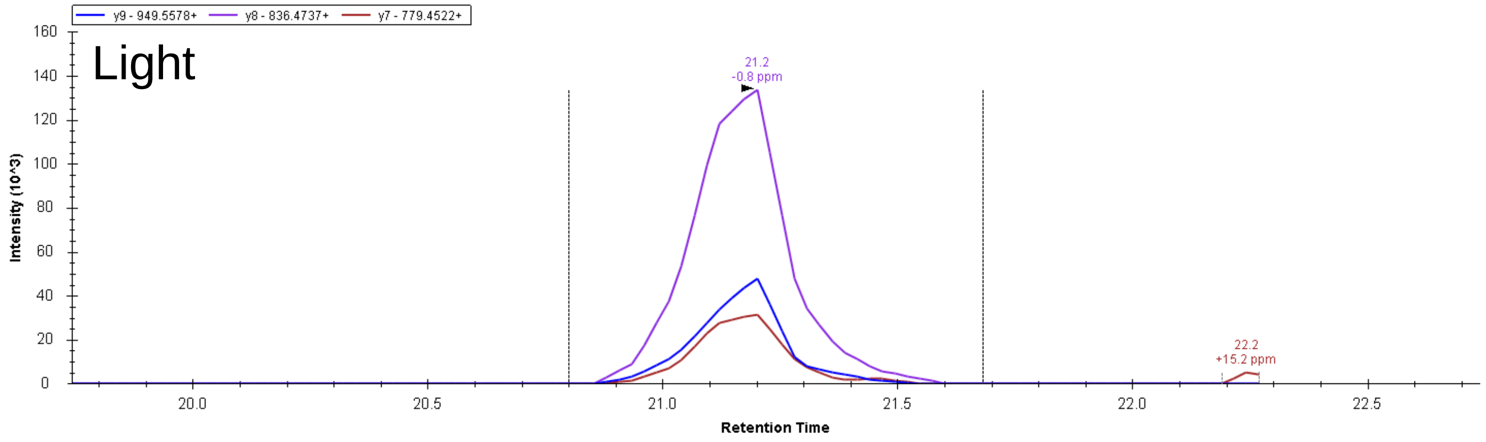

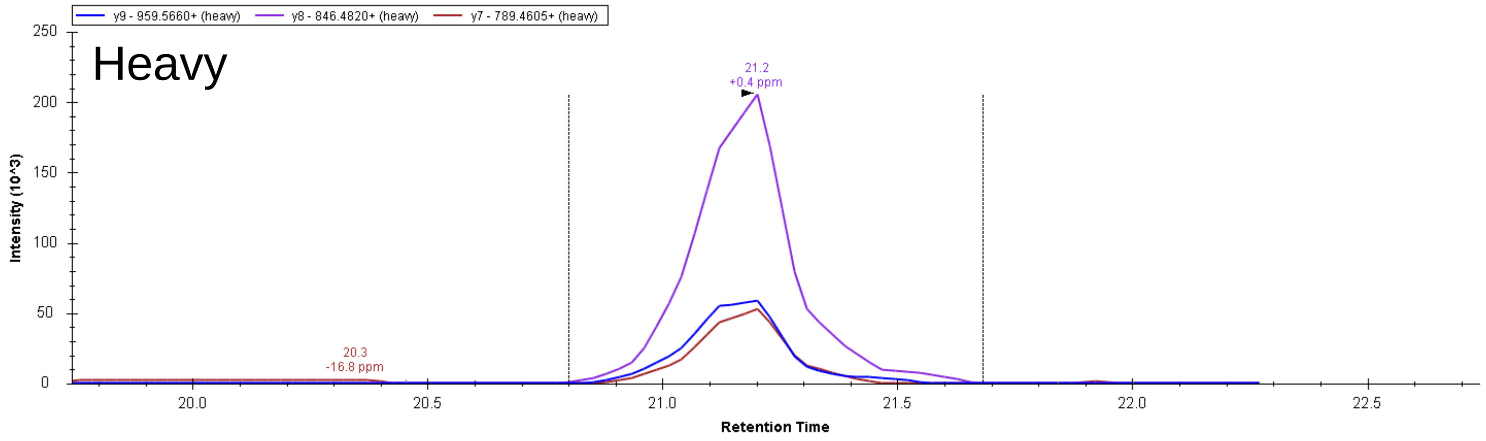

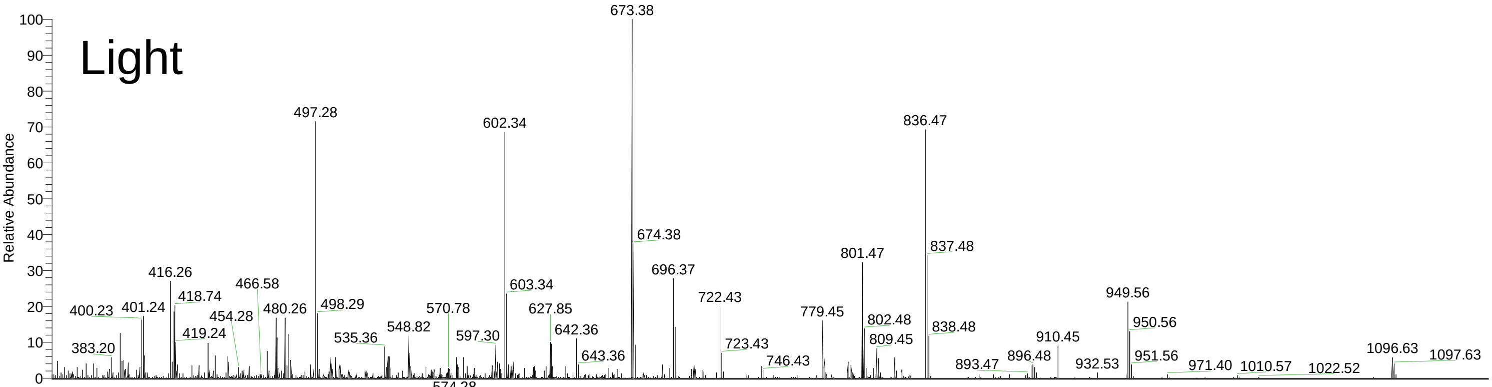

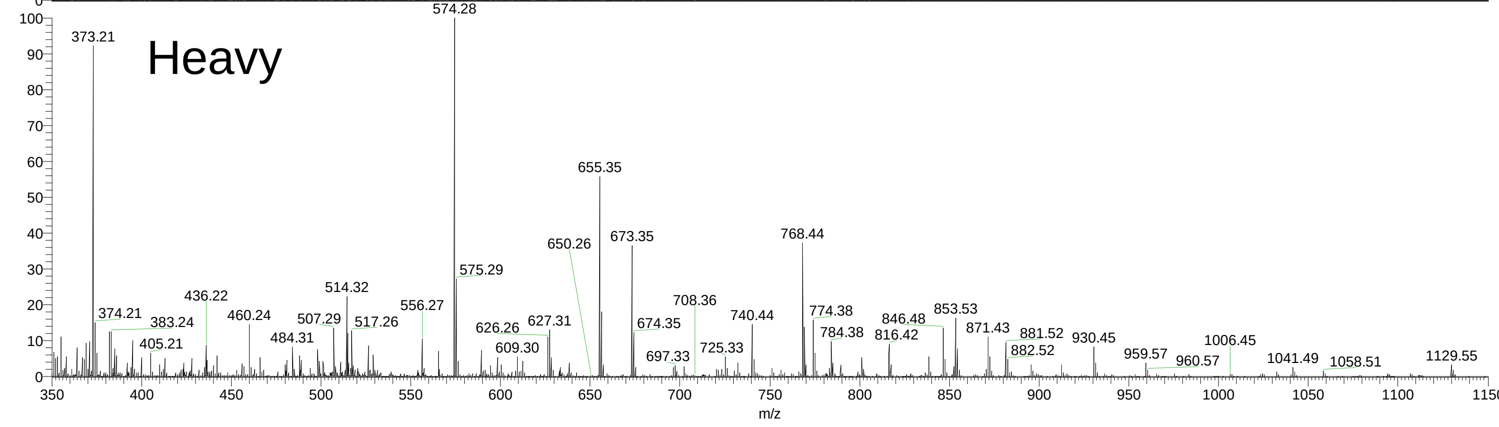

M; CTSB_80-87; LPASFDAR

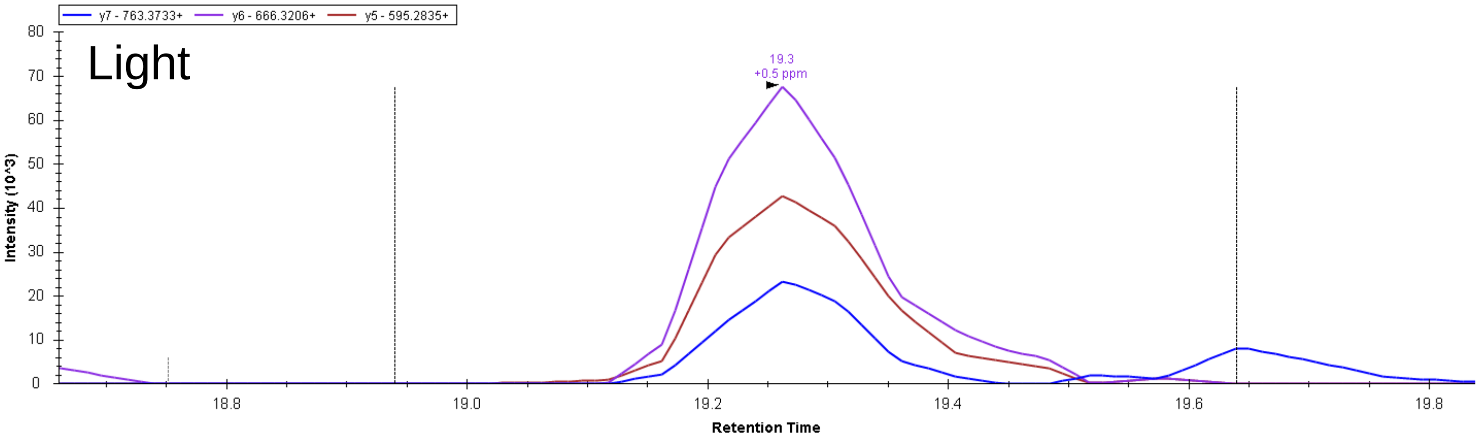

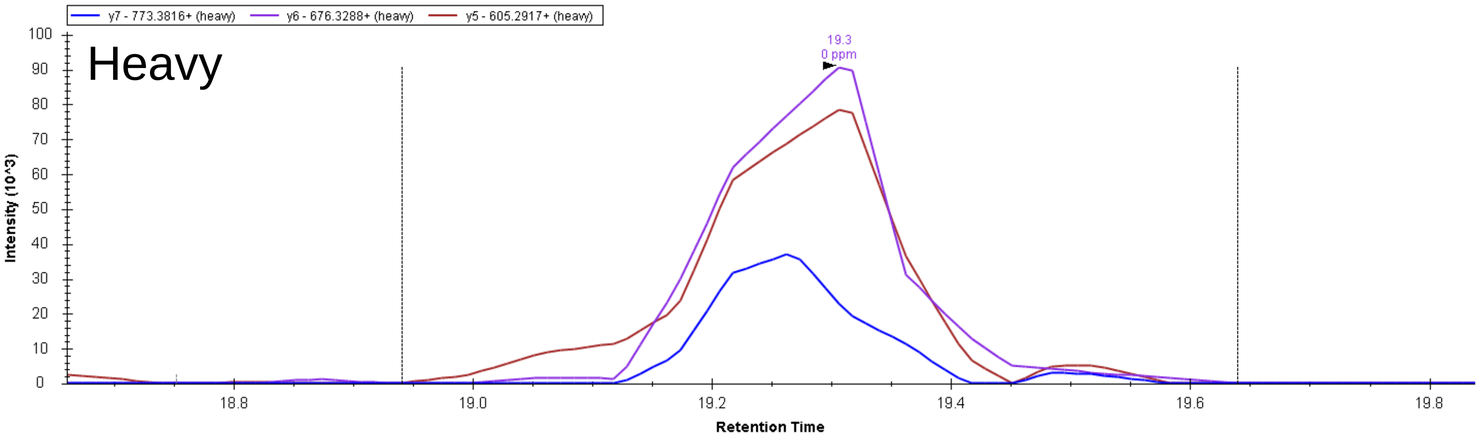

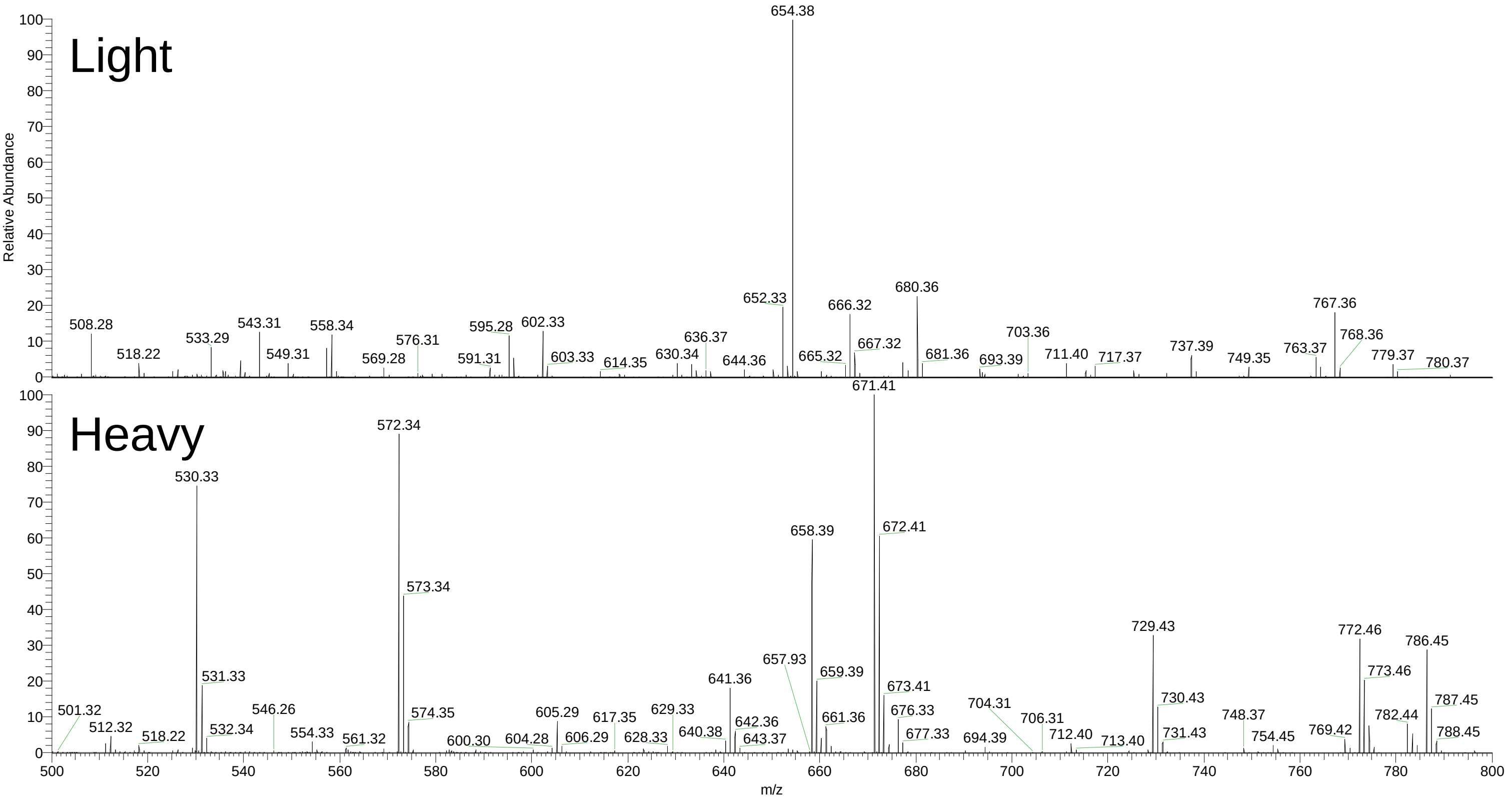

N; CTSB_210-220; IcEPGYSPTYK

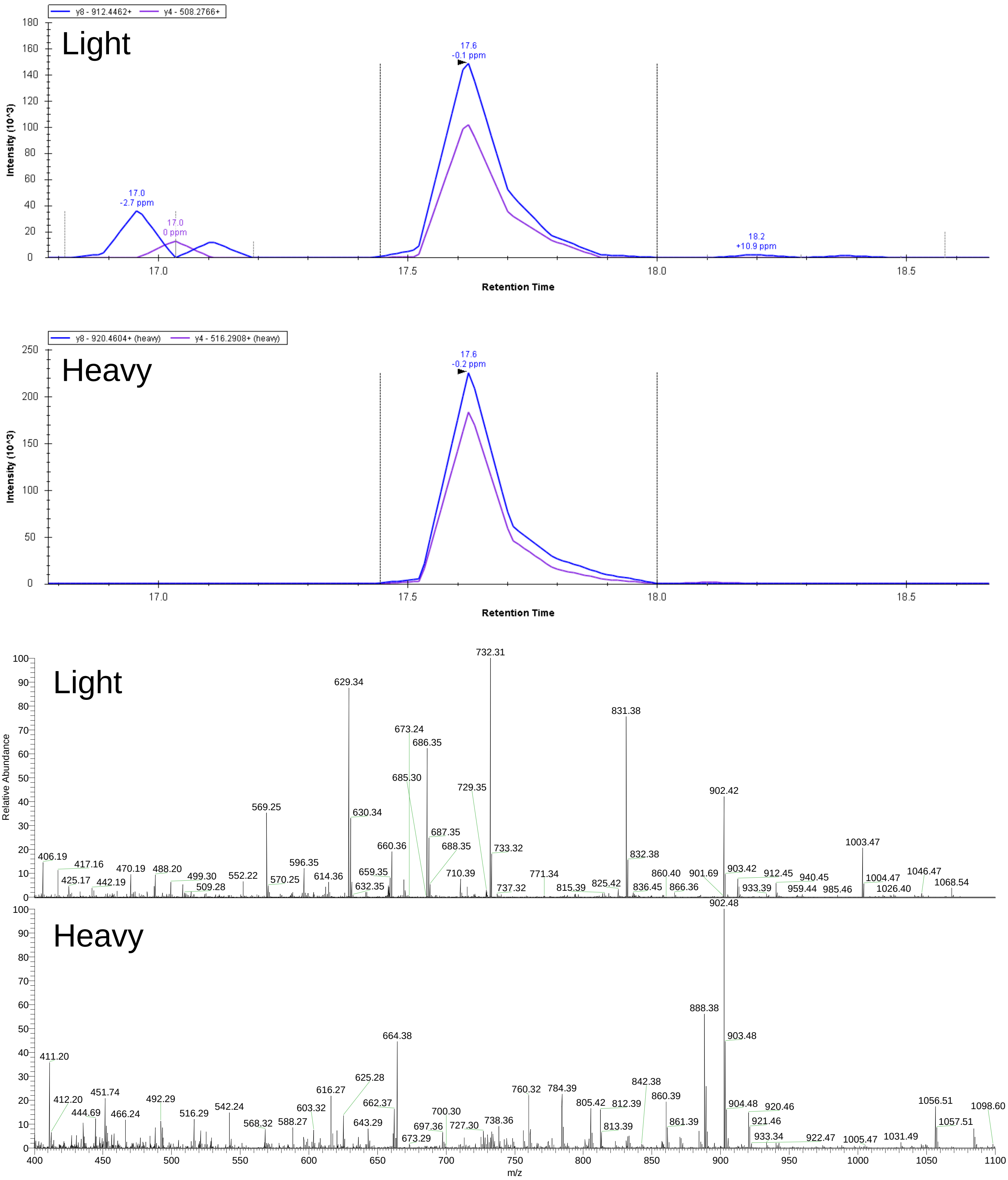

O; CTSD_55-72; YSQAVPAVTEGPIPEVLK

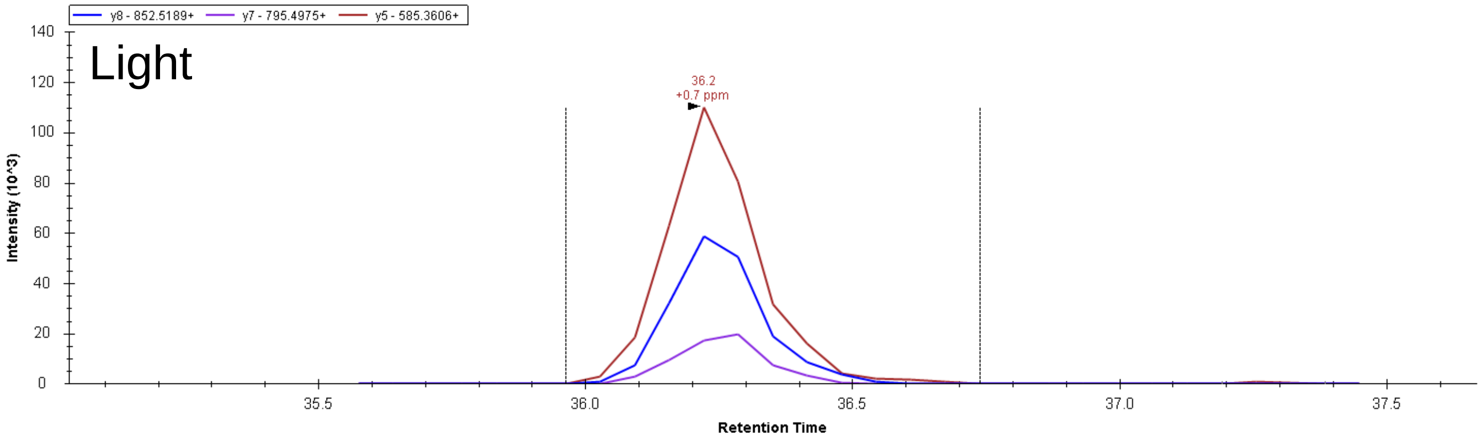

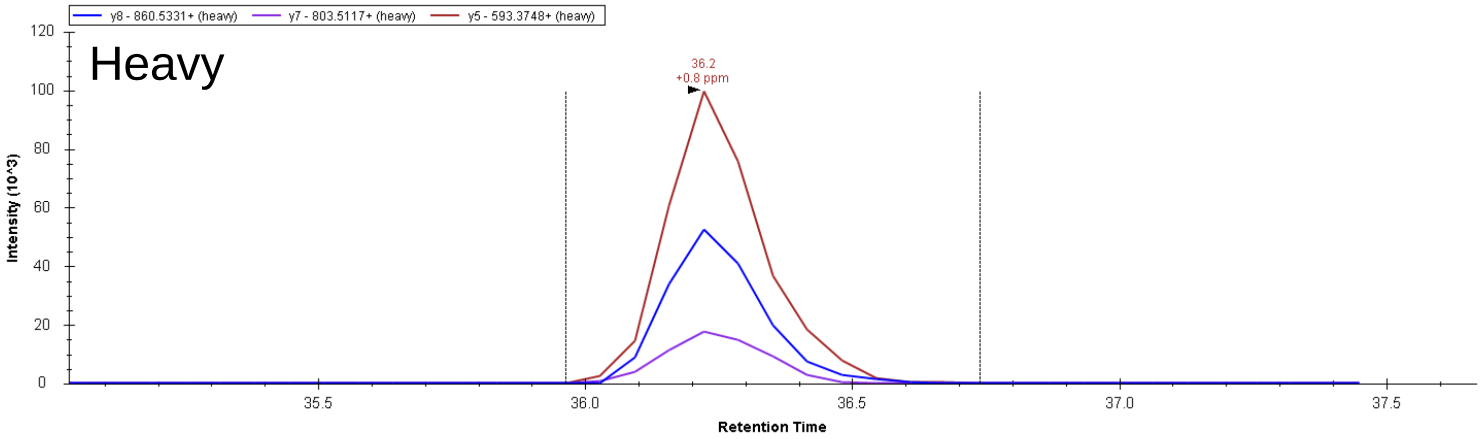

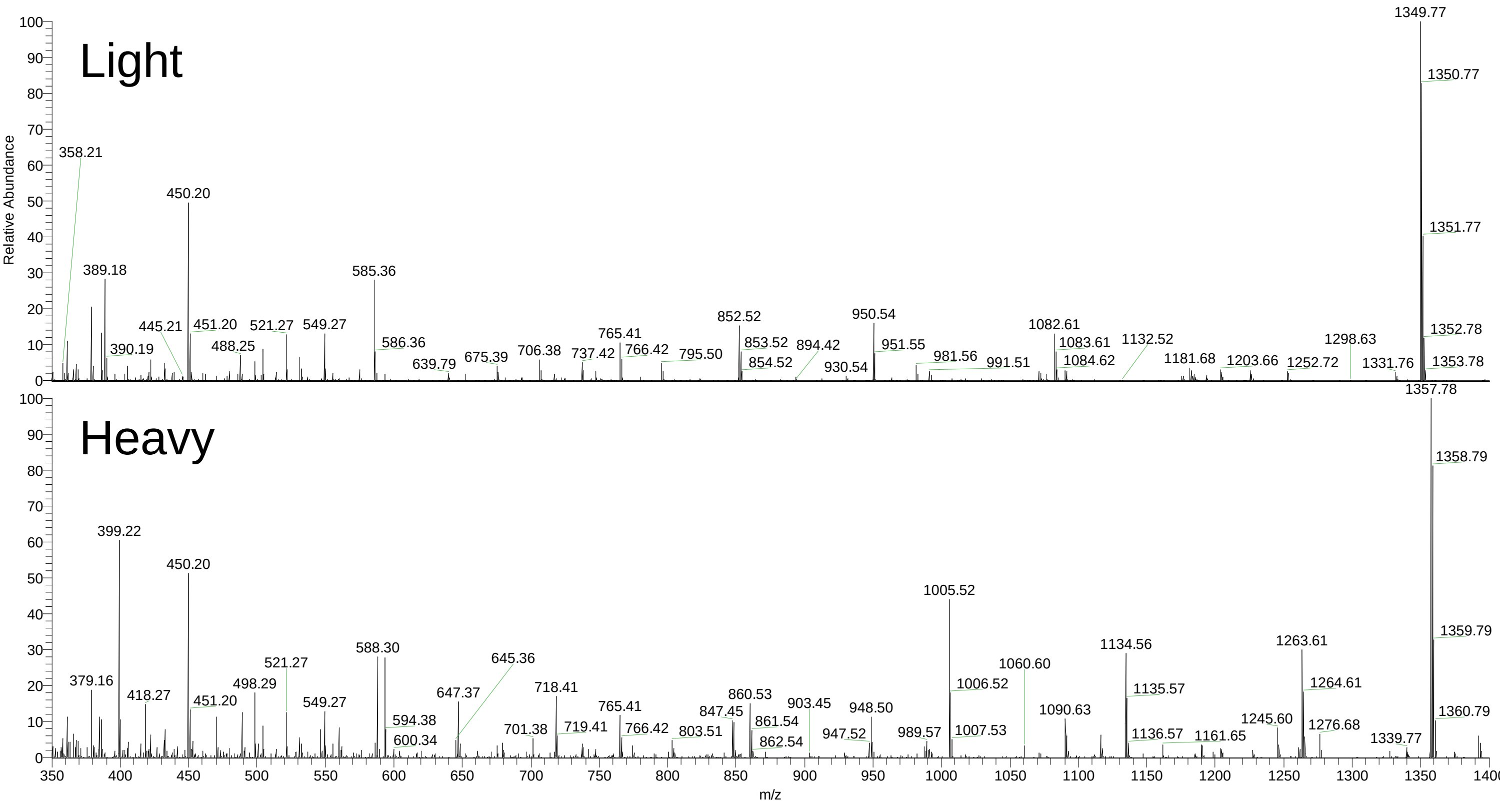

P; CTSD_112-122; LLDIAcWIHHK

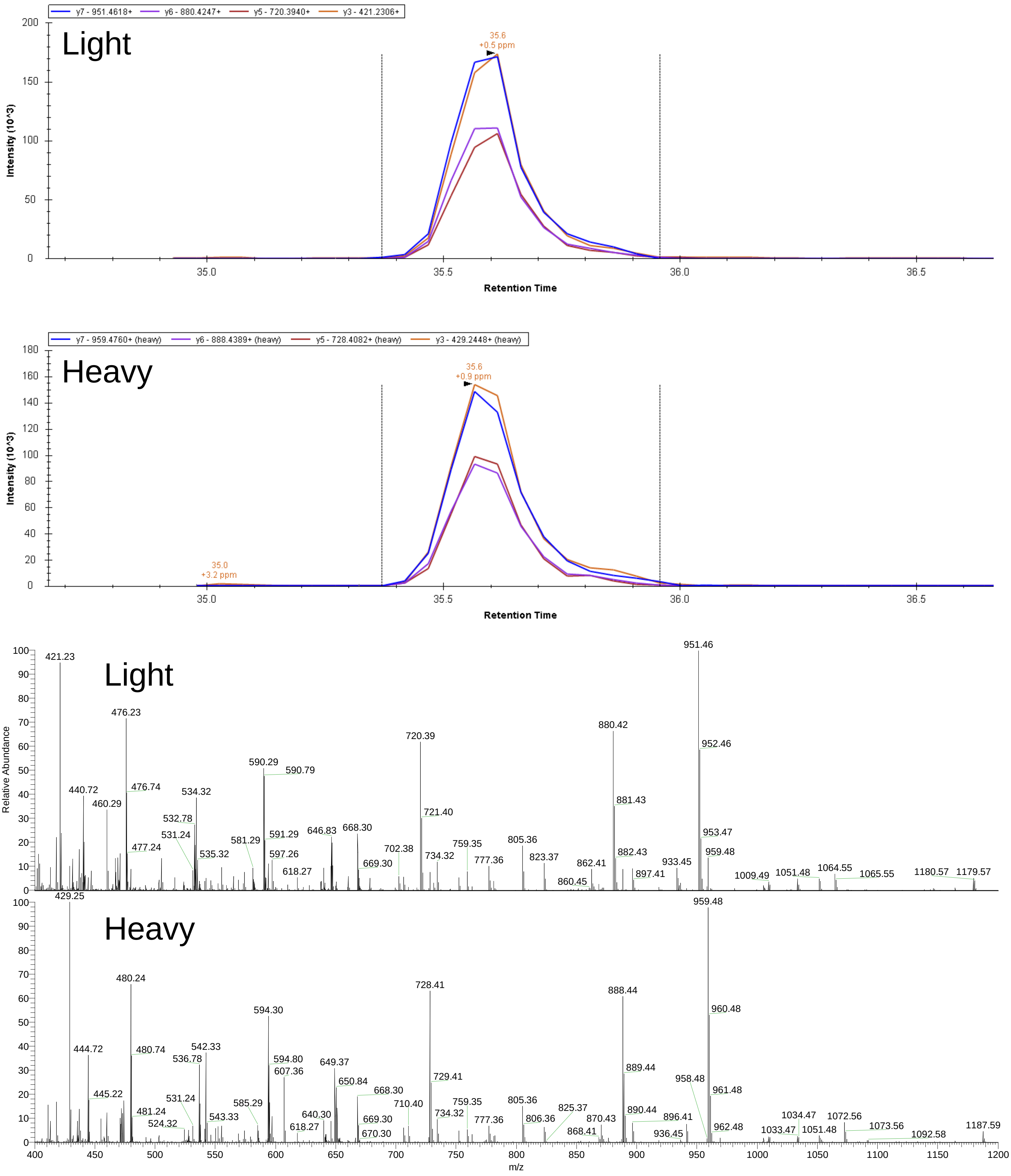

Q; CTSD_349-357; LSPEDYTLK

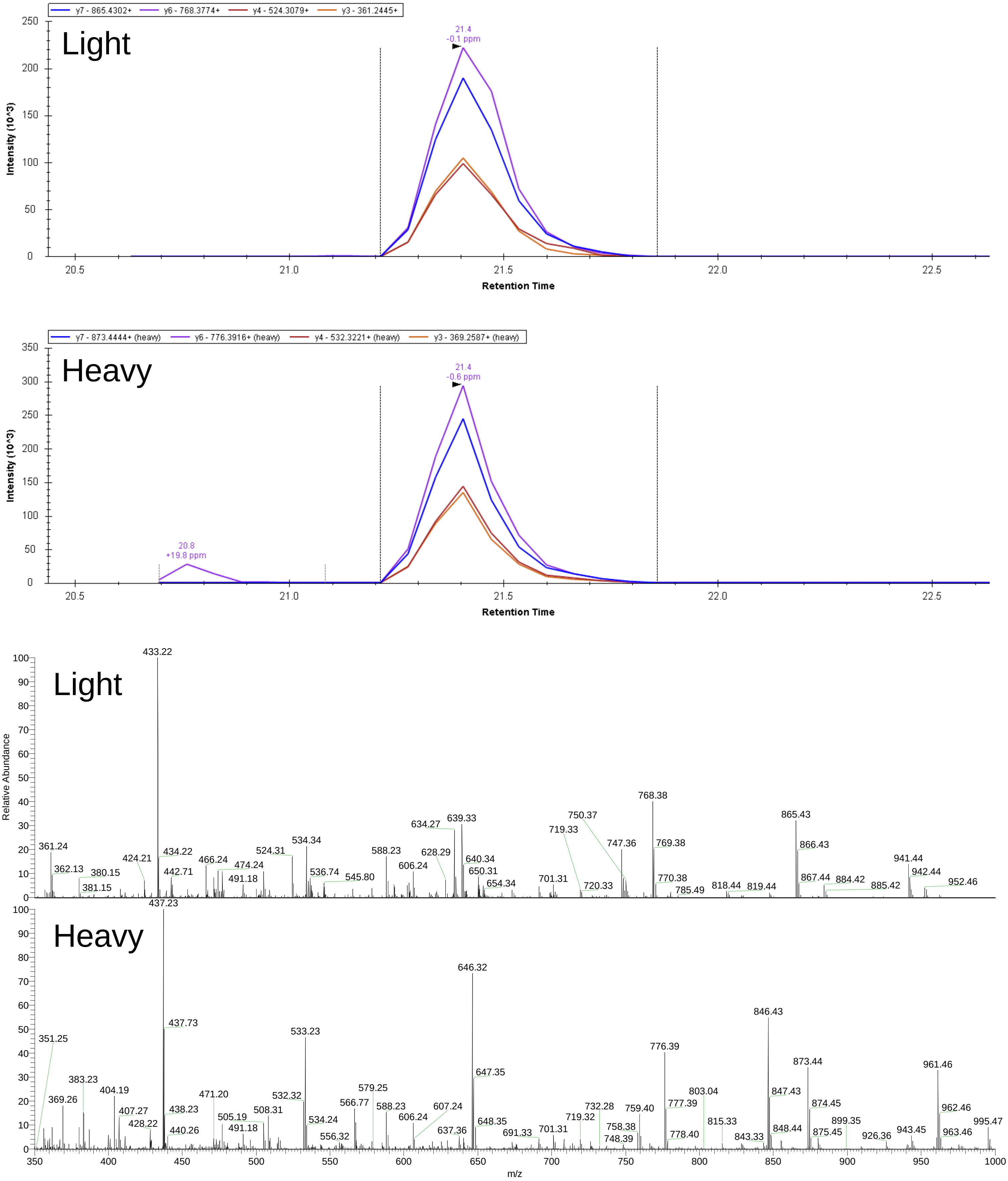

R; CTSF_103-116; TLLcSFQVLDELGR

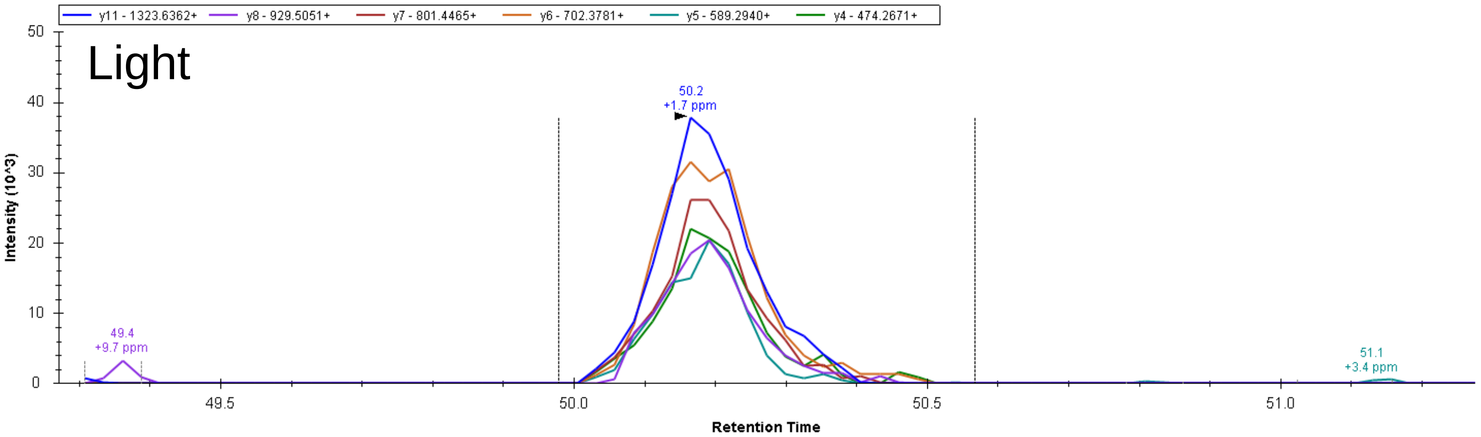

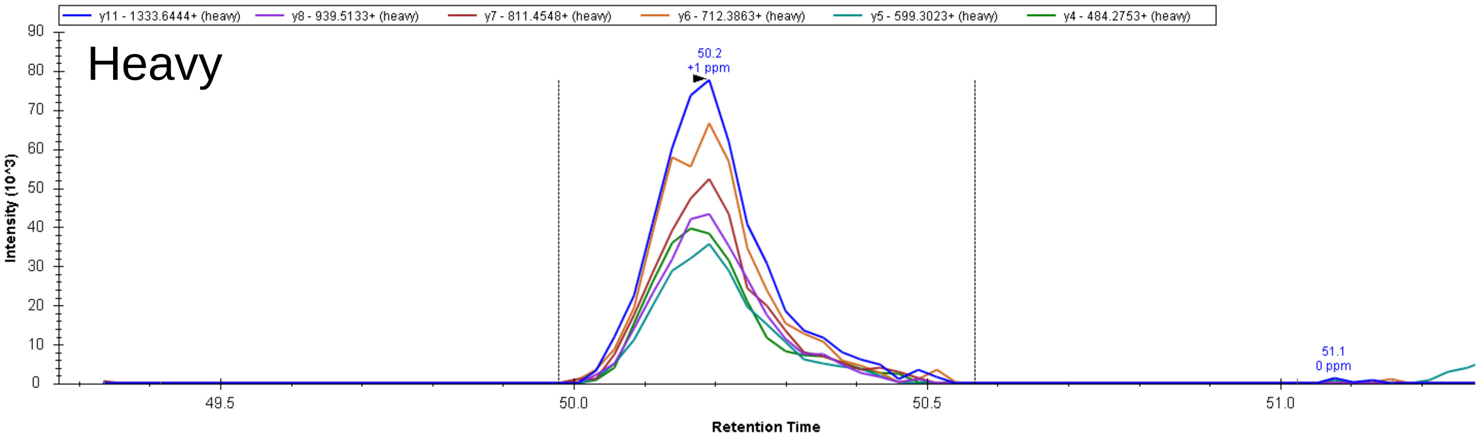

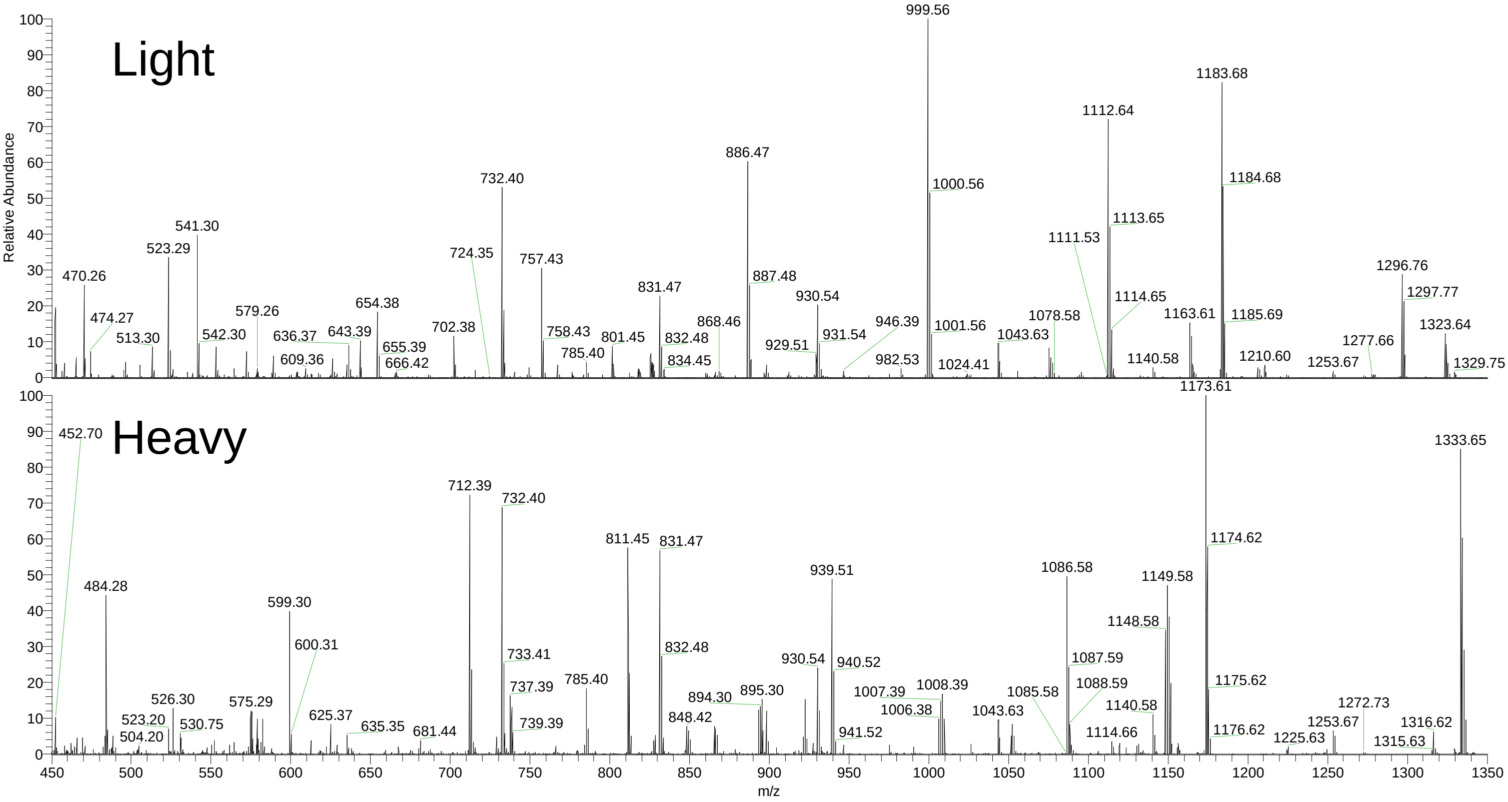

S; CTSF_236-245; FSDLTEEEFR

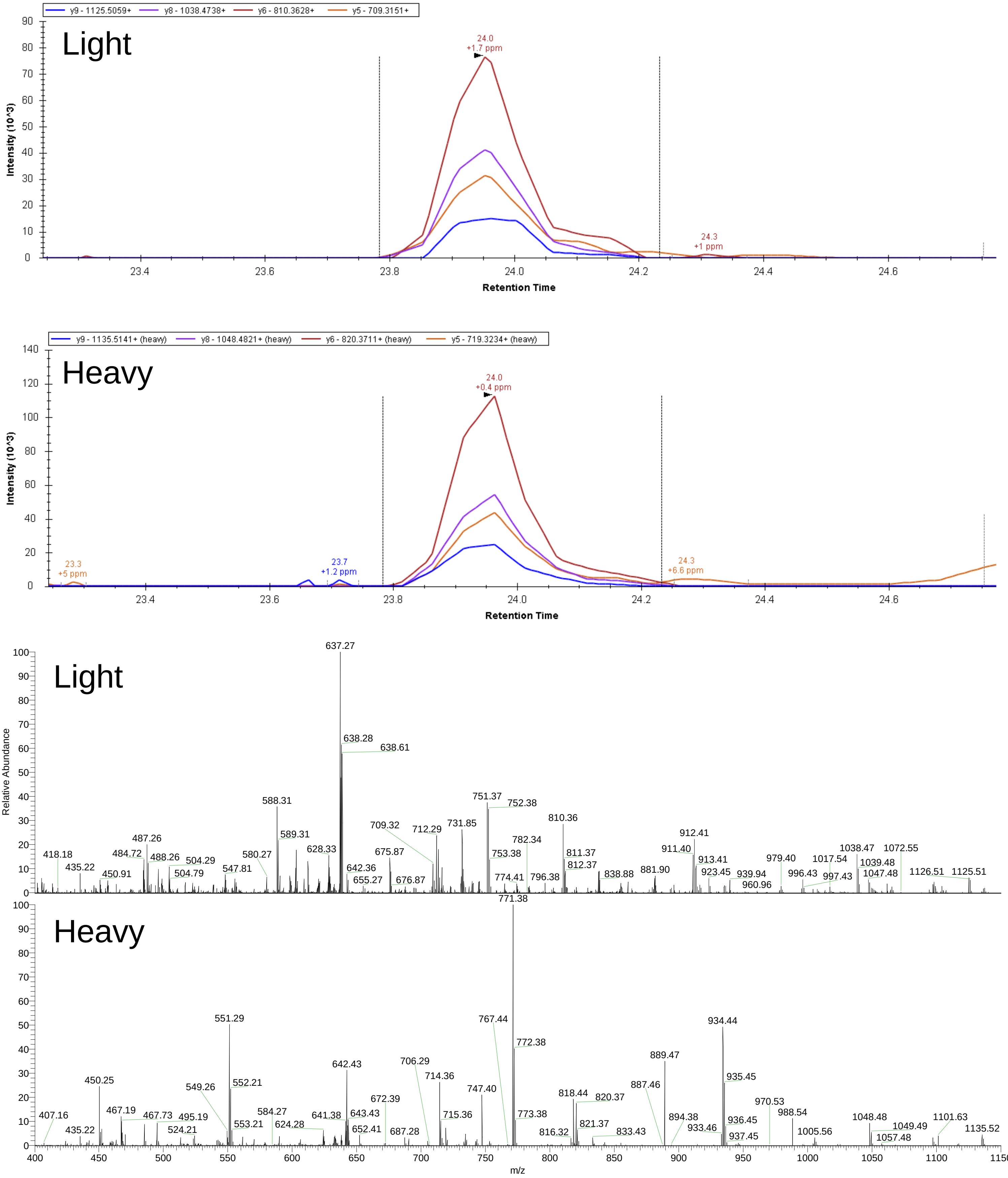

T; CTSF_266-278; SVGDLAPPEWDWR

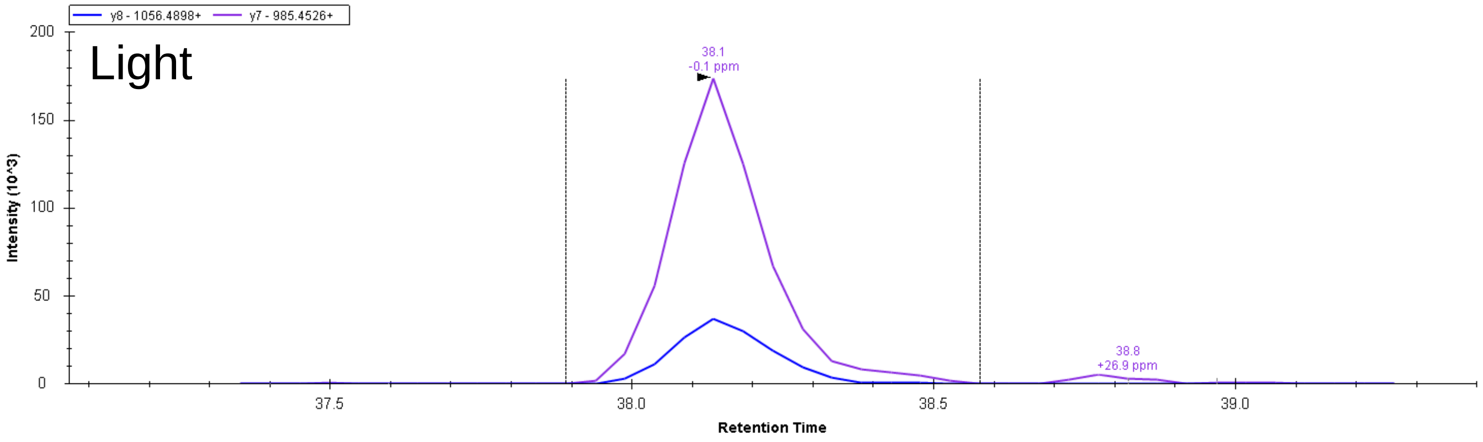

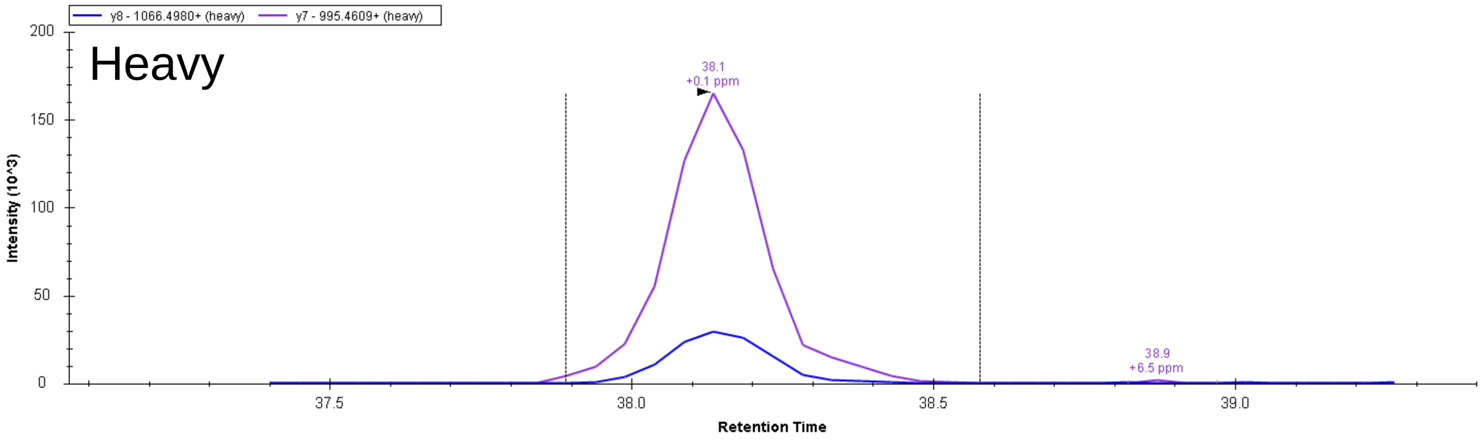

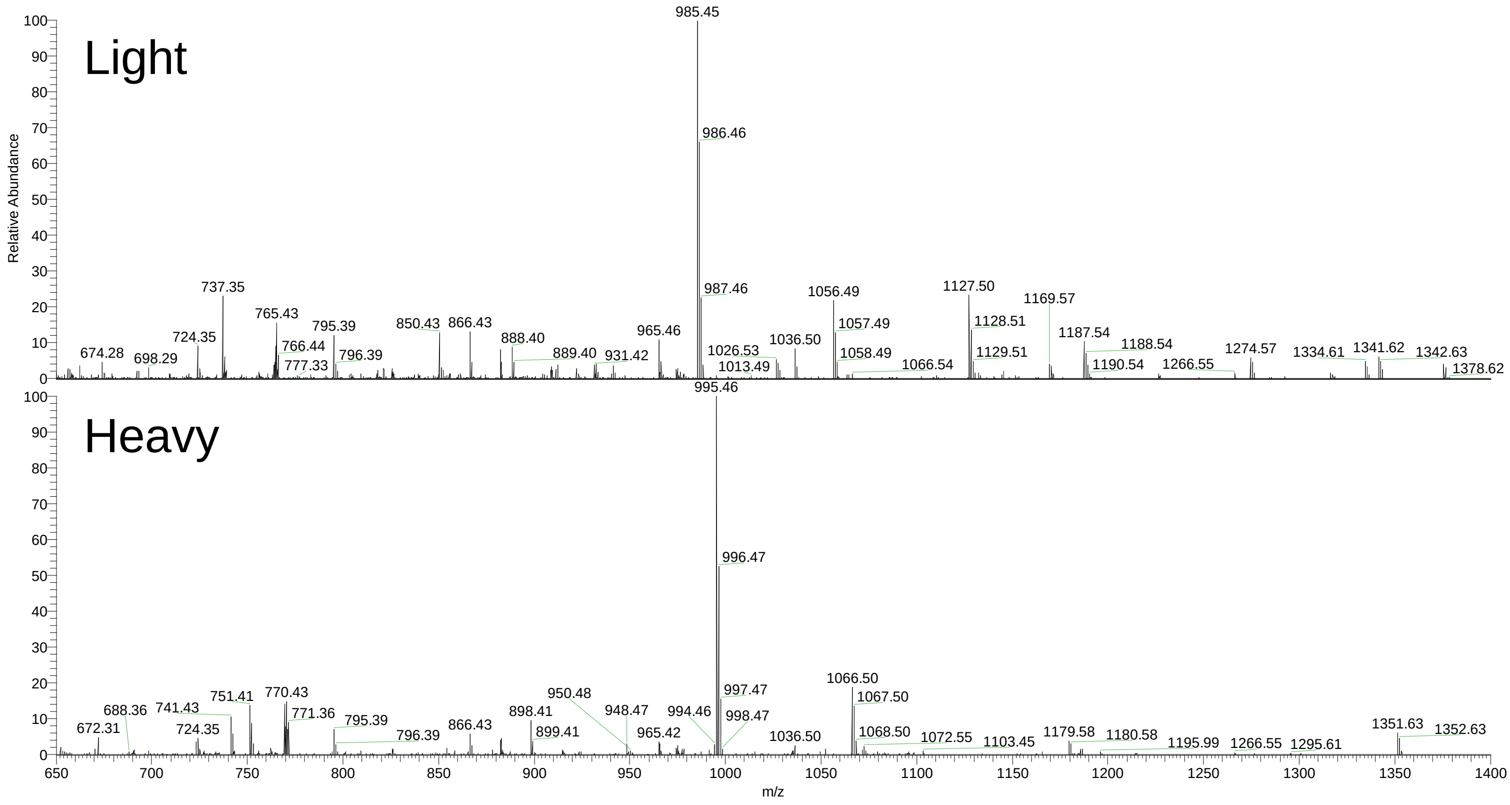

U; CTSF_442-450; SDVPFWAIK

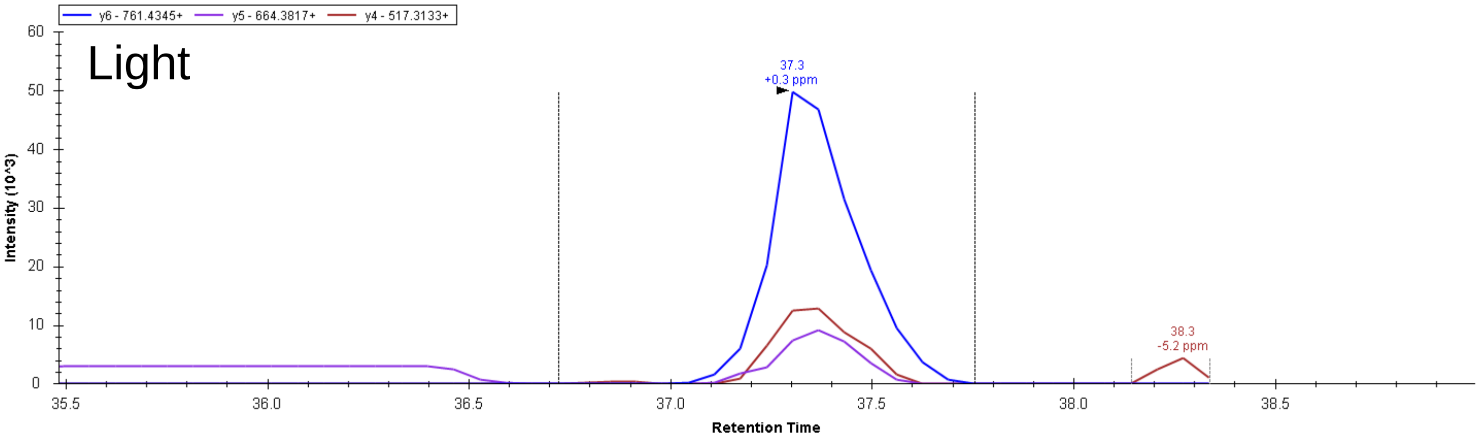

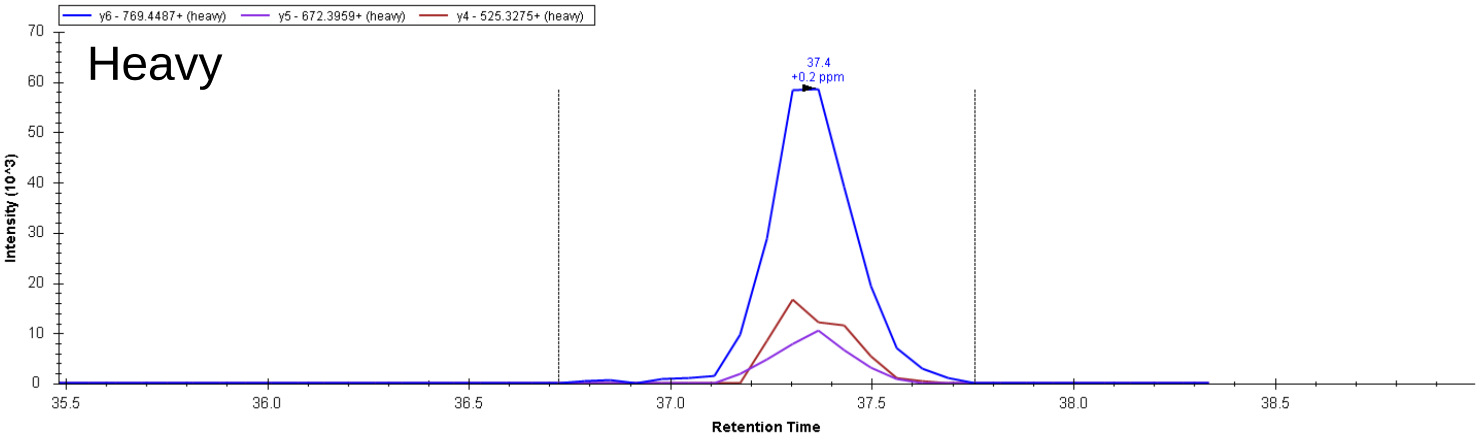

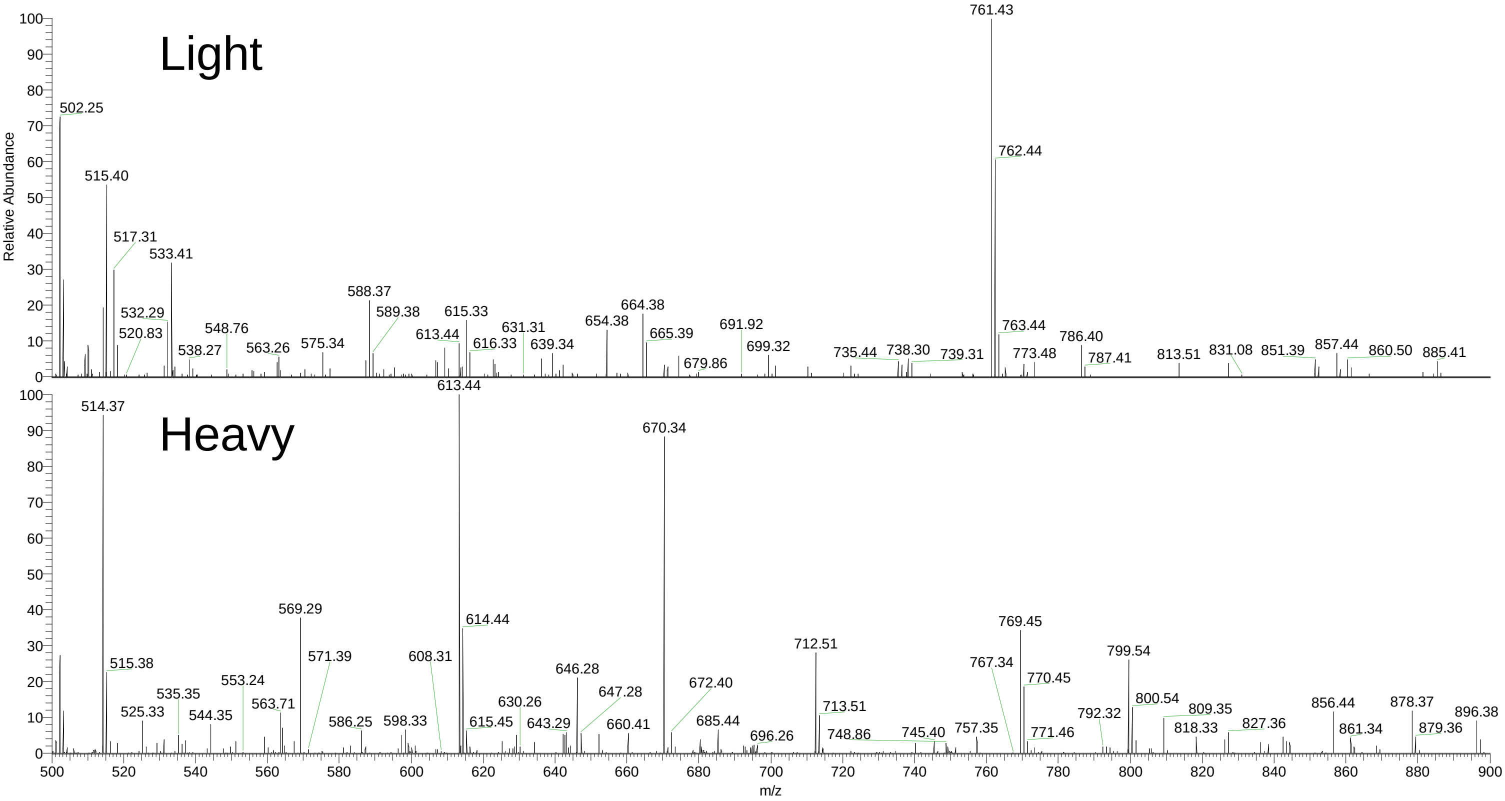

V; CTSL_105-116; VFQEPLFYEAPR

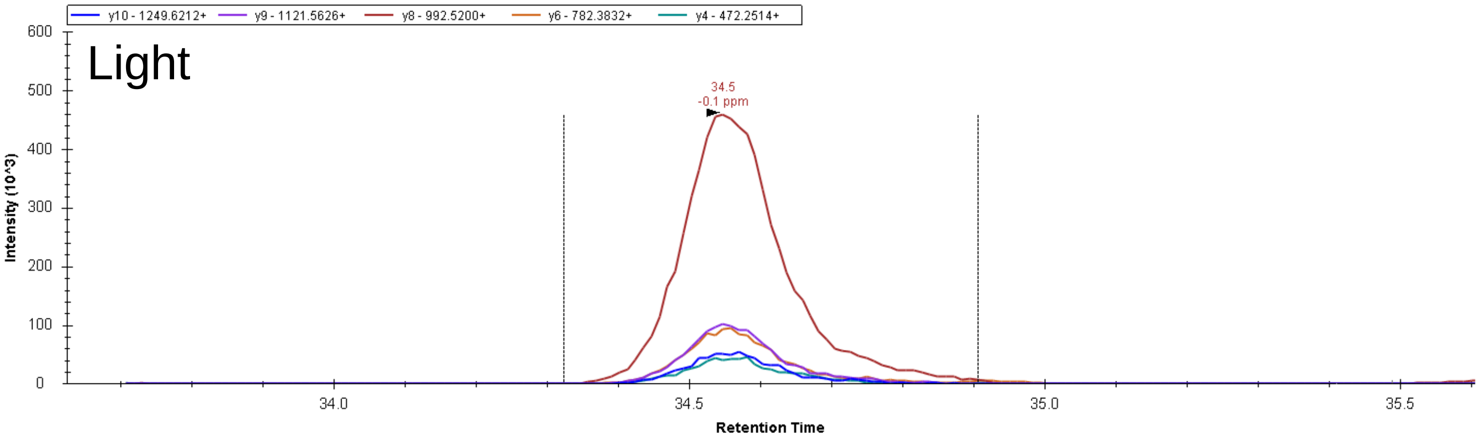

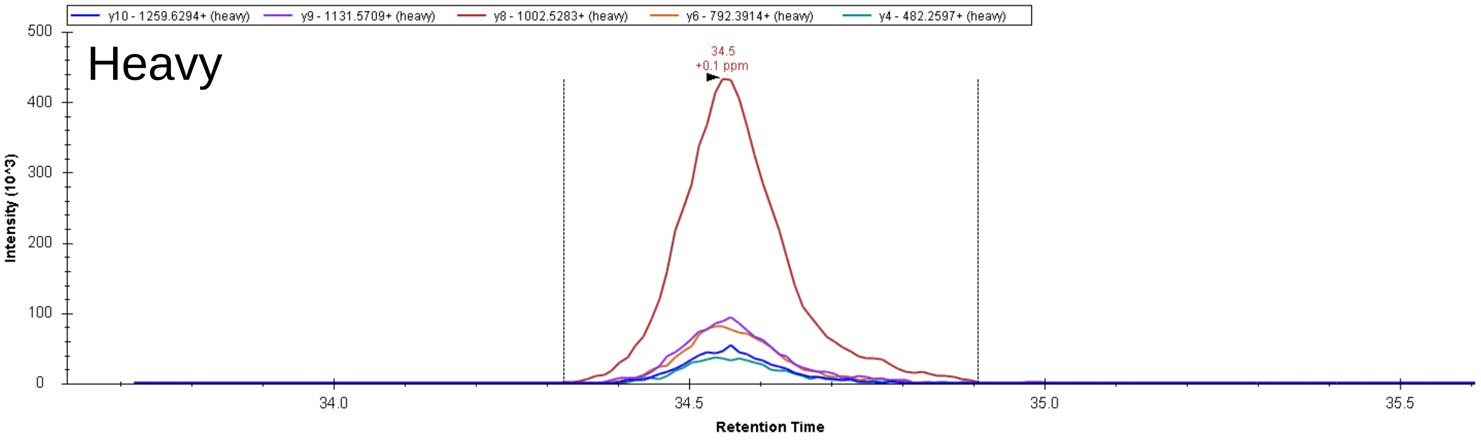

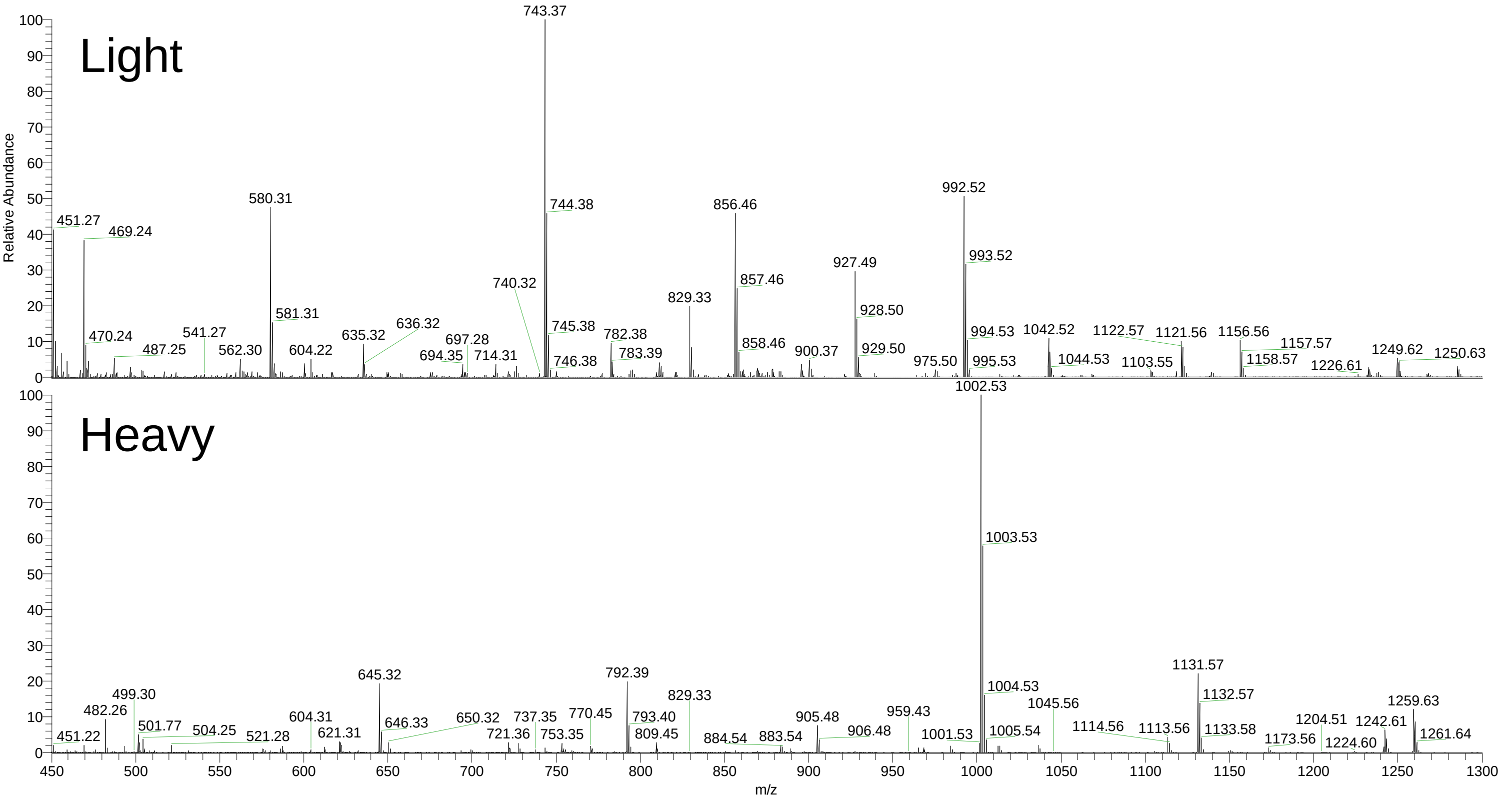

W; CTSZ_39-47; GDGLAPLGR

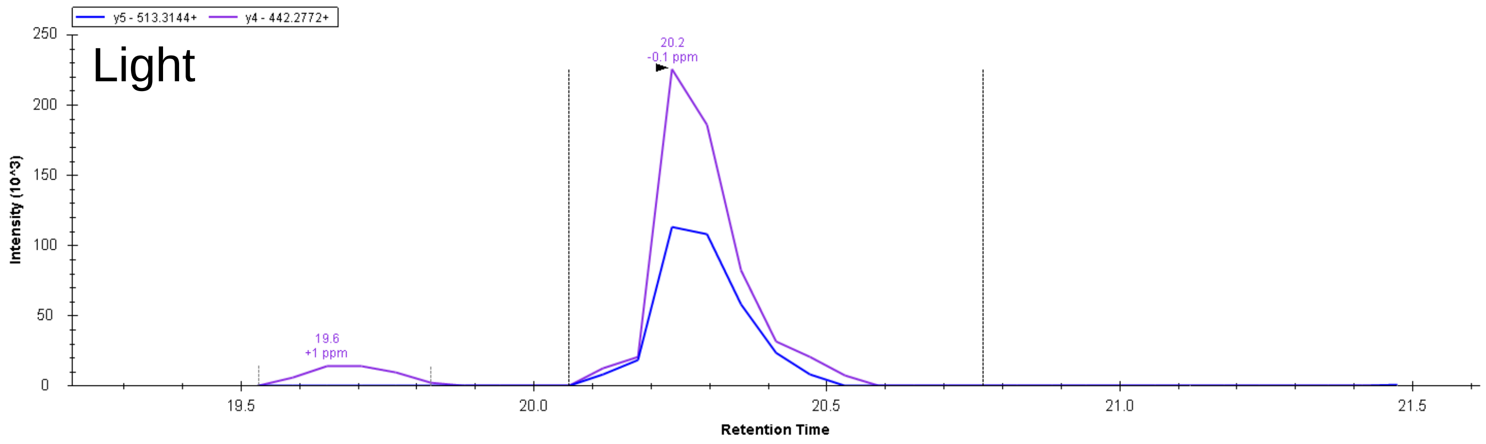

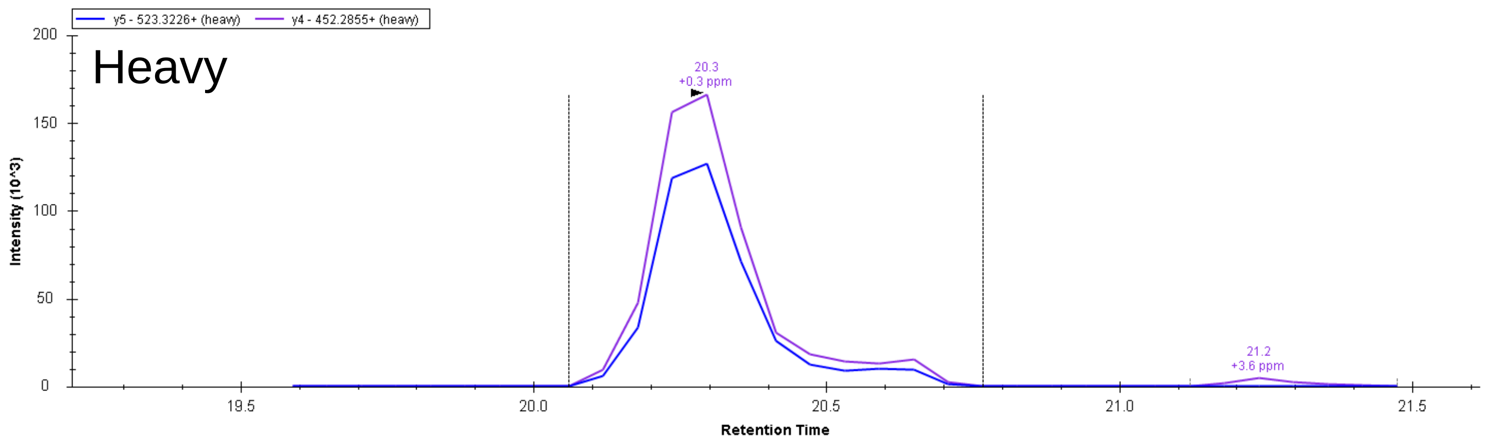

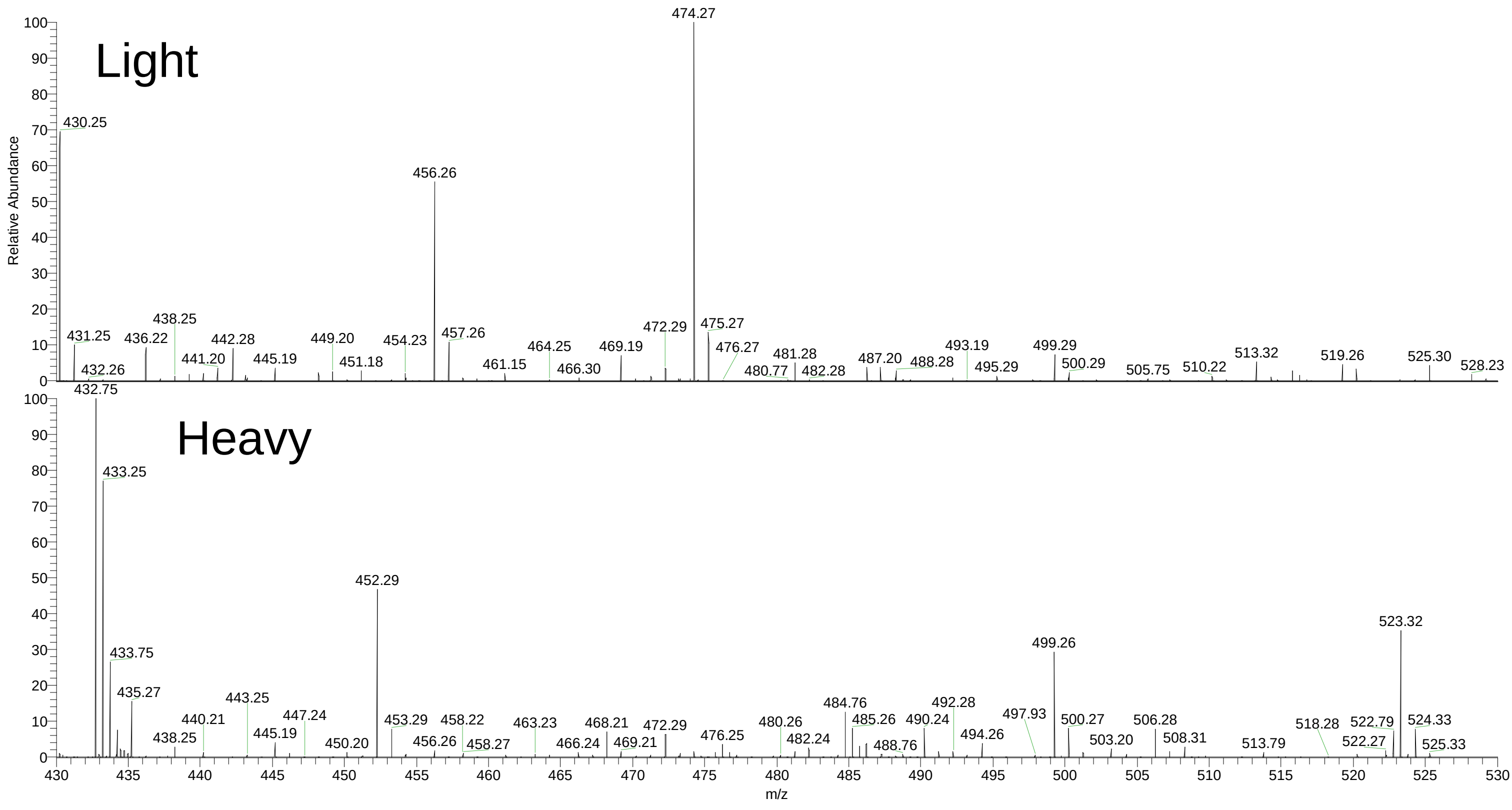

X; CTSZ_261-270; NSWGEPWGER

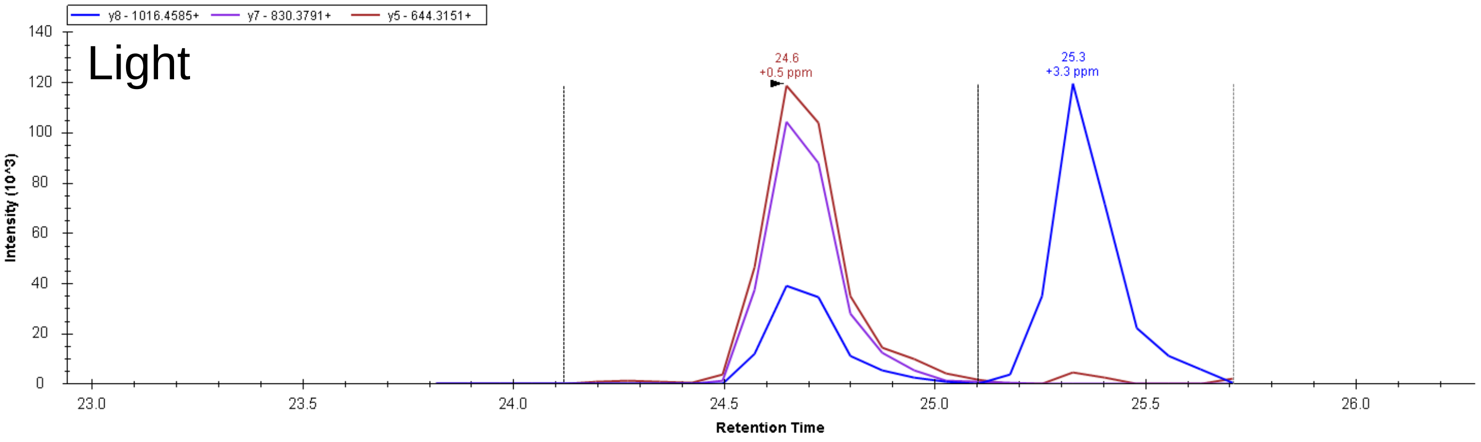

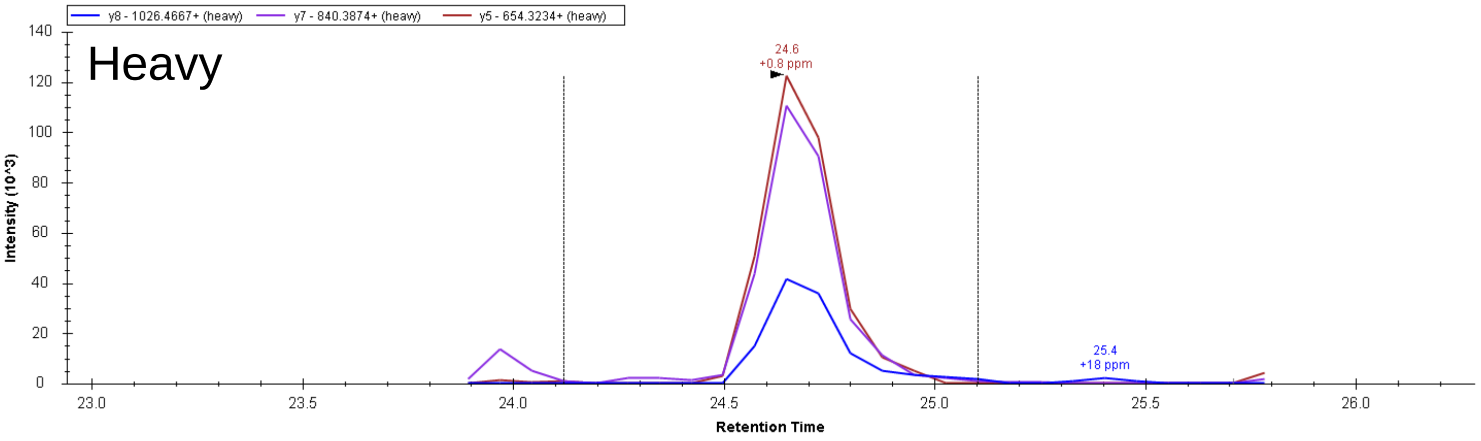

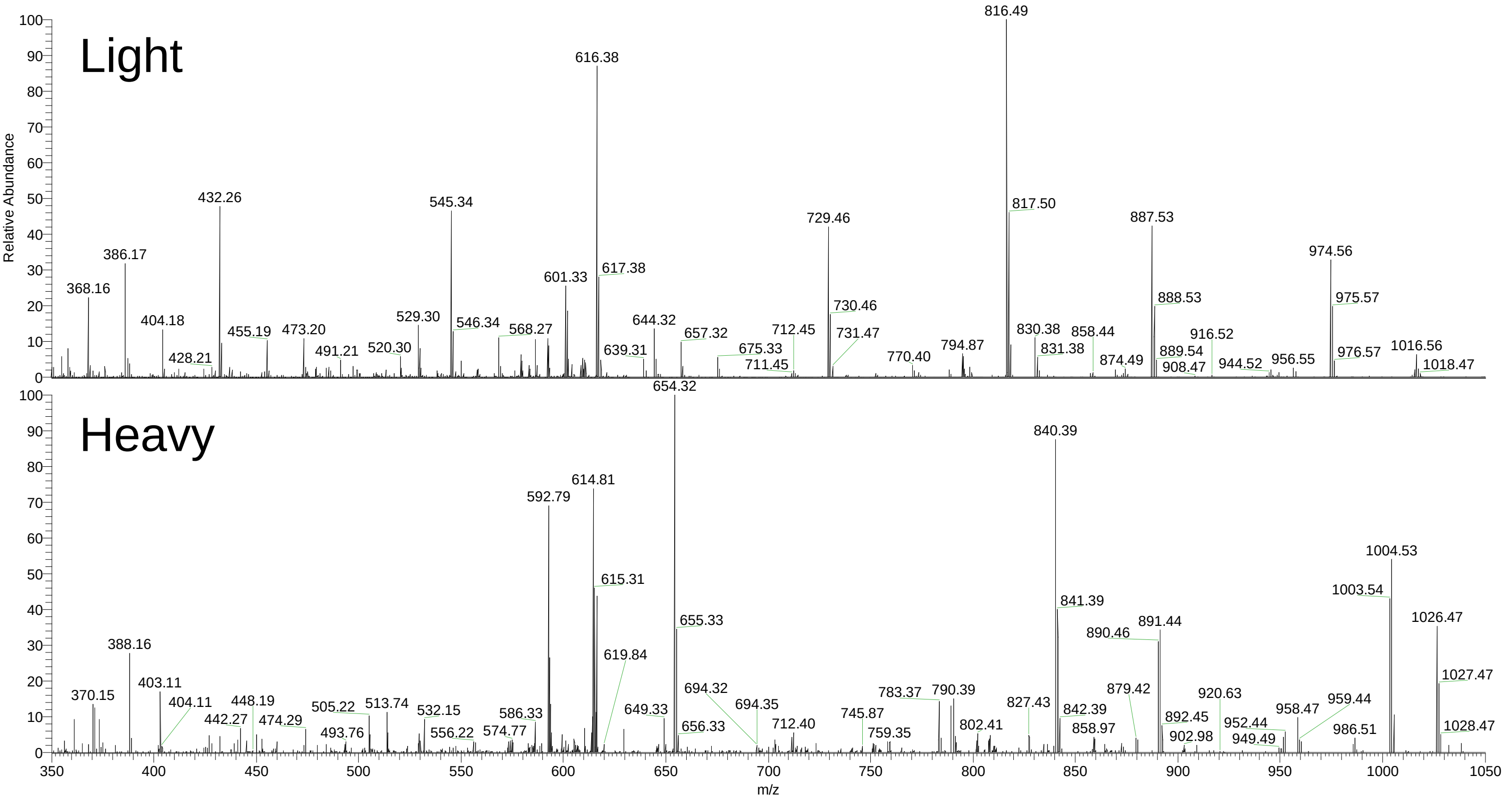

Y; DPP7_40-47, LDHFNFER

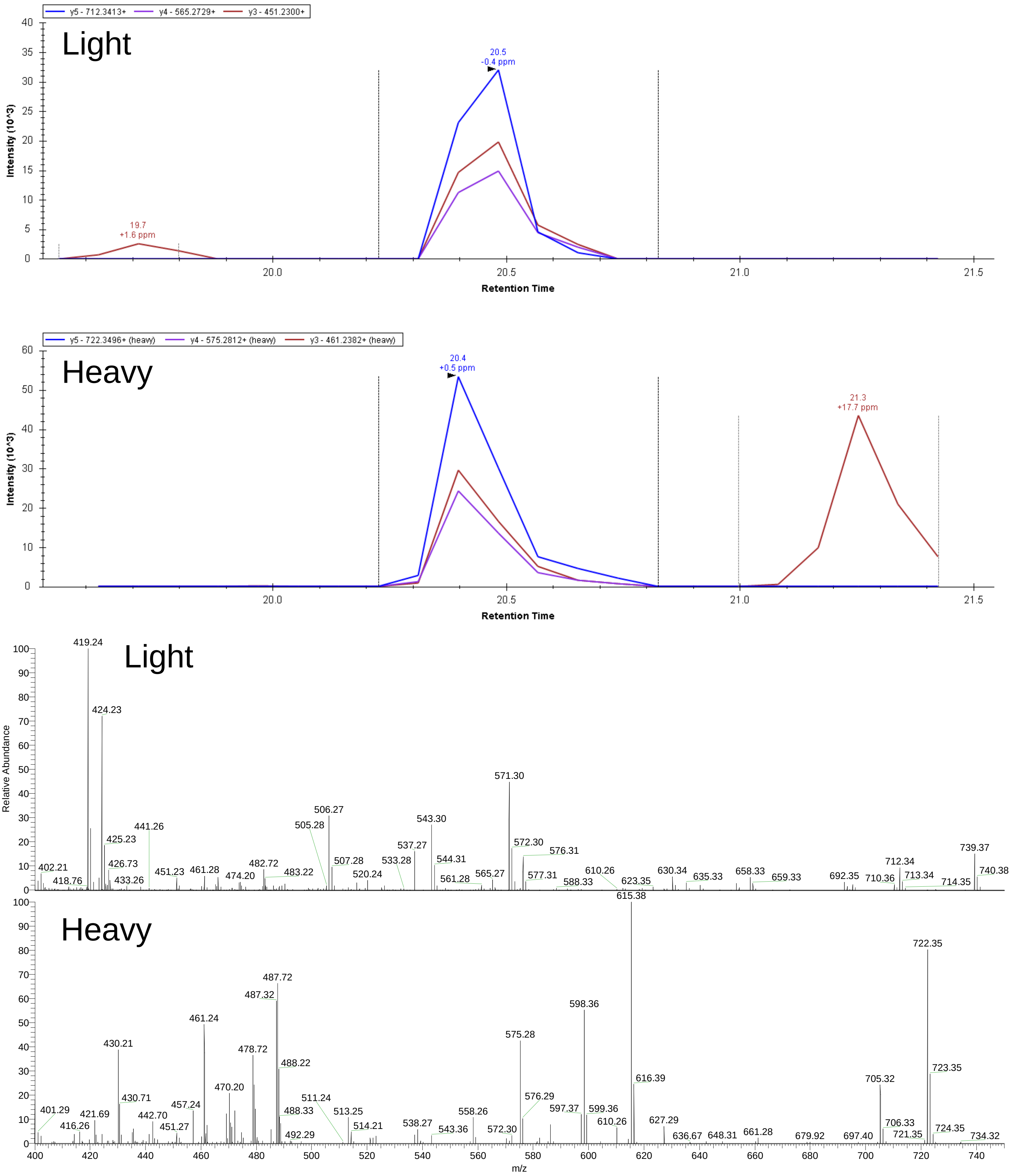

Z; DPP7_113-123; SLPFGAQSTQR

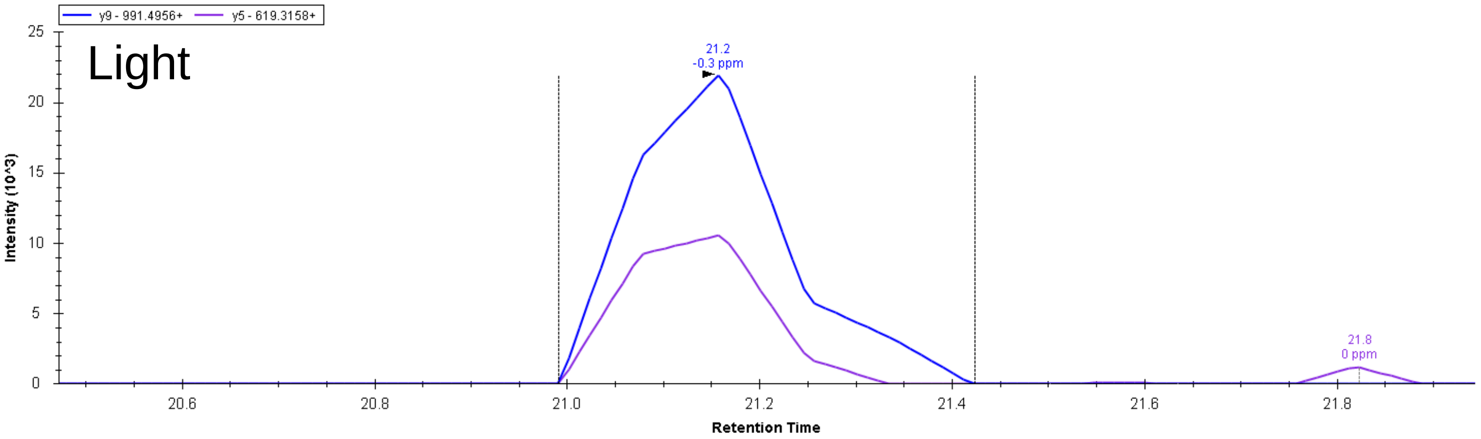

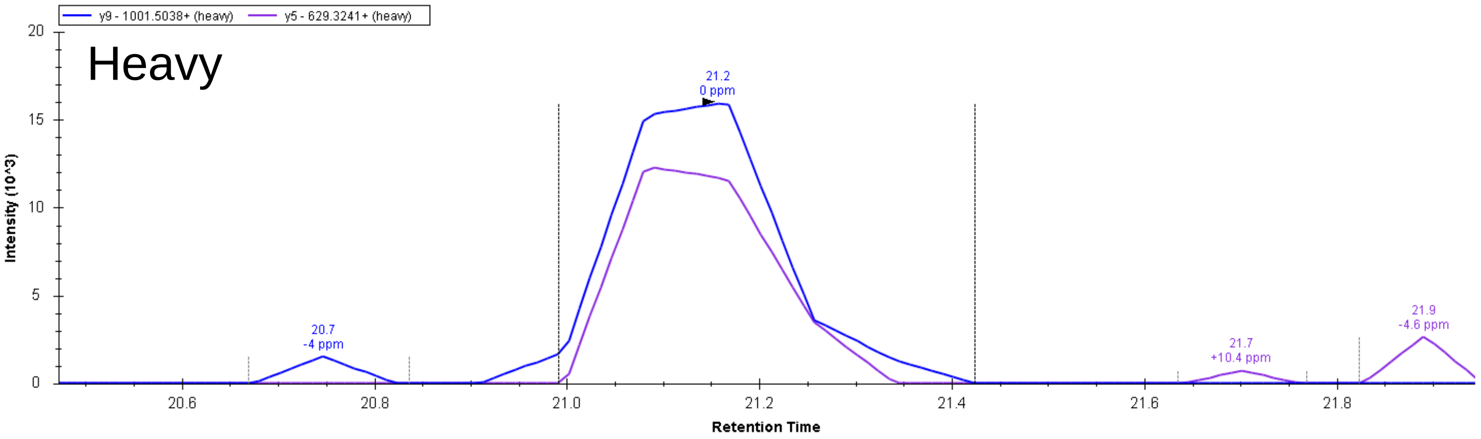

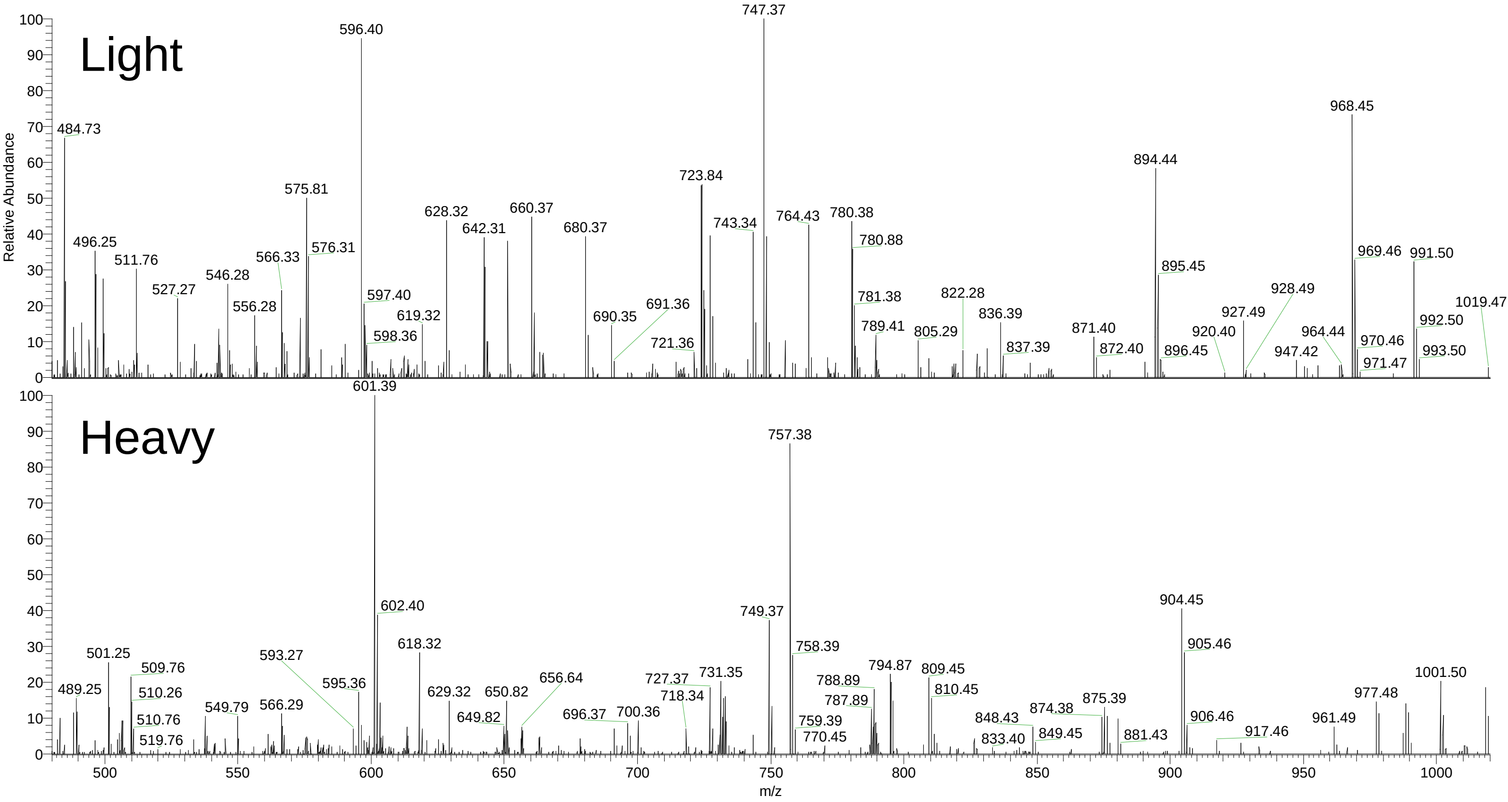

Aa; DPP7_449-462; ASHPEDPASVVEAR

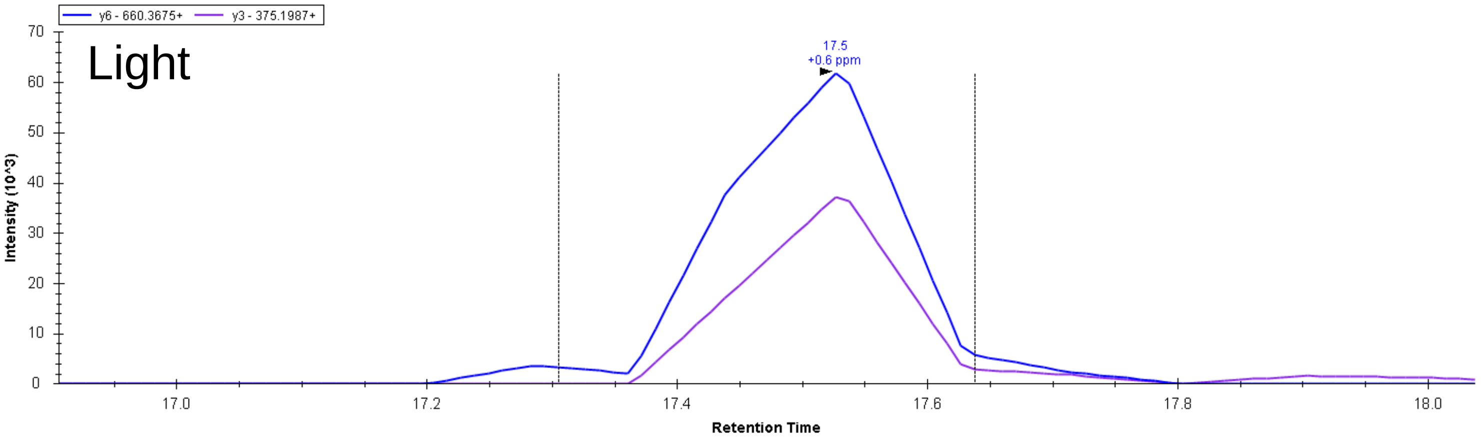

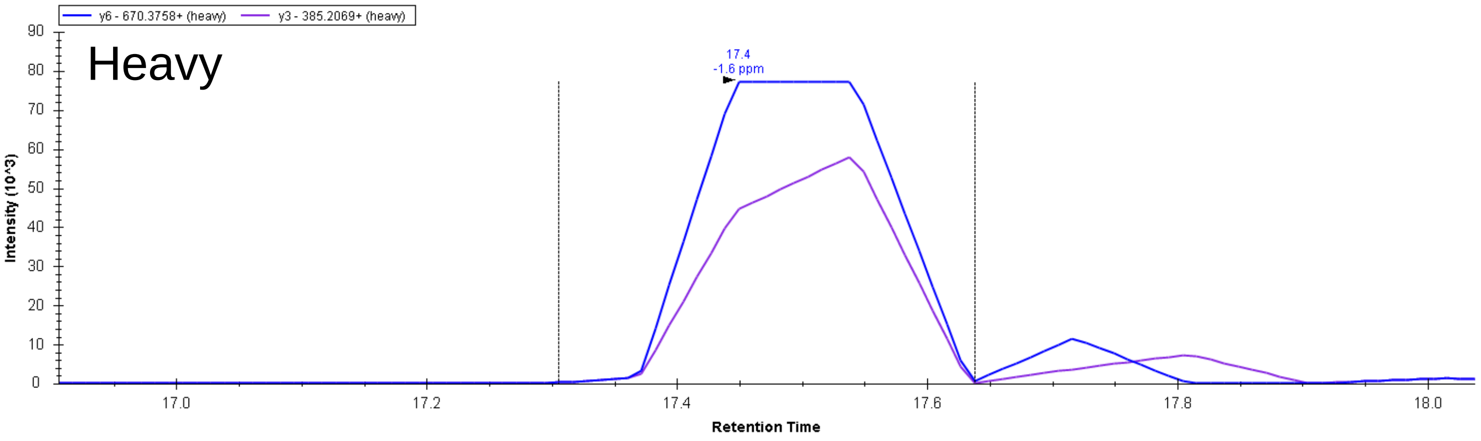

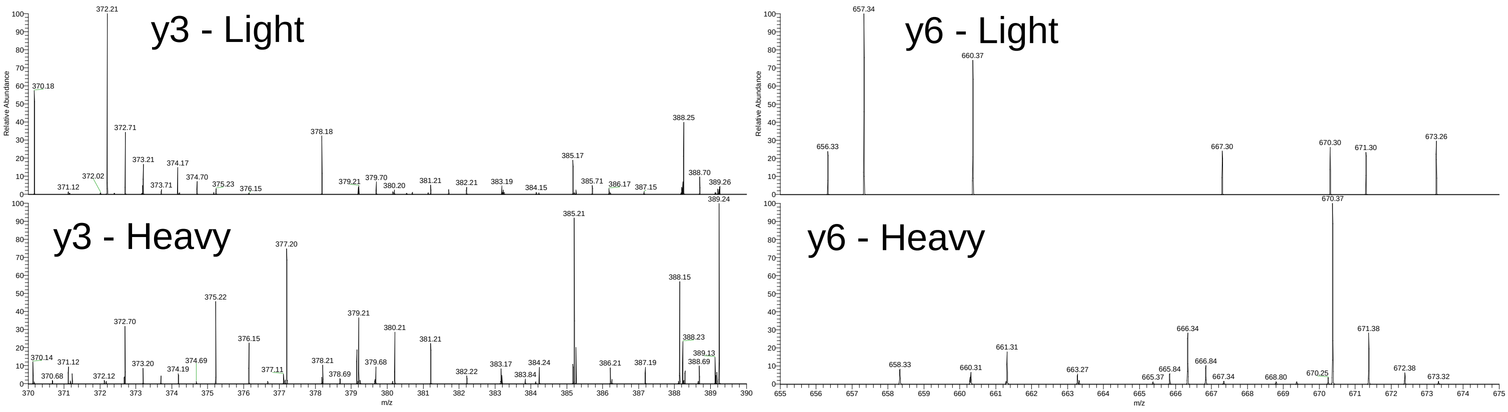

Ab; GM2A_89-96; EVAGLWIK

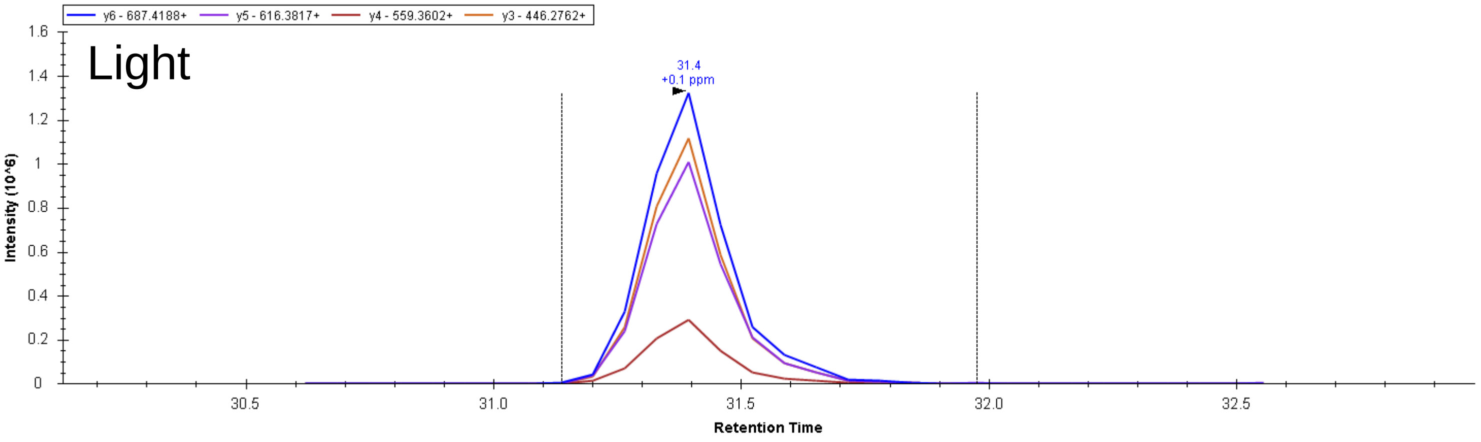

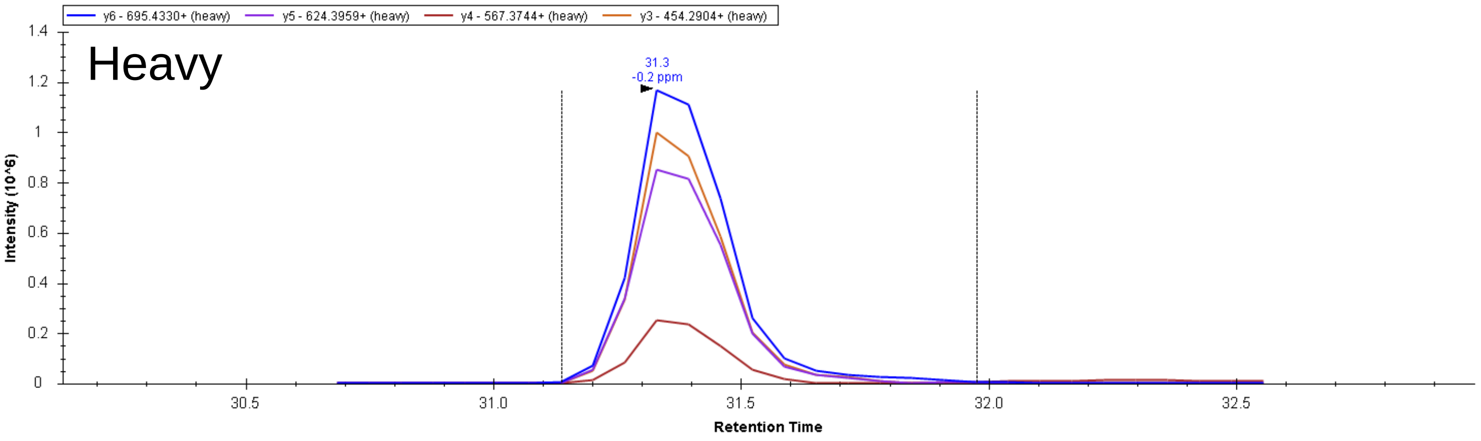

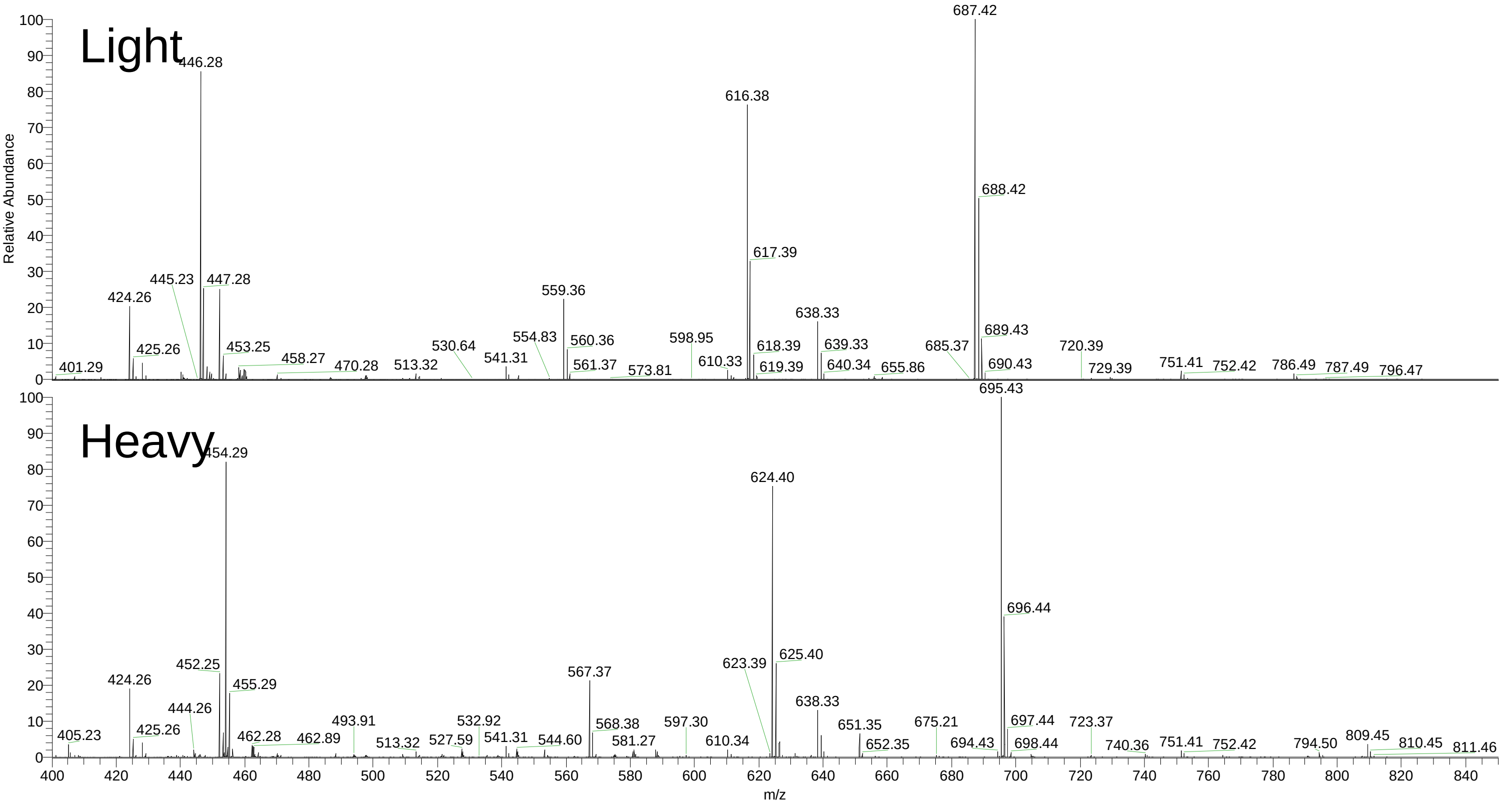

Ac; GM2A_170-179; IESVLSSSGK

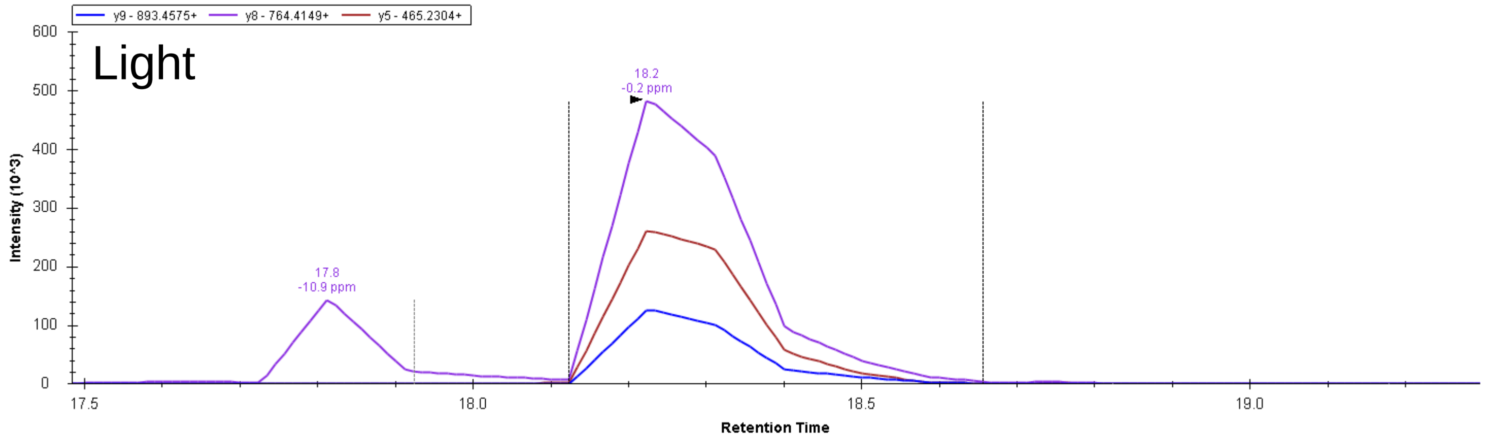

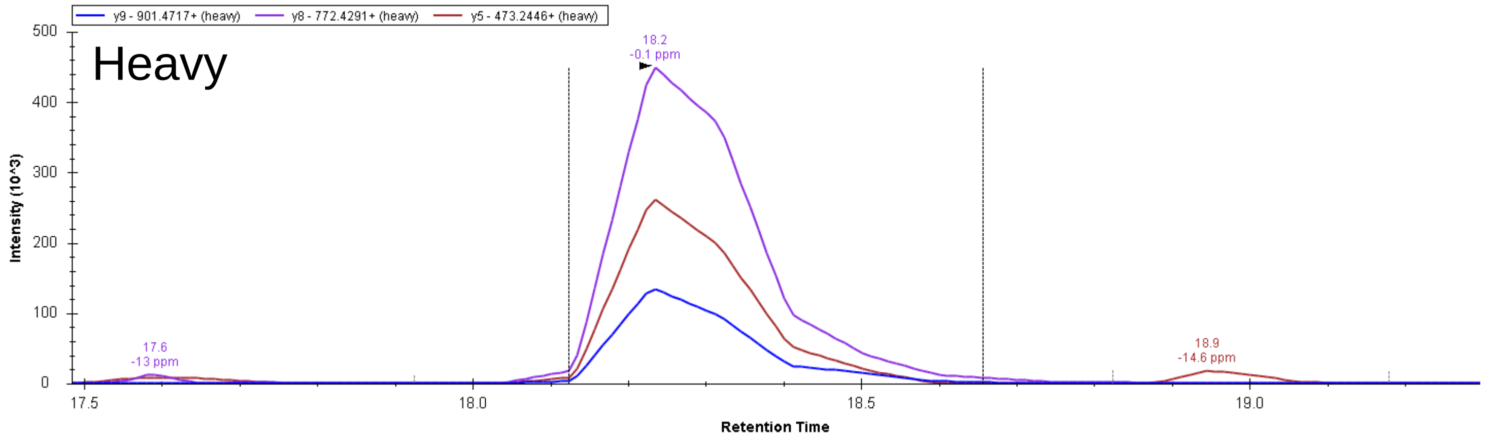

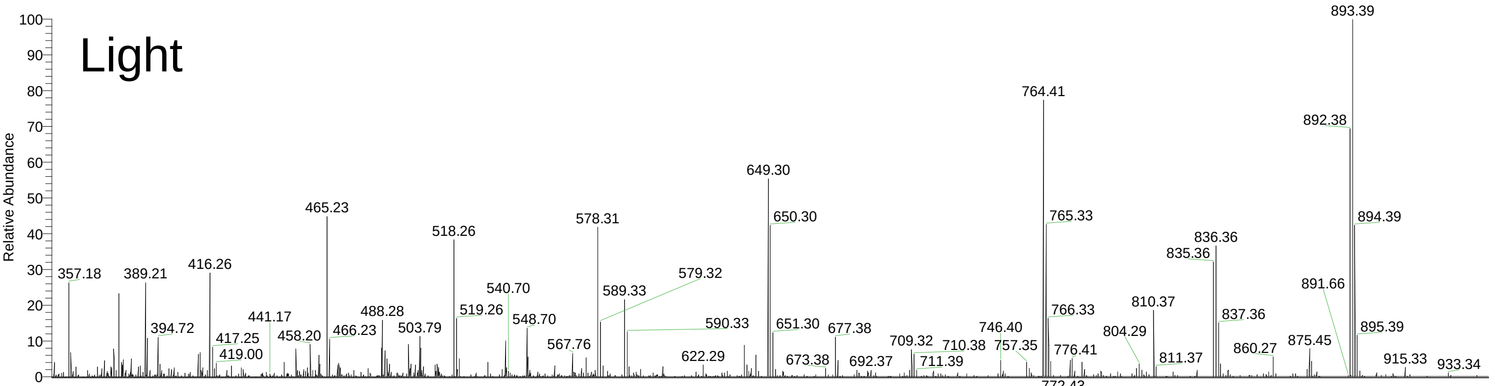

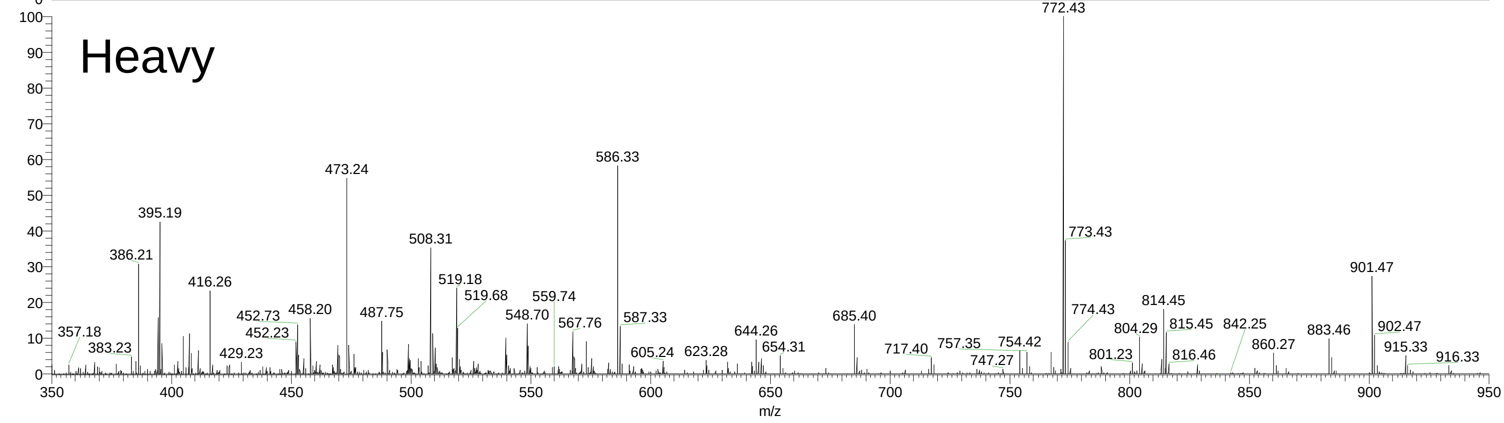

Ad; HEXB_285-300; VLPEFDTPGHTLSWGK

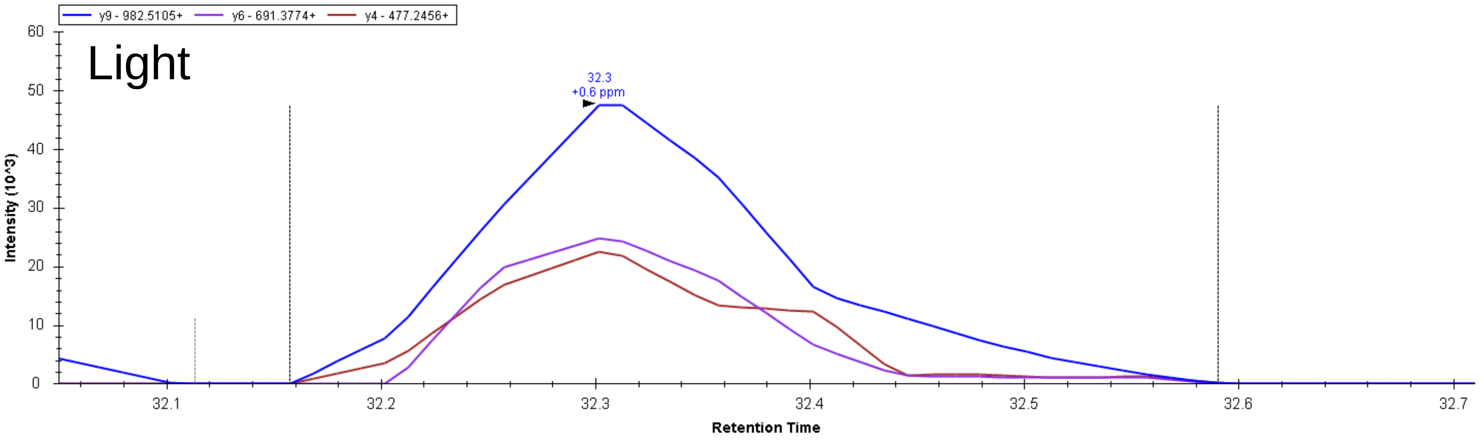

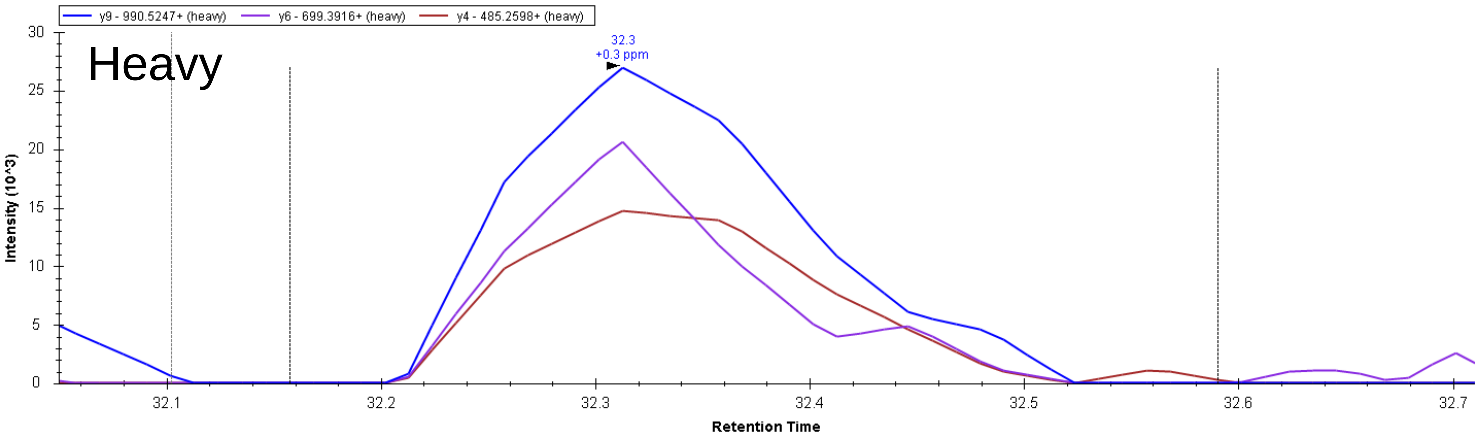

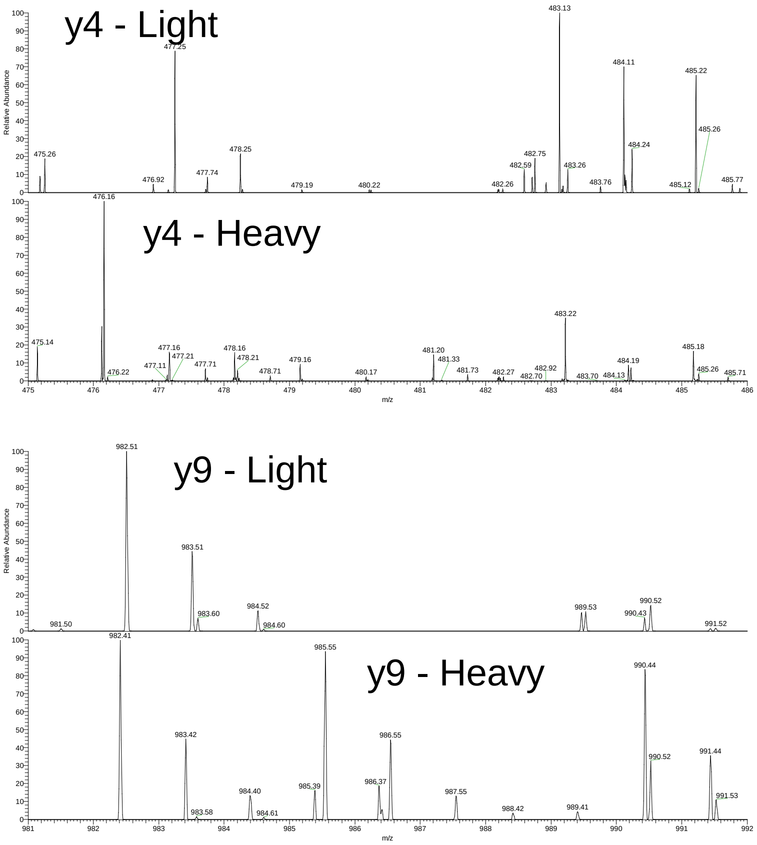

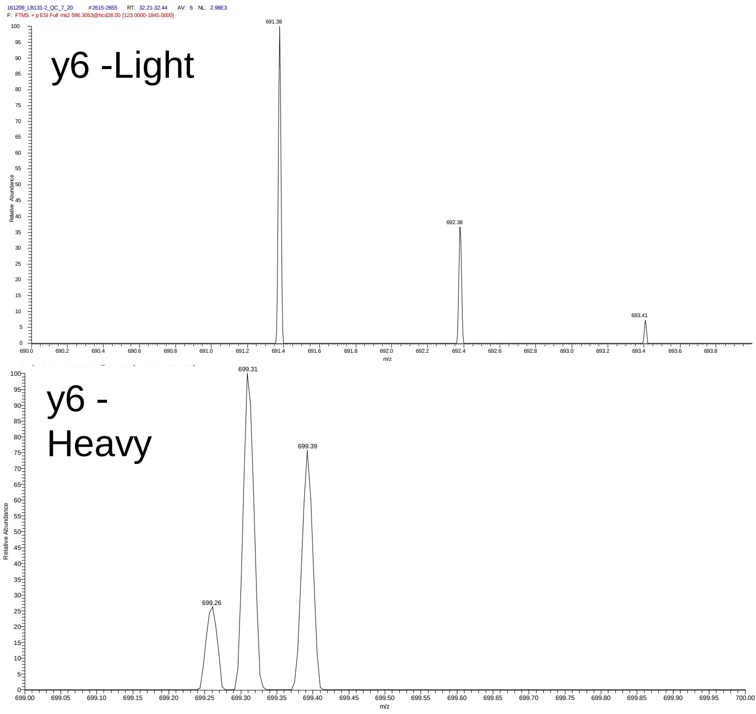

Ae; HEXB_391-400; VLDIIATINK

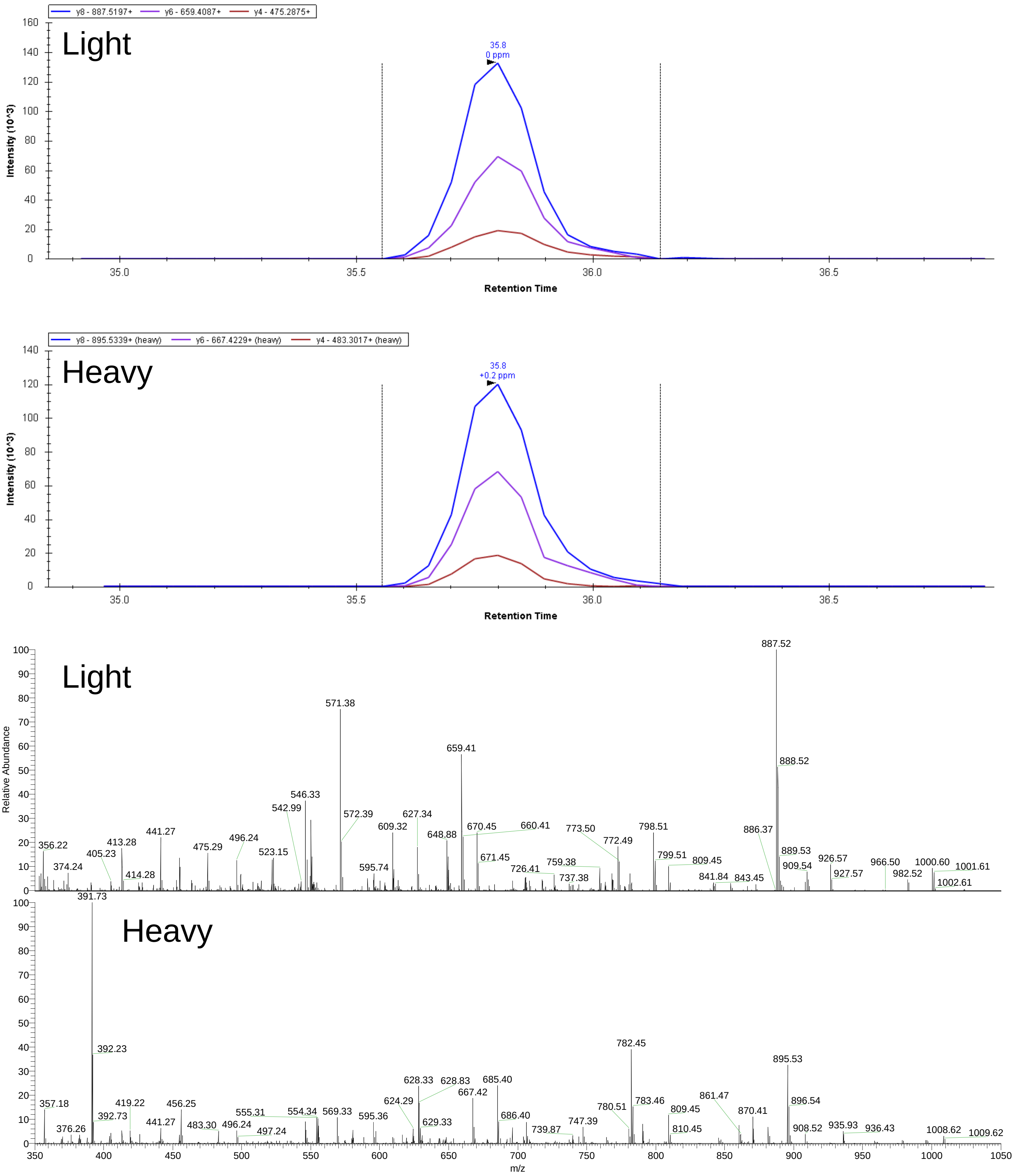

Af; LAMP1_138-146; TVESITDIR

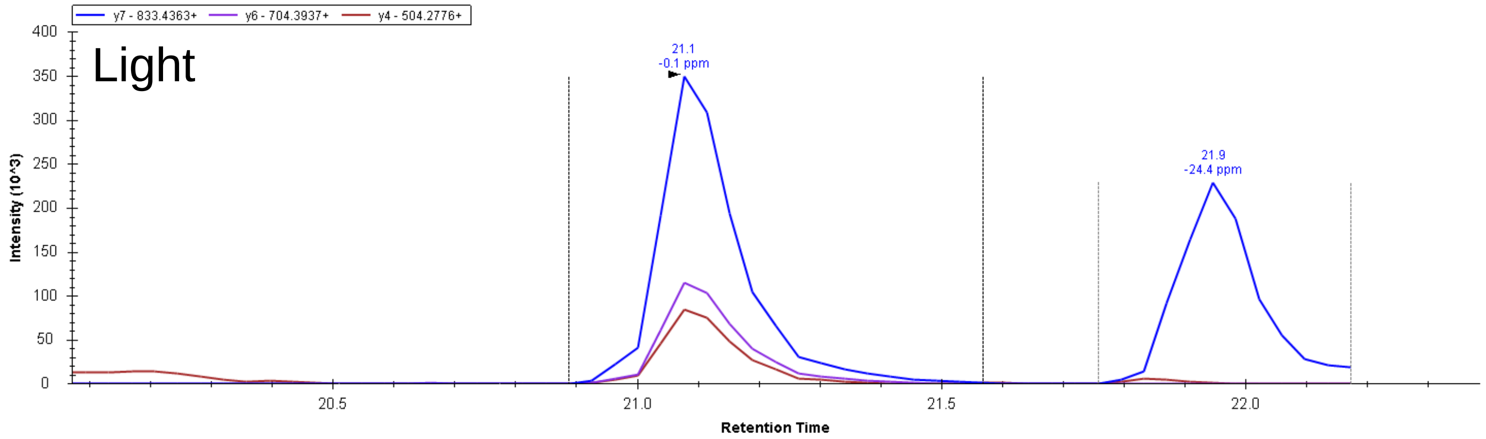

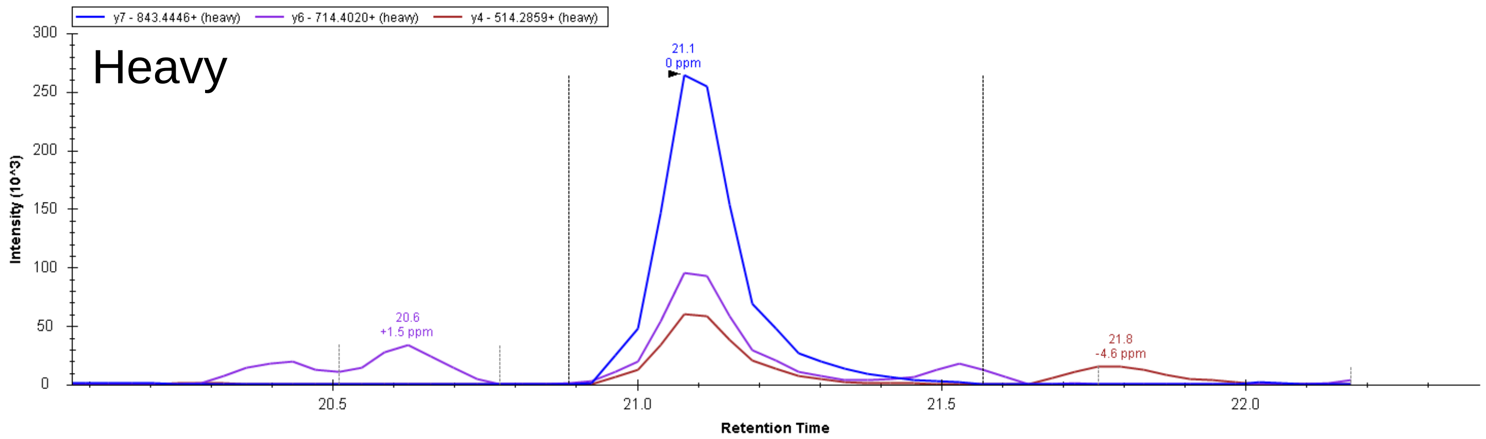

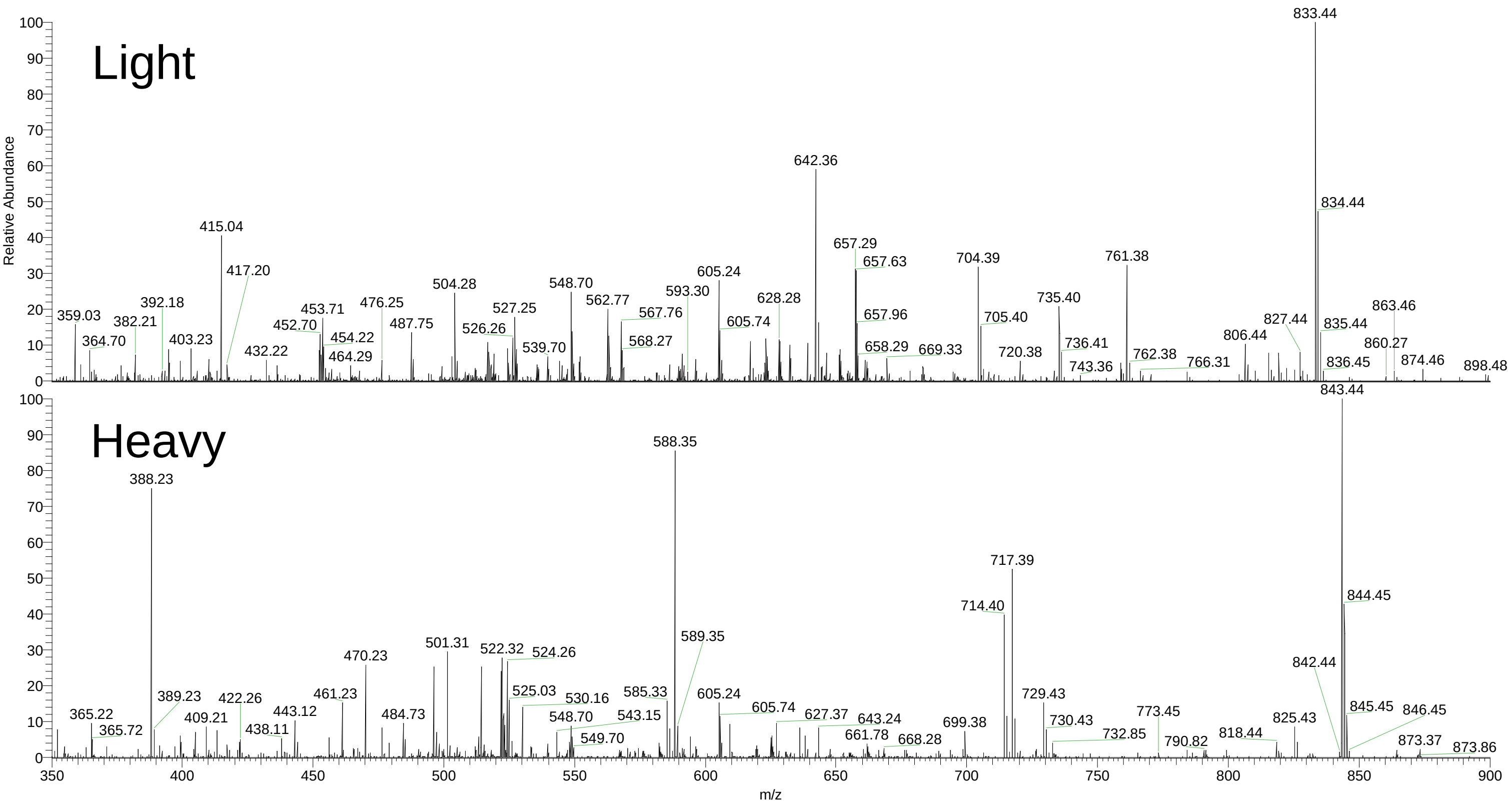

Ag; LAMP1_327-337; ALQATVGNSYK

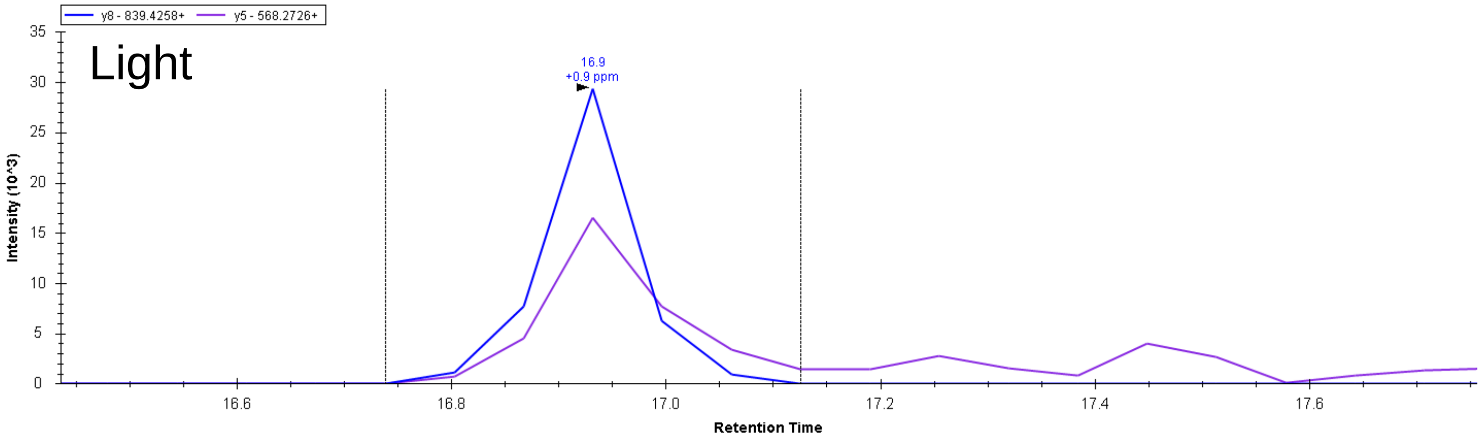

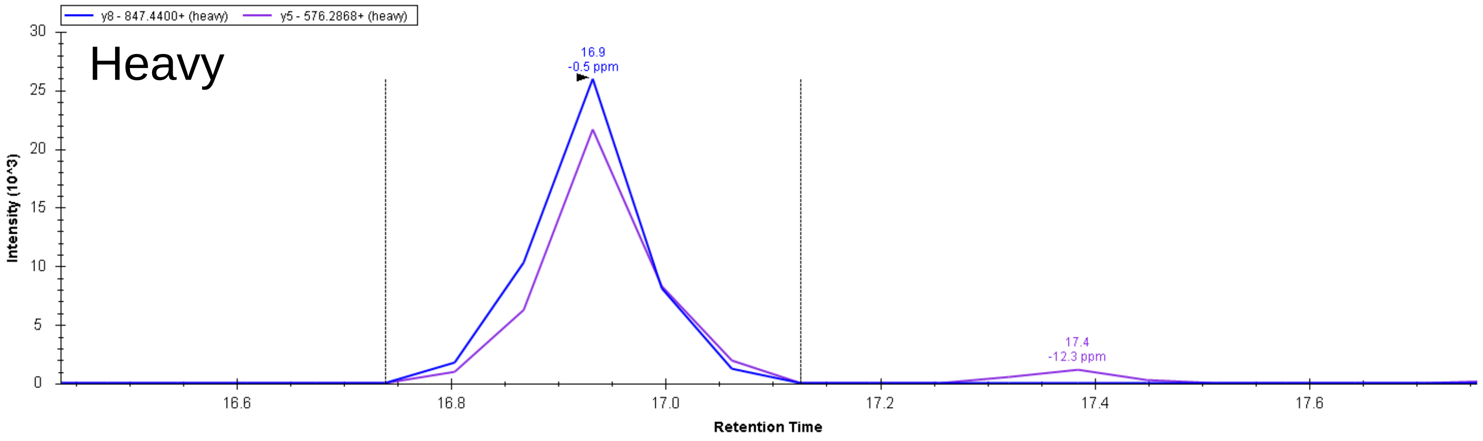

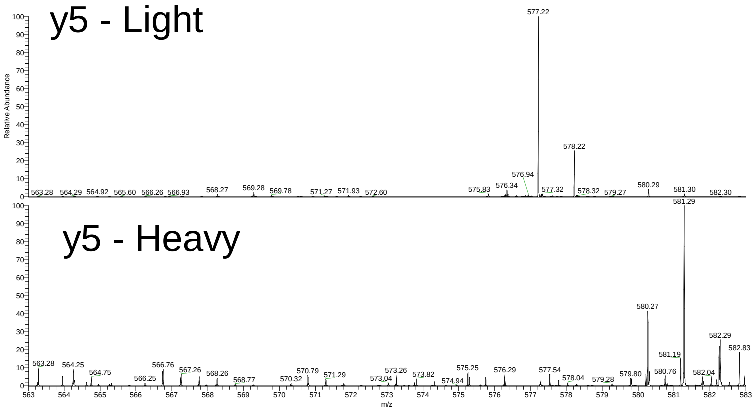

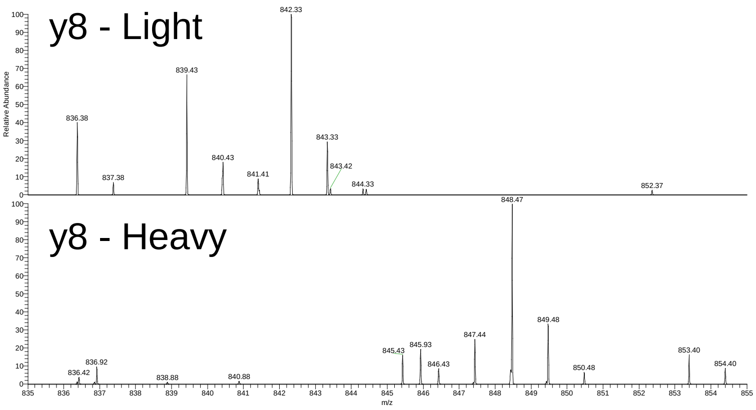

Ah; LAMP1_357-363; VWVQAFK

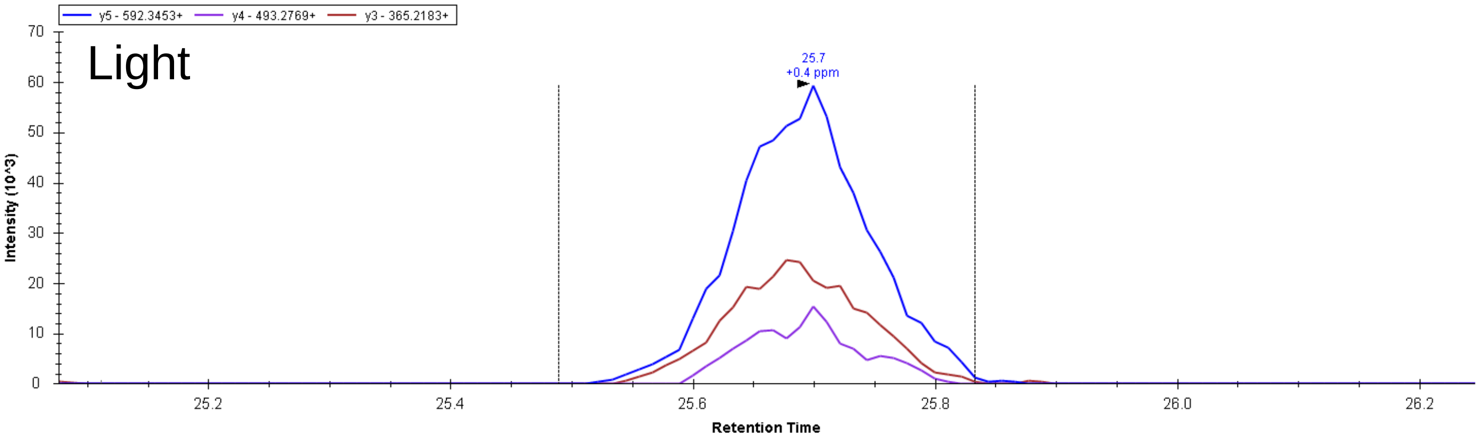

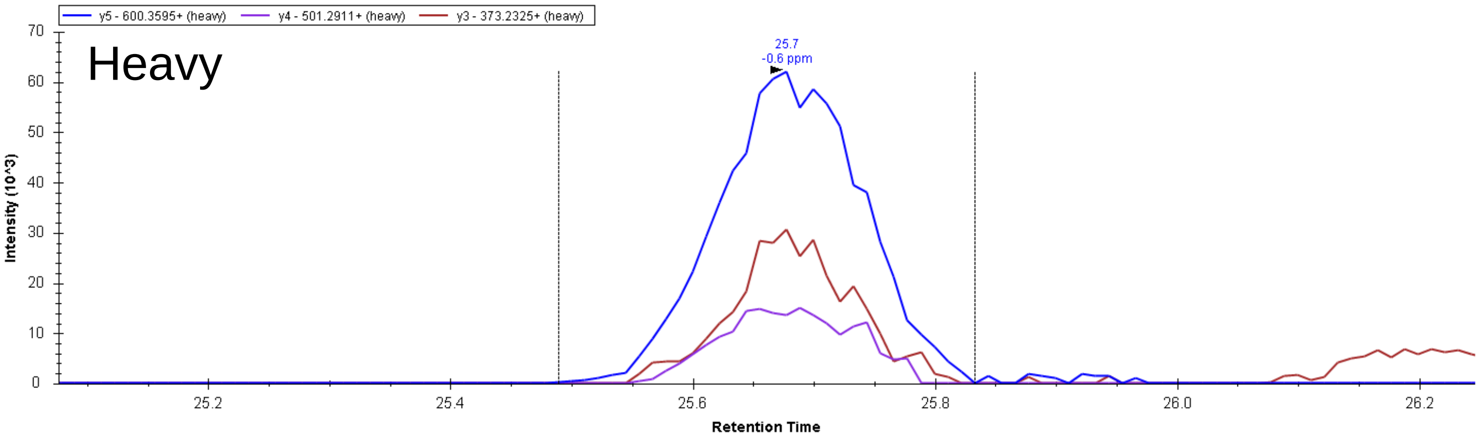

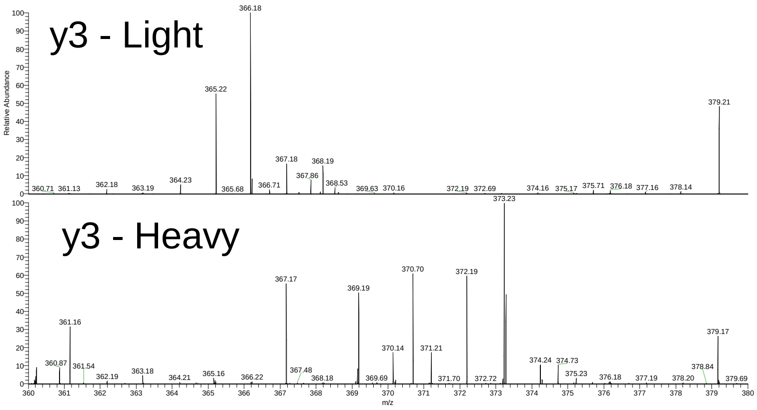

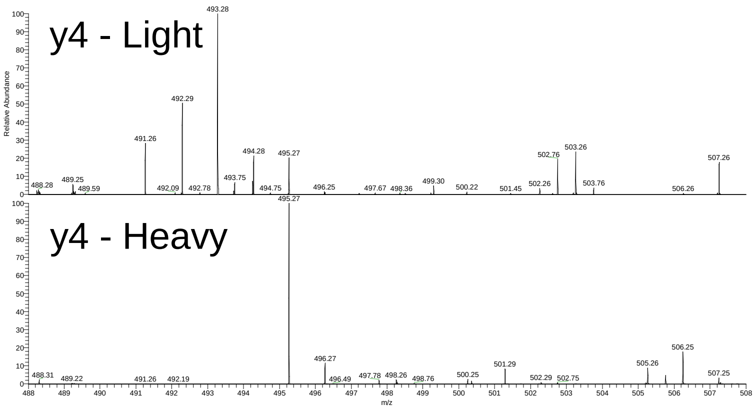

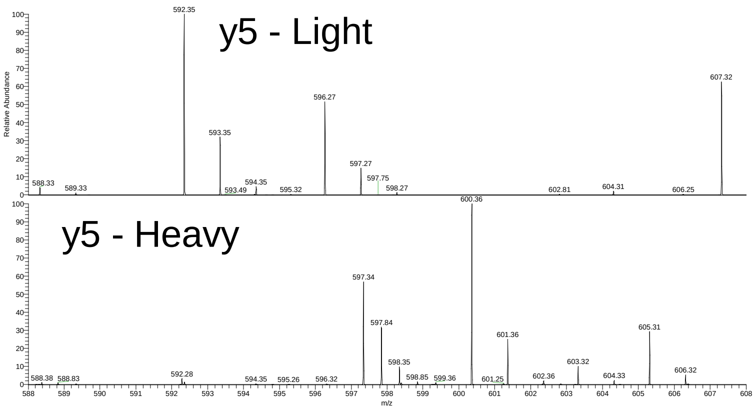

Ai; LAMP2_133-144; GILTVDELLAIR

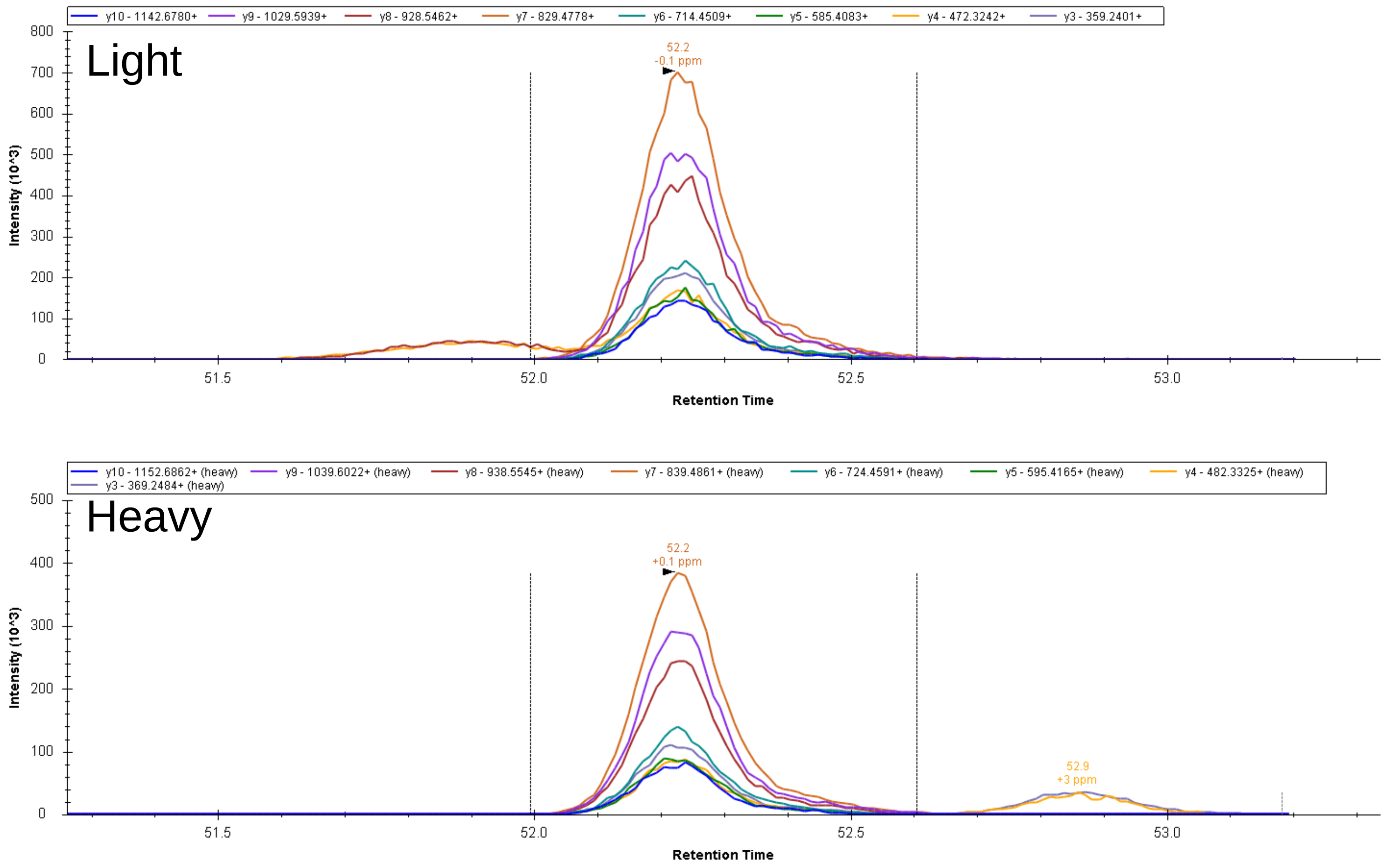

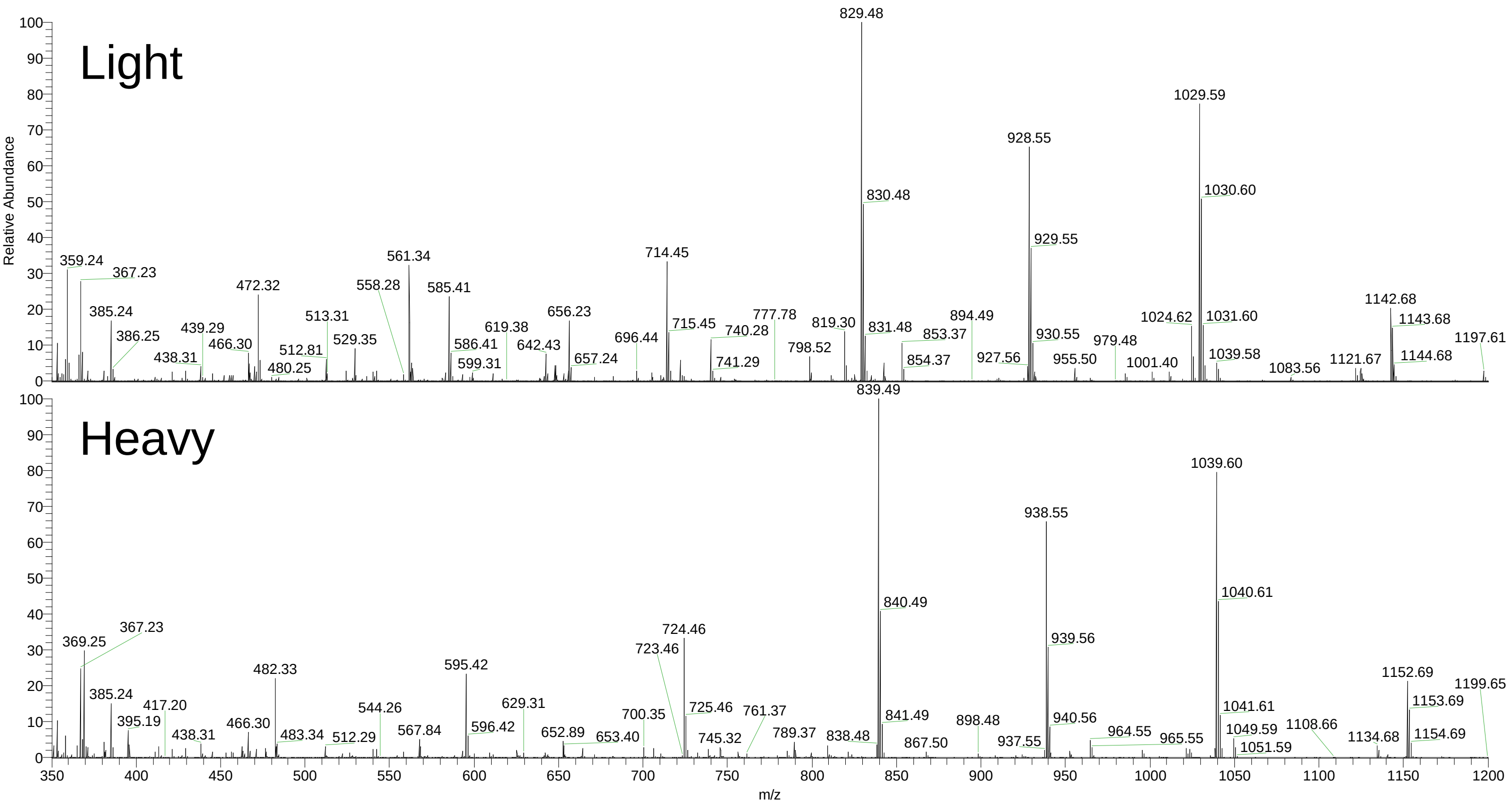

Aj; LAMP2_145-152; IPLNDLFR

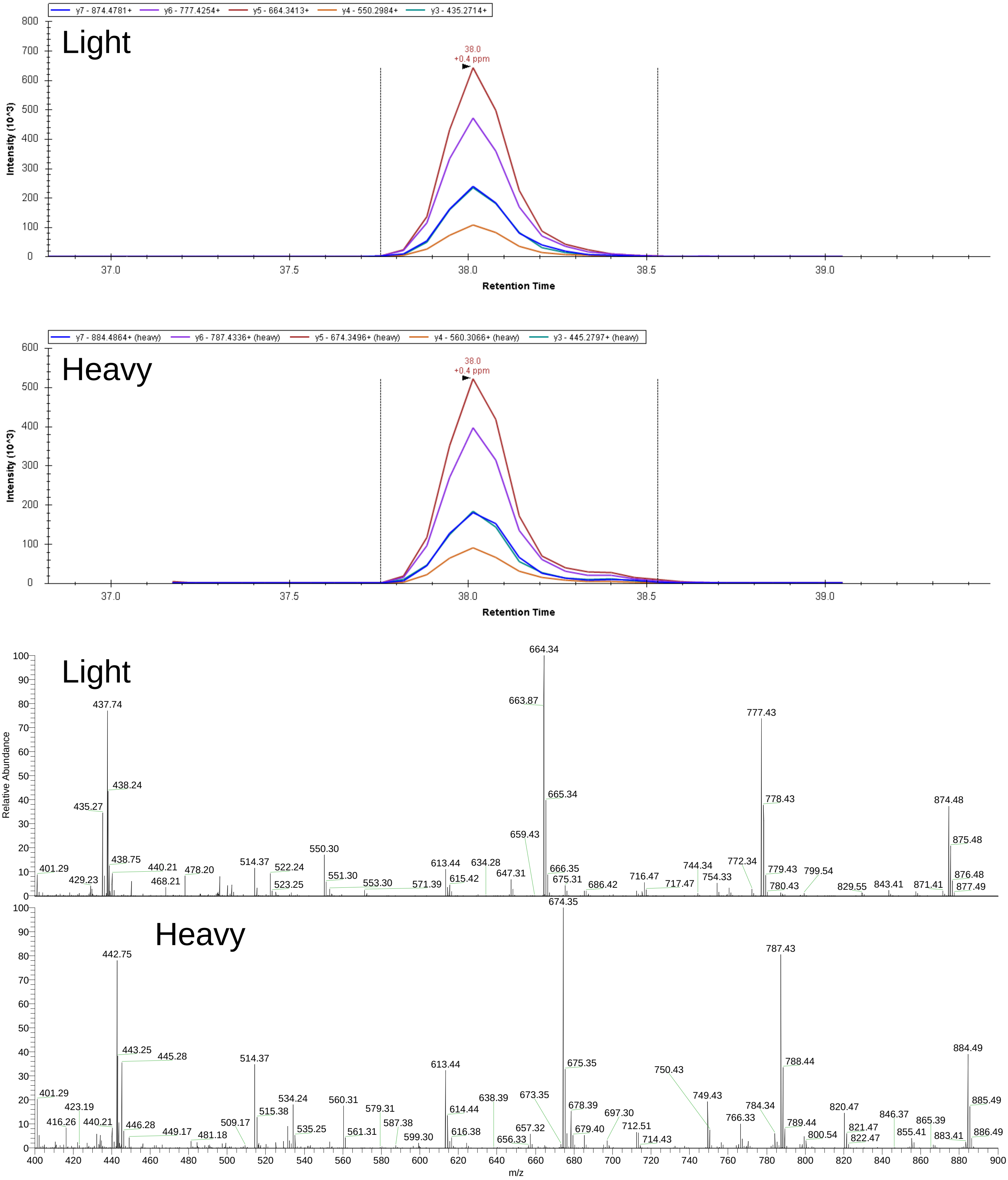

Ak; LAMP2_153-161; cNSLSTLEK

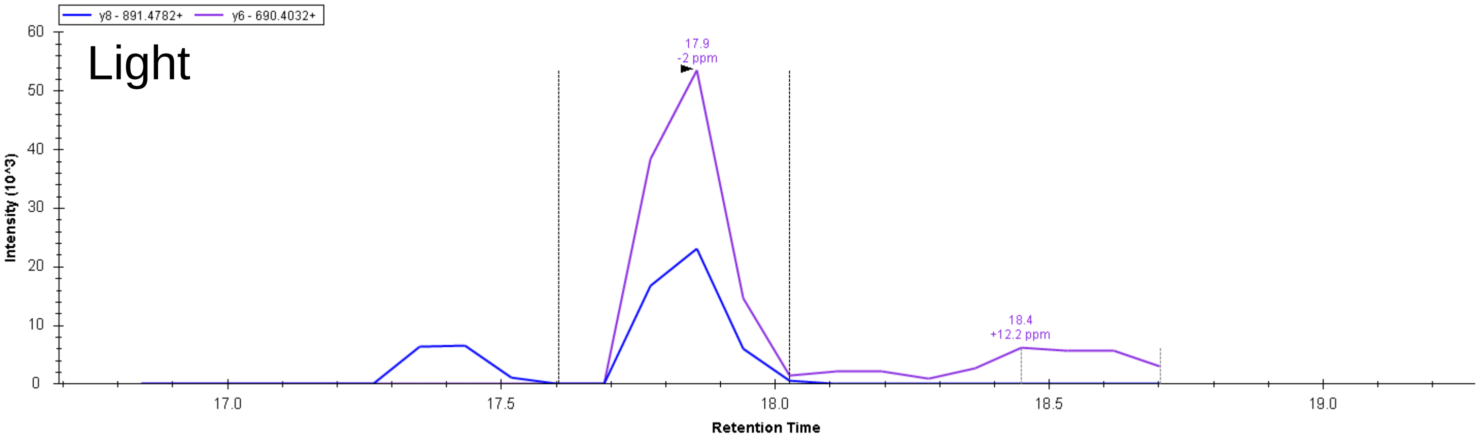

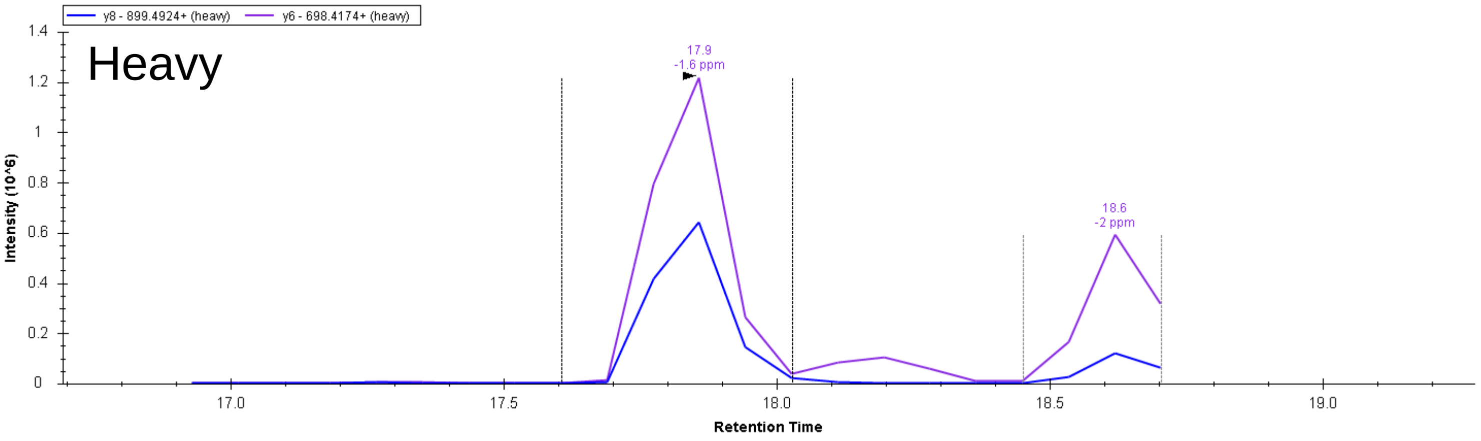

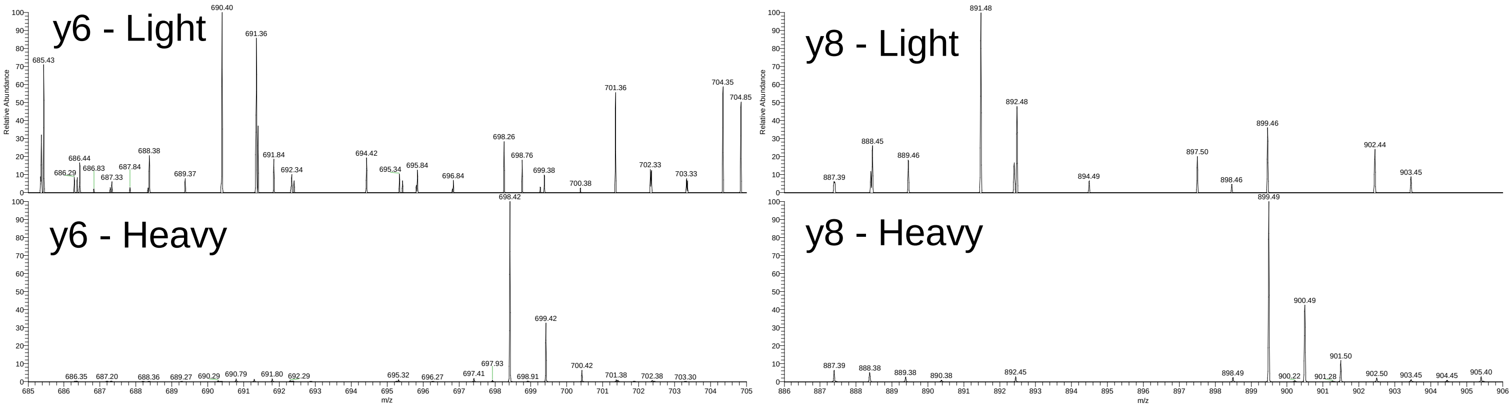

AI; LAMP2_281-289; YLDFVFAVK

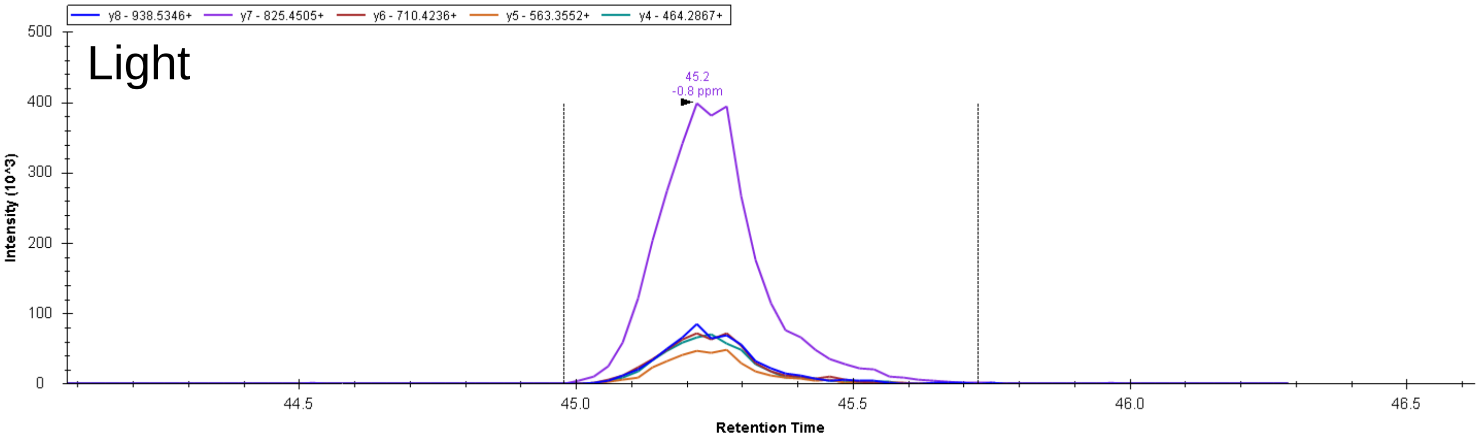

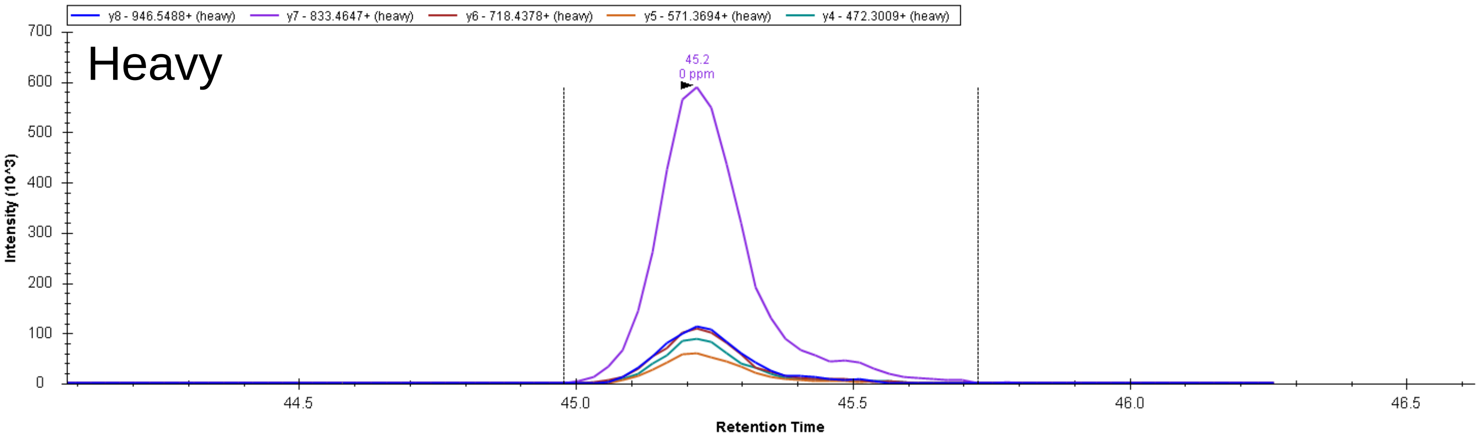

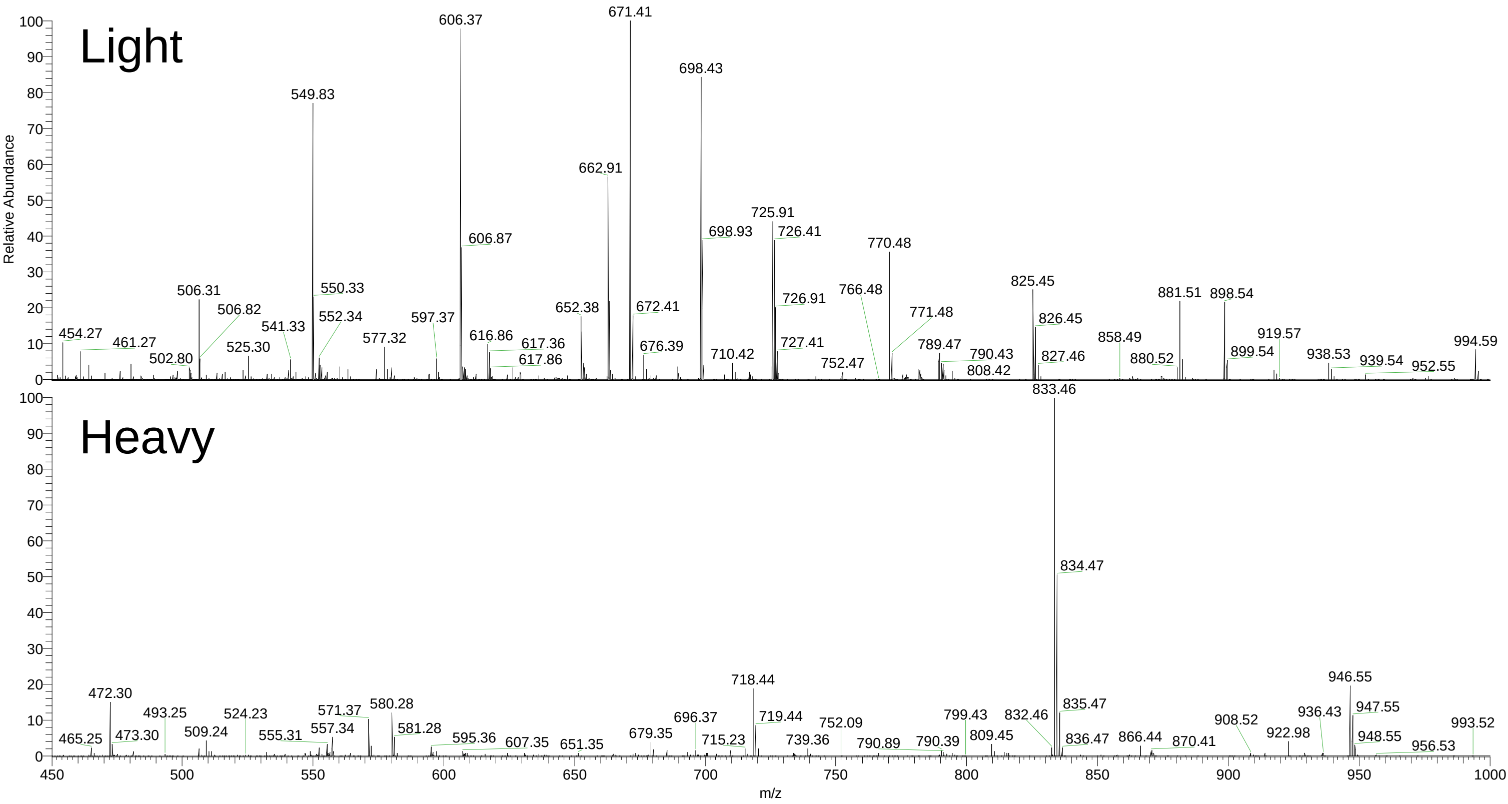

Am; LYZ_52-59; WESGYNTR

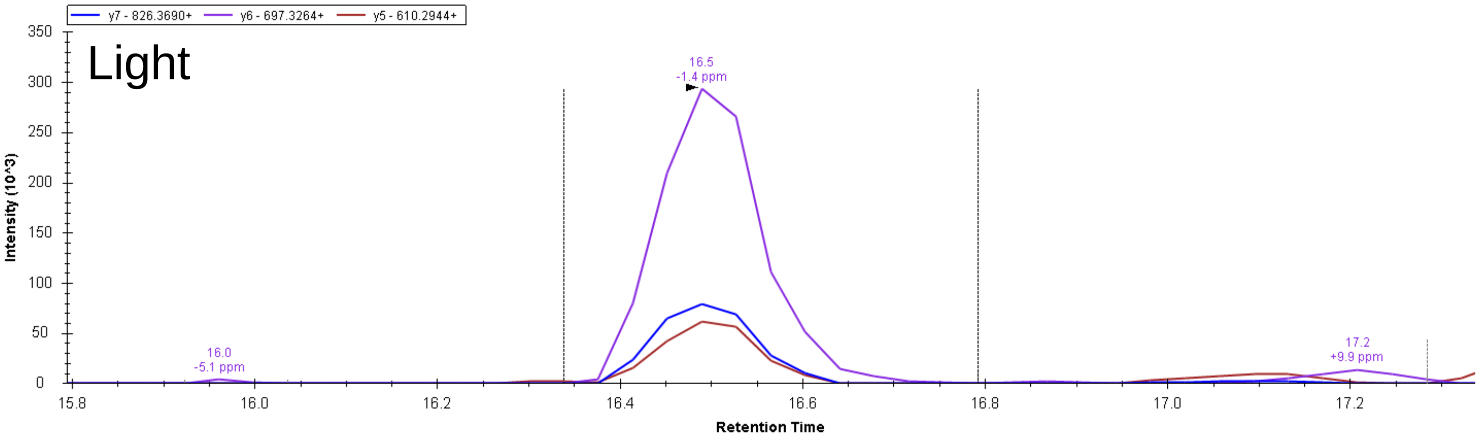

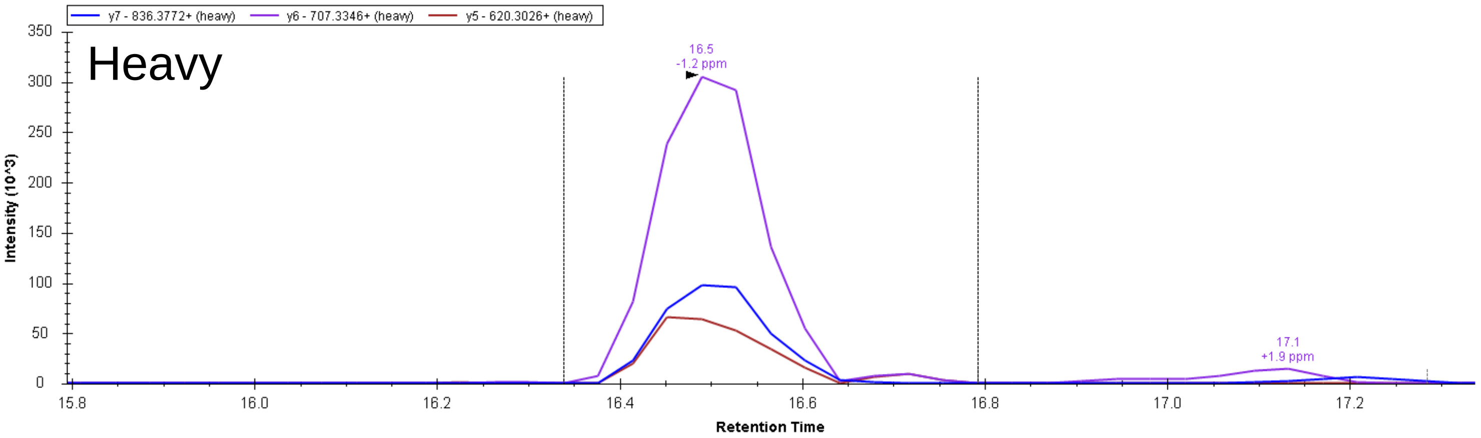

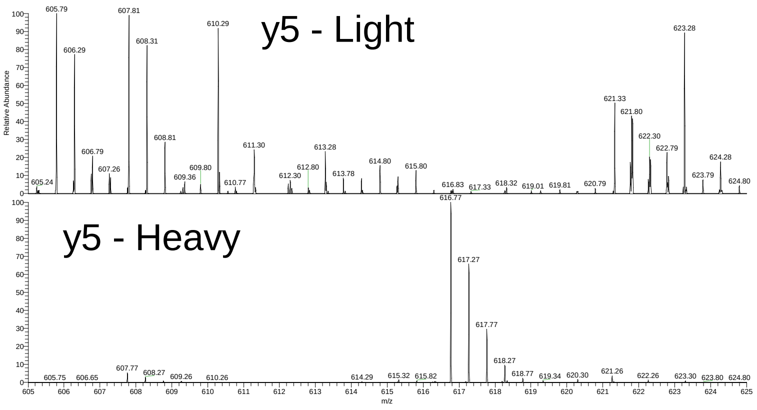

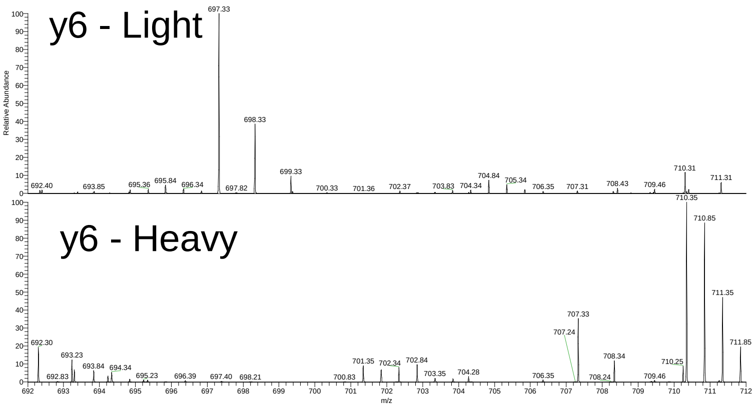

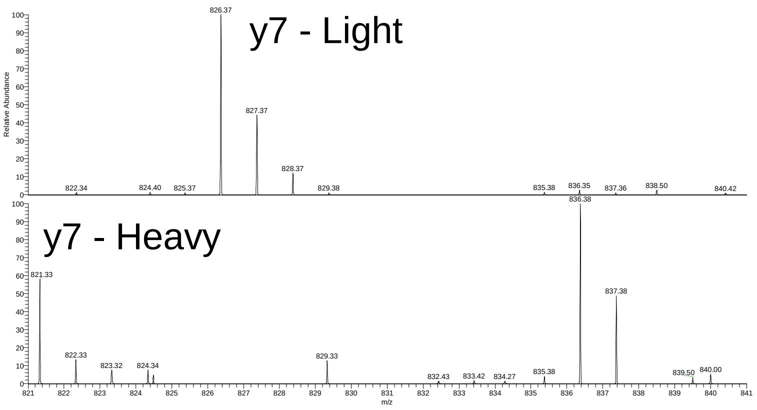

An; LYZ_69-80; STDYGIFQINSR

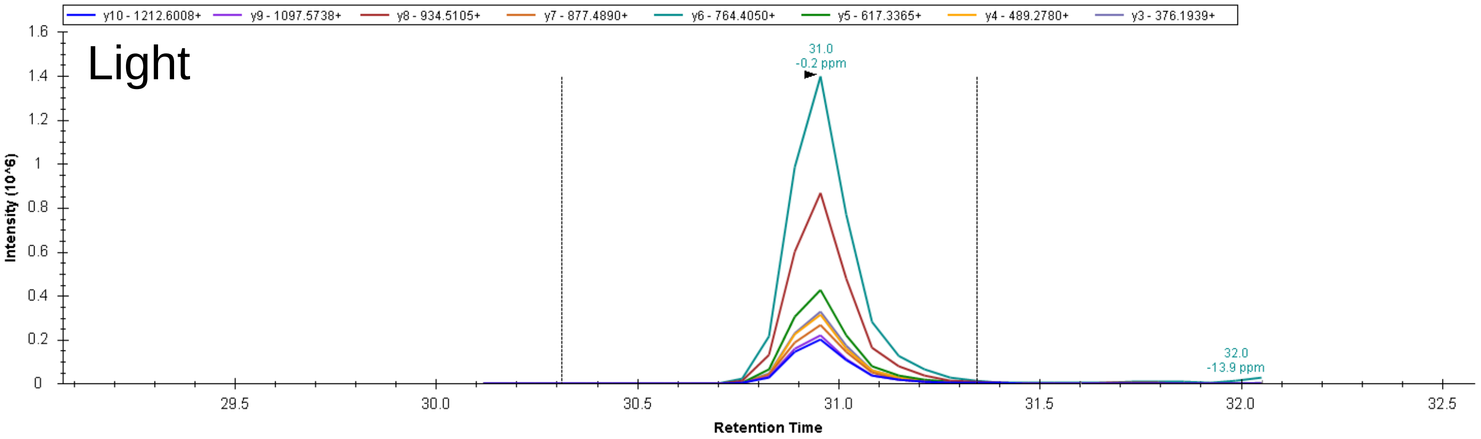

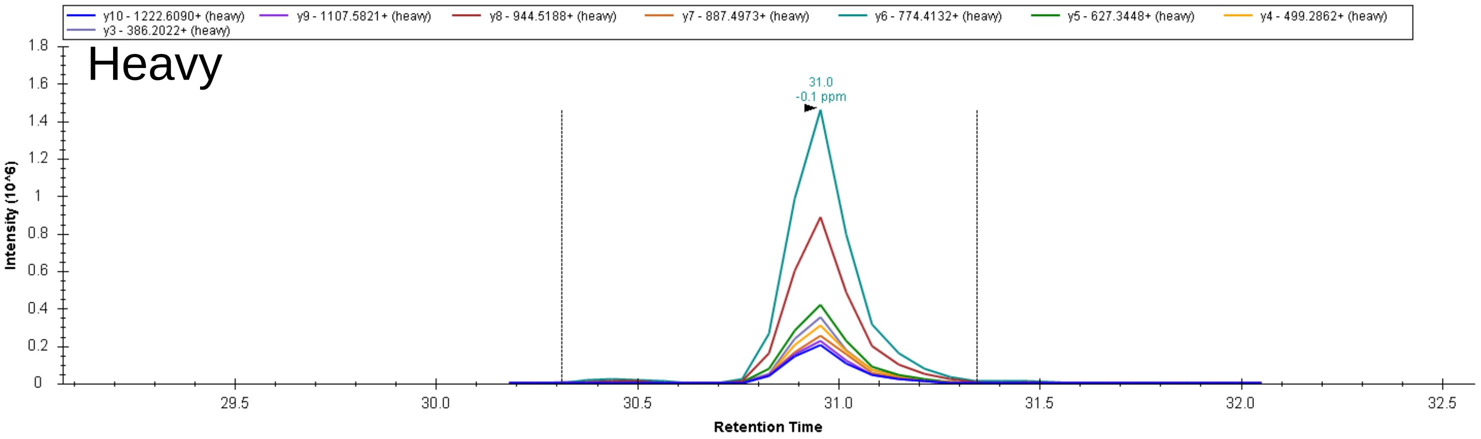

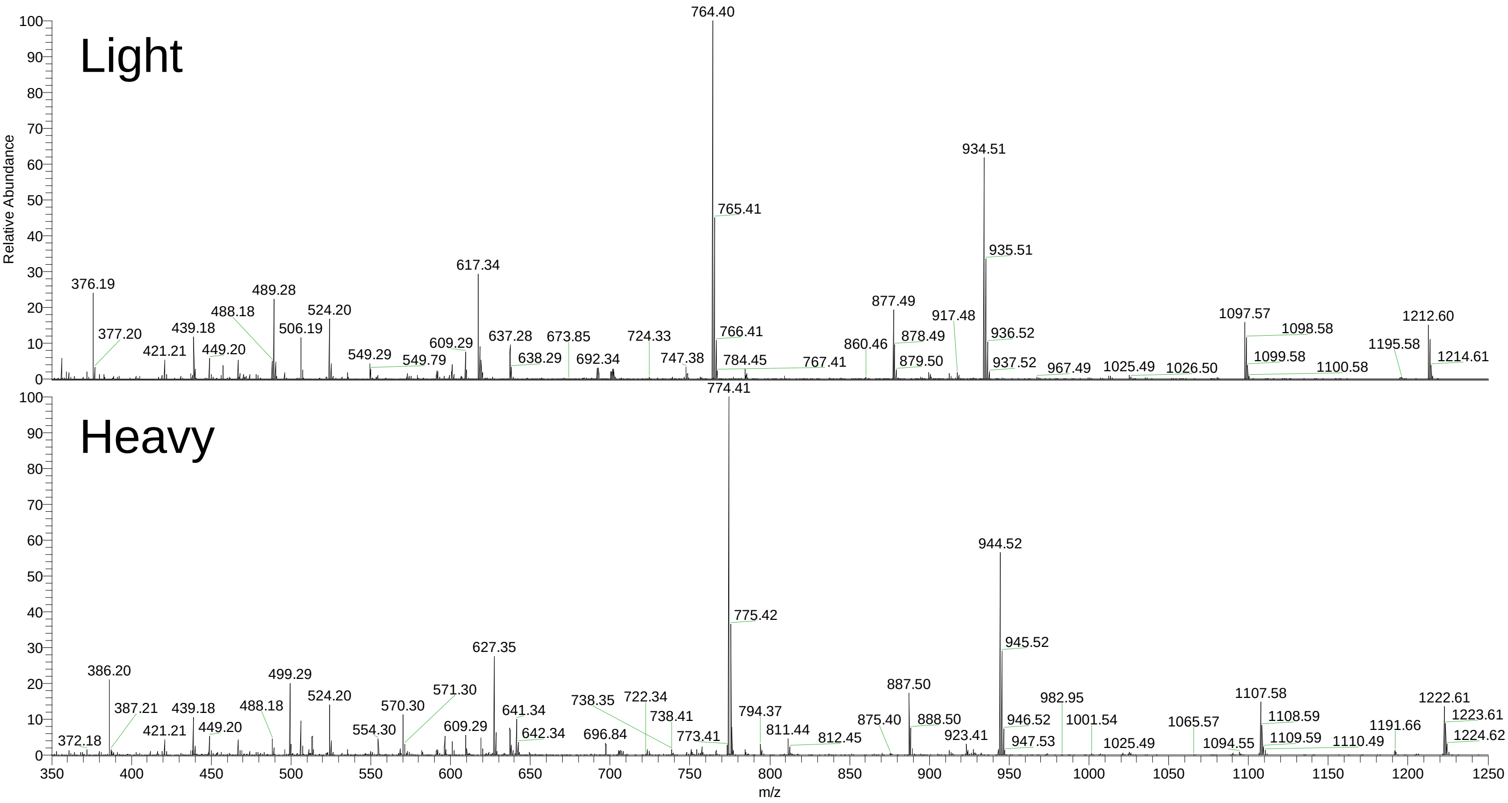

Ao; FUCA1_114-130; FFHPEEWADLFQAAGAK

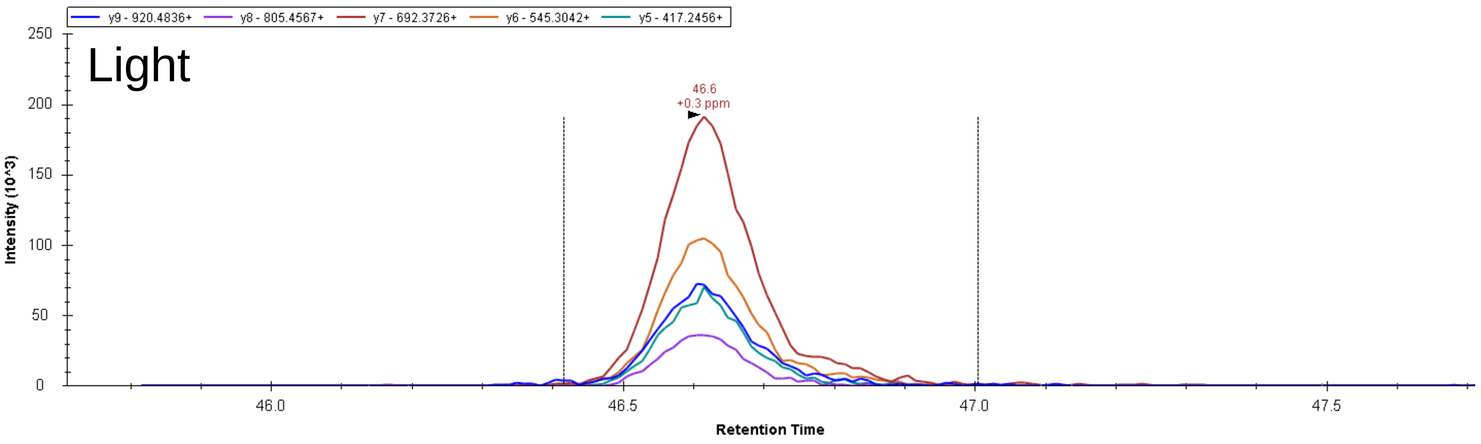

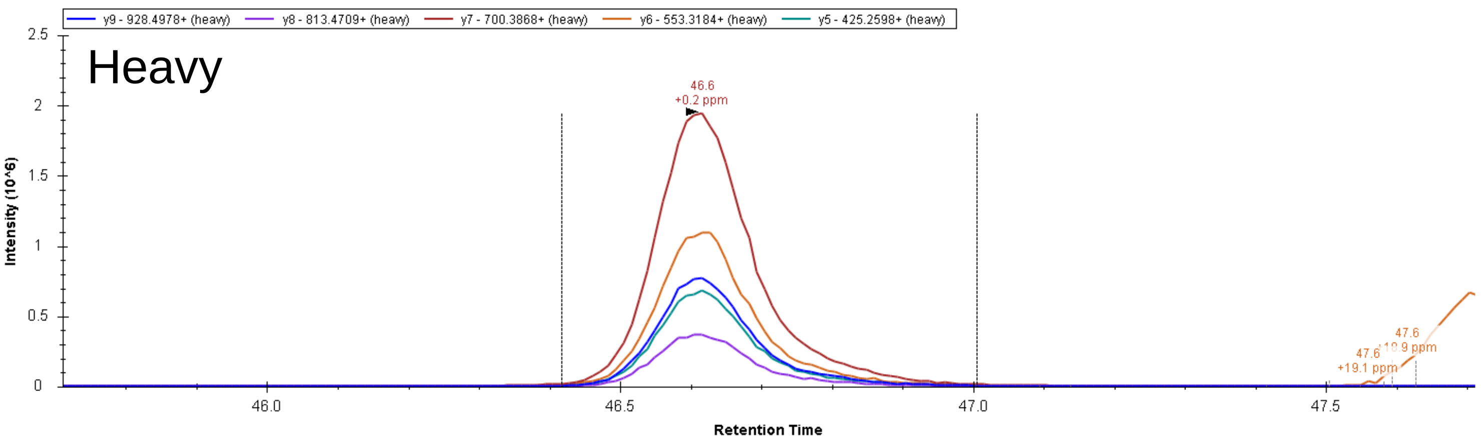

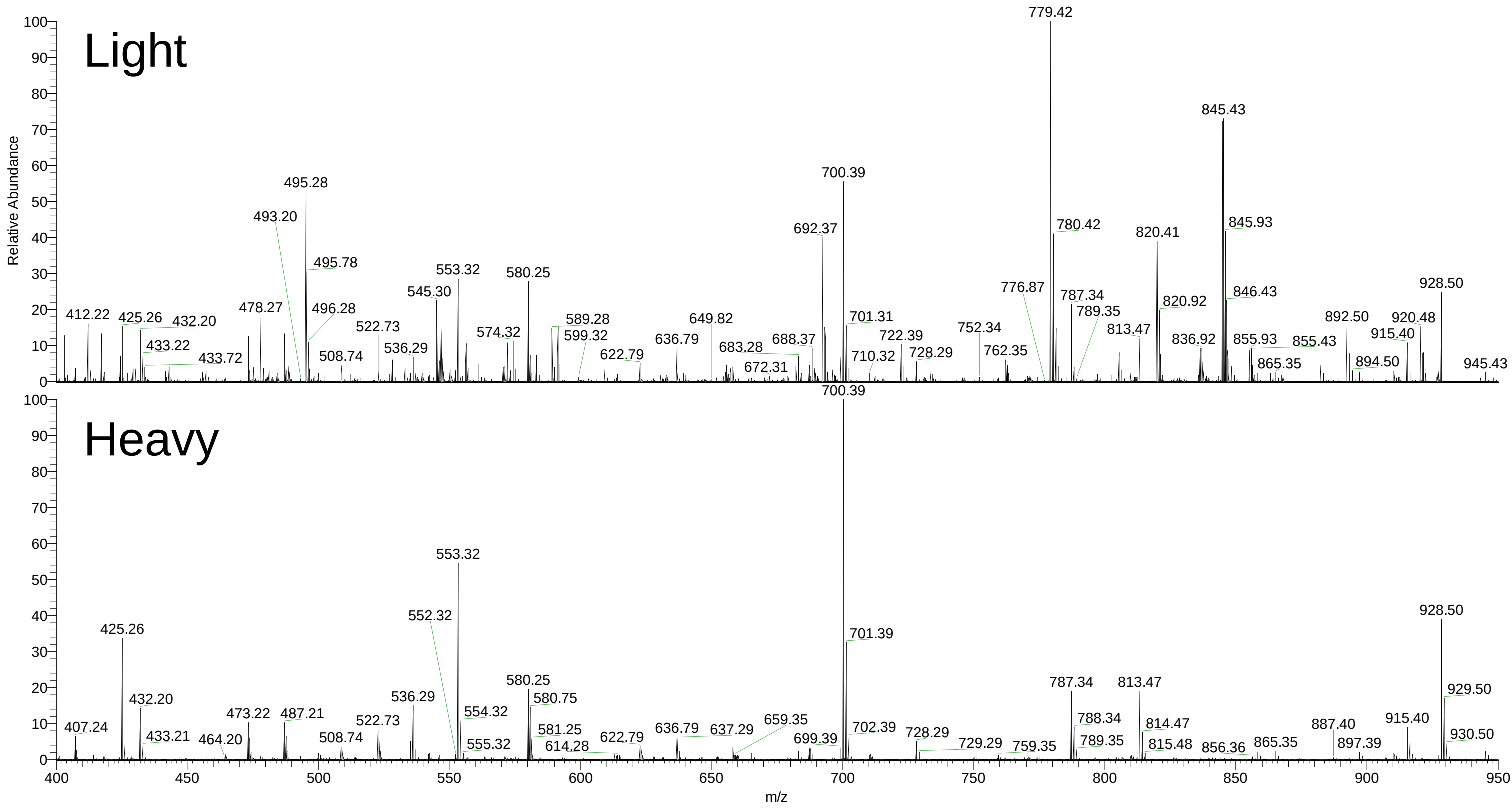

Ap; FUCA1_163-173; DLVGELGTALR

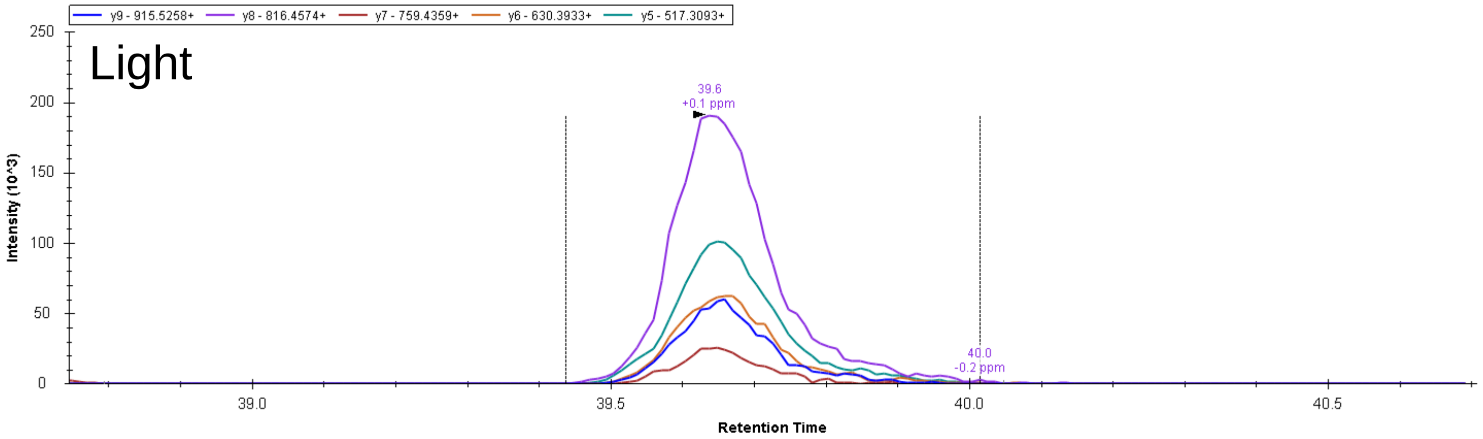

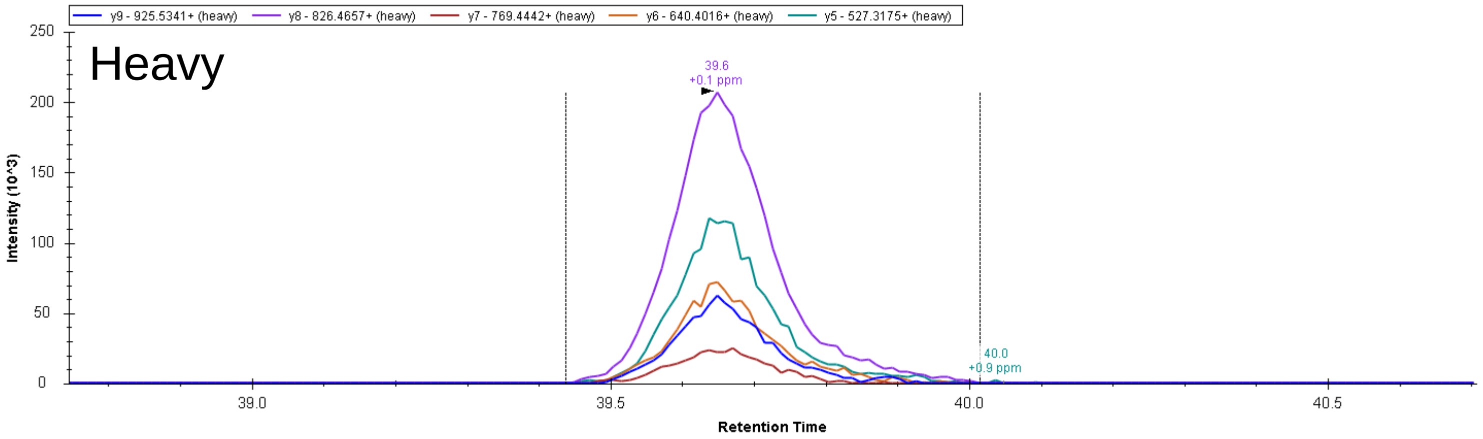

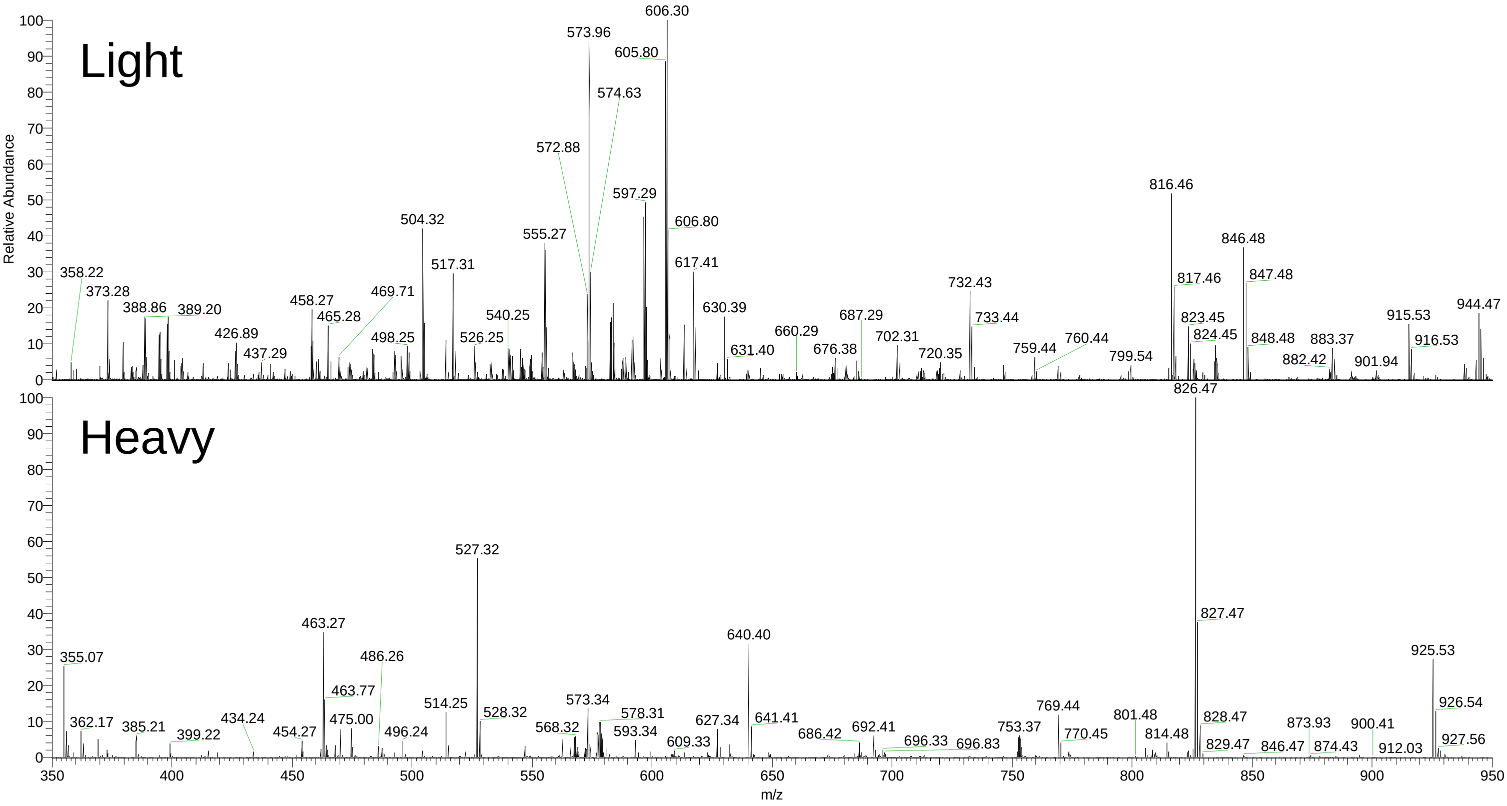

Aq; FUCA1_344-354; DGLIVPIFQER

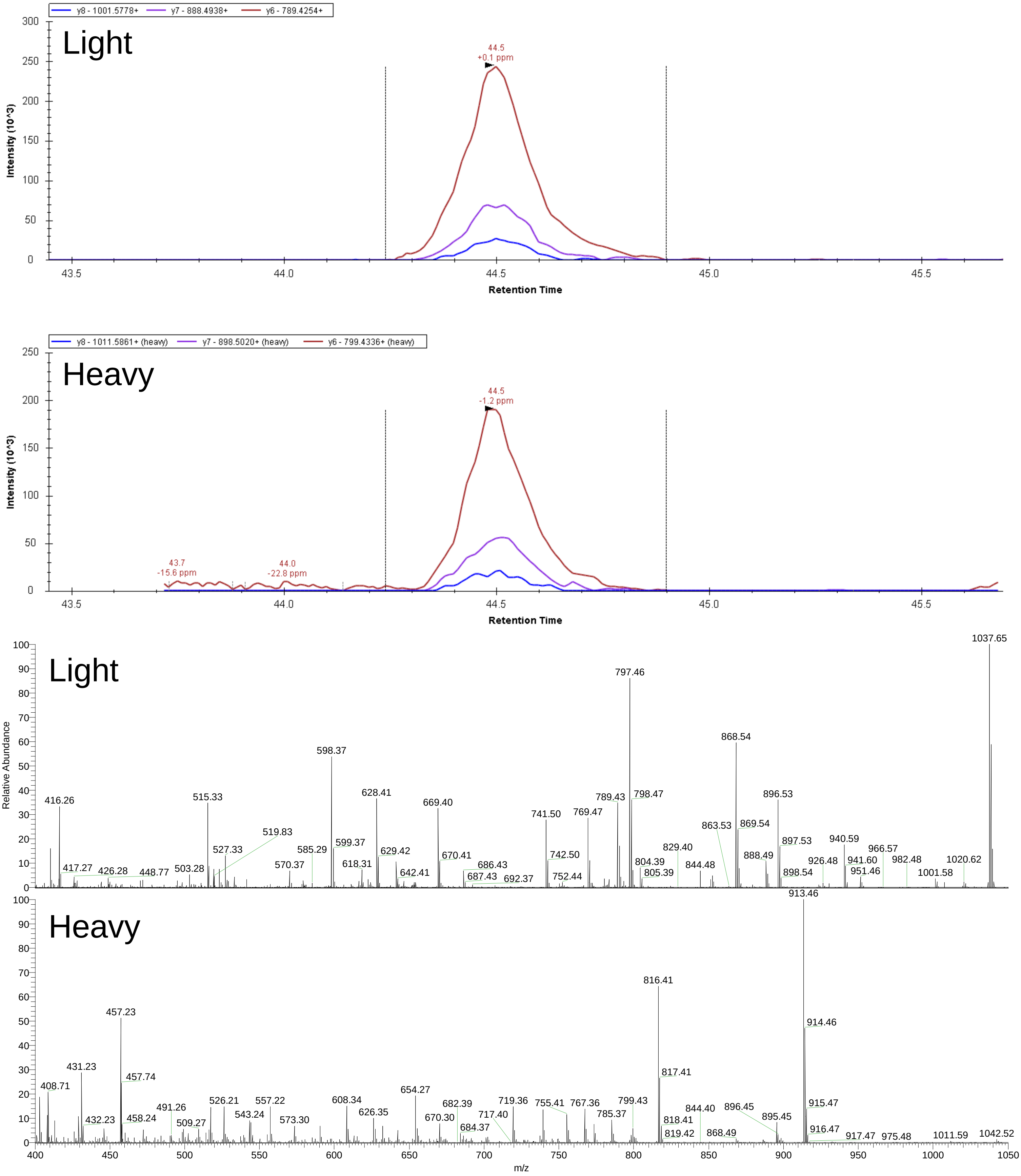

Ar; TCN2_45-49; LSLEHLNPSIYVGLR

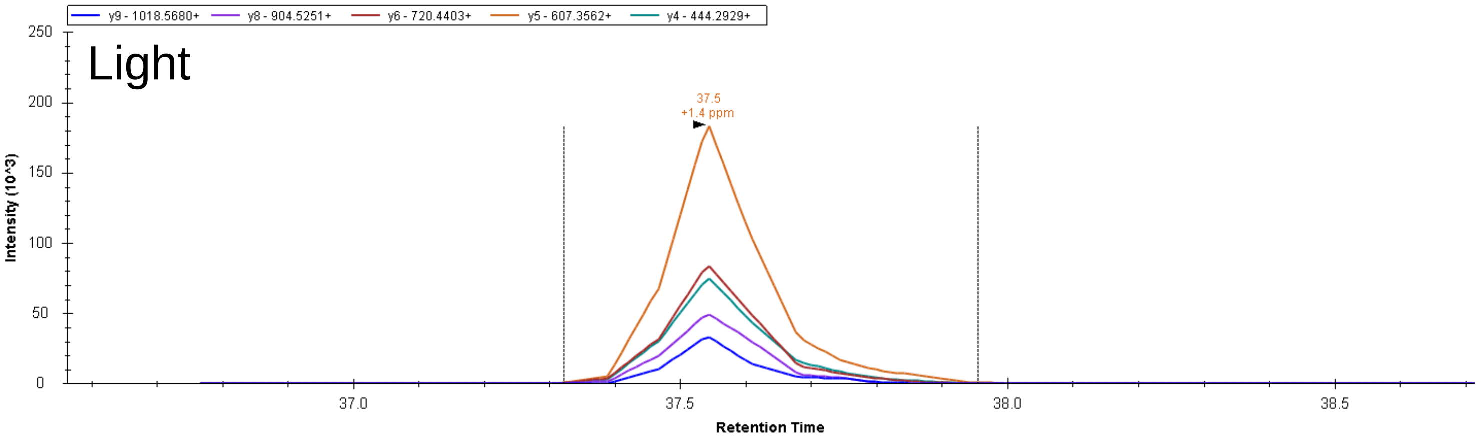

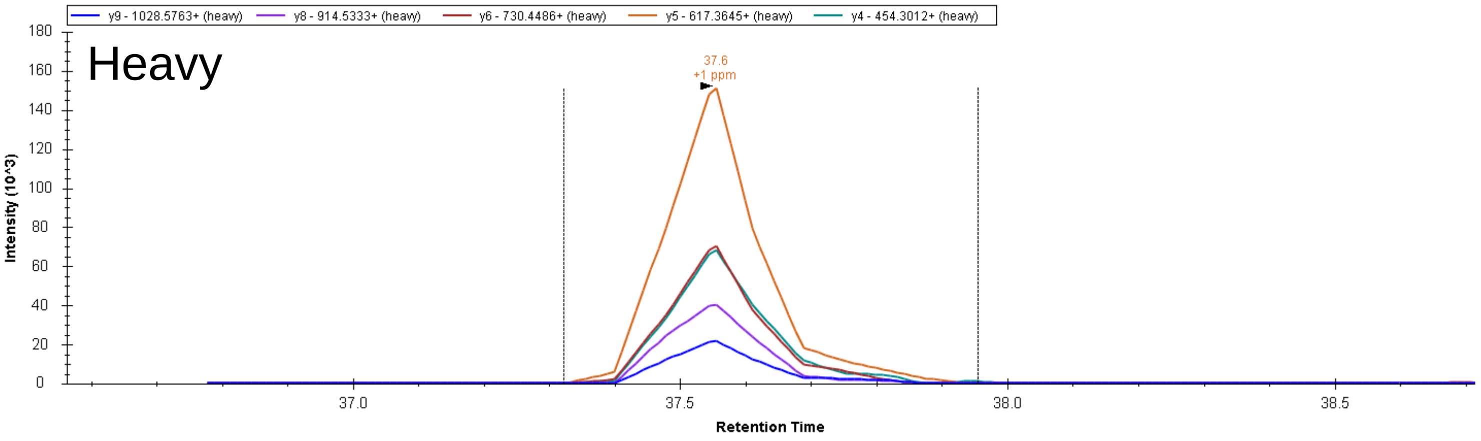

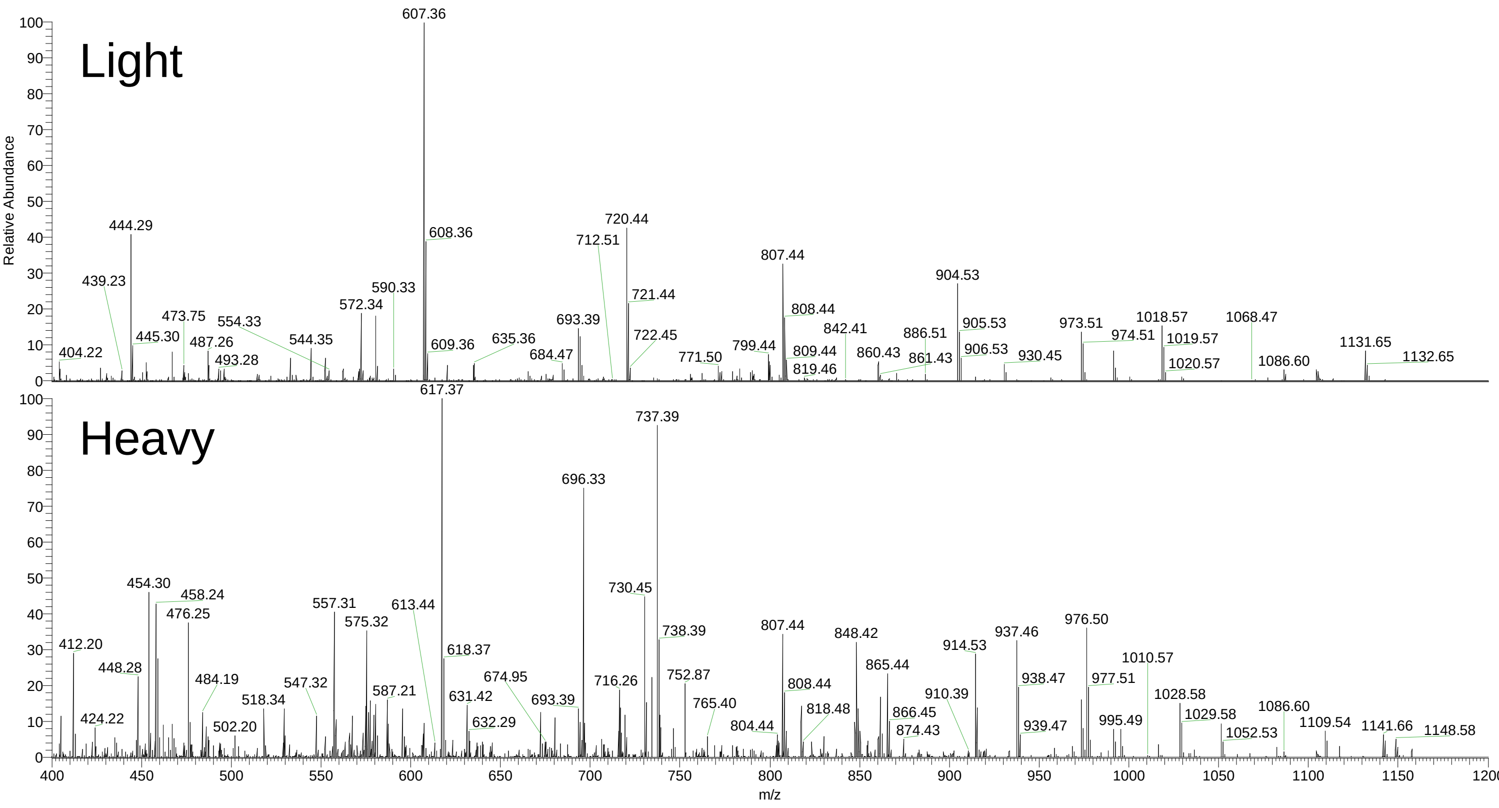

As; TCN2_300-313; TYIDLIFPDcLAPR

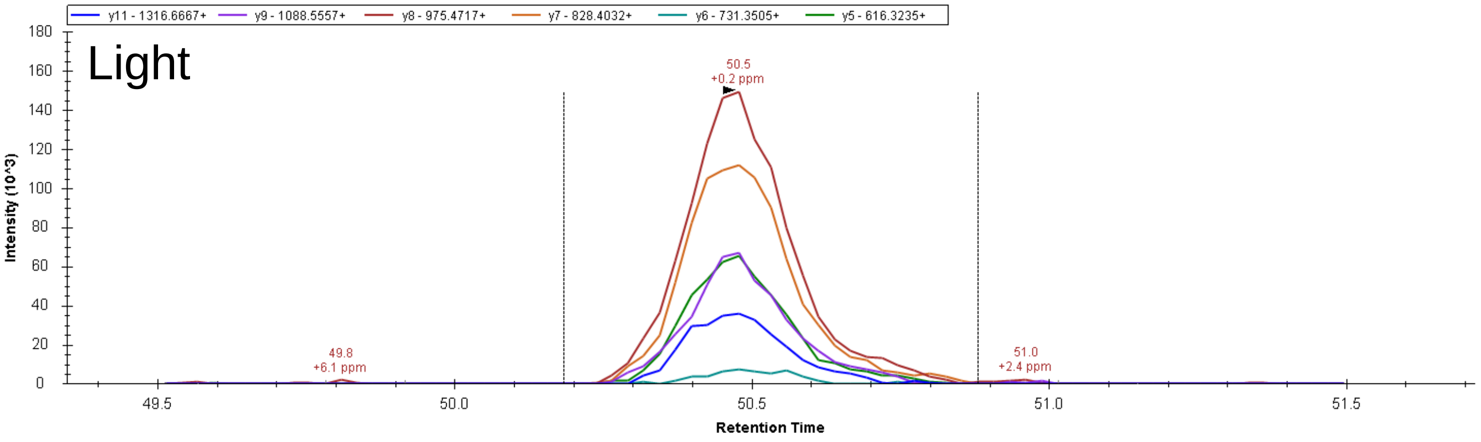

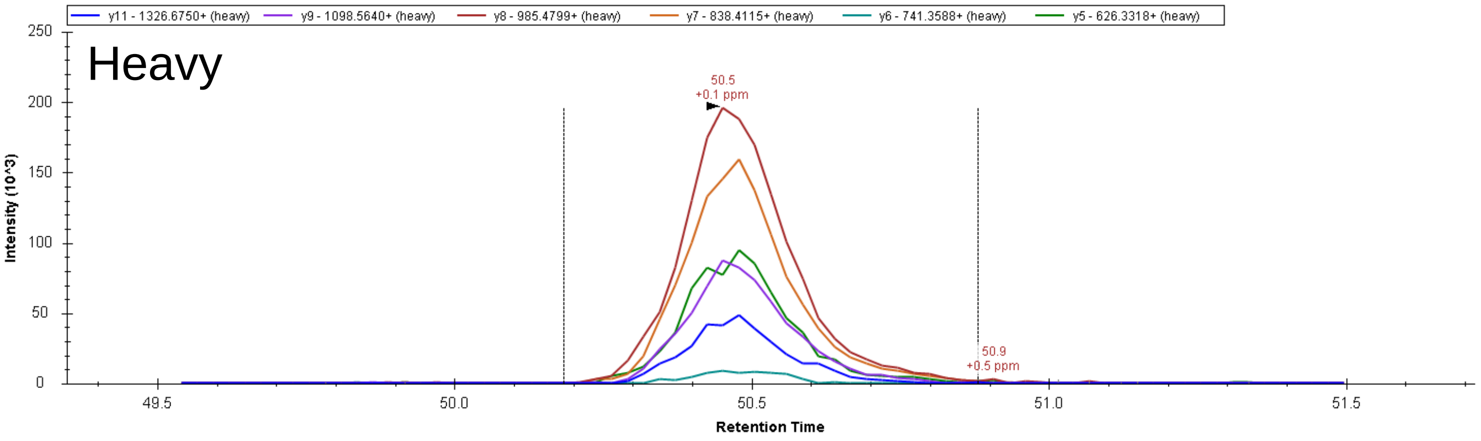

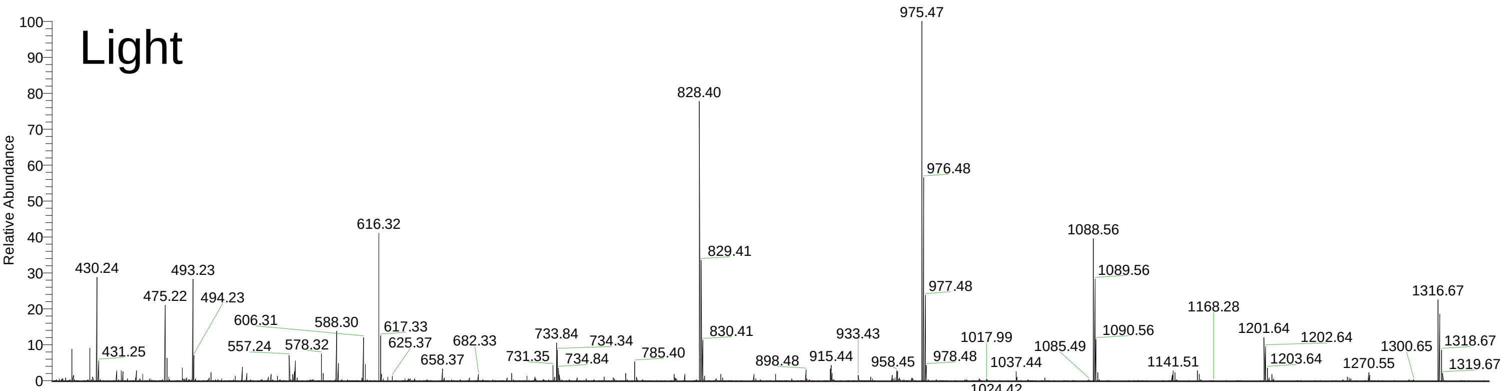

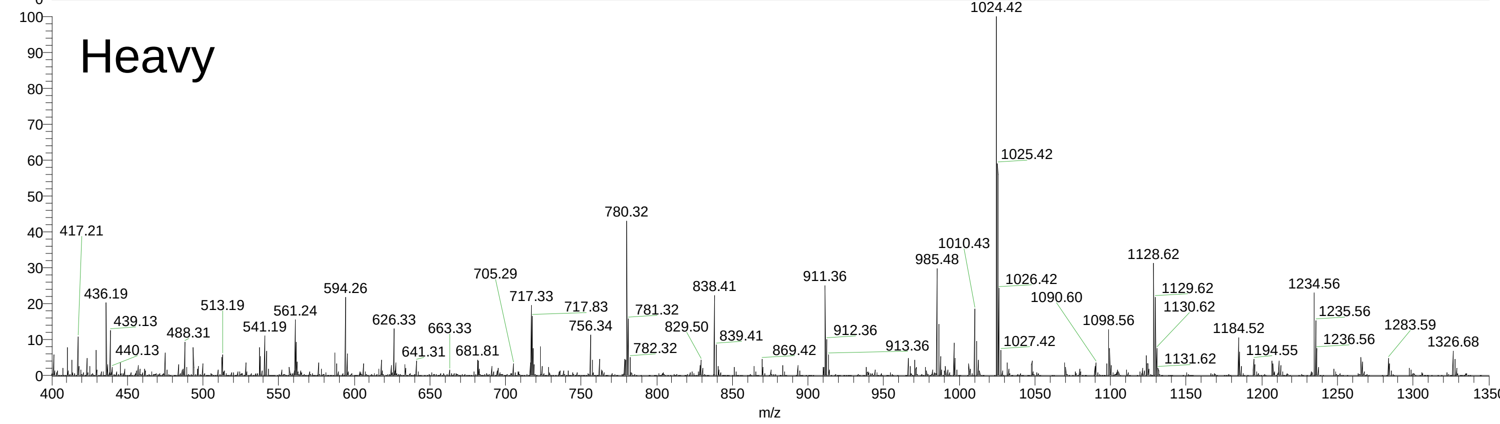

At; TCN2_393-399; EFWQLLR

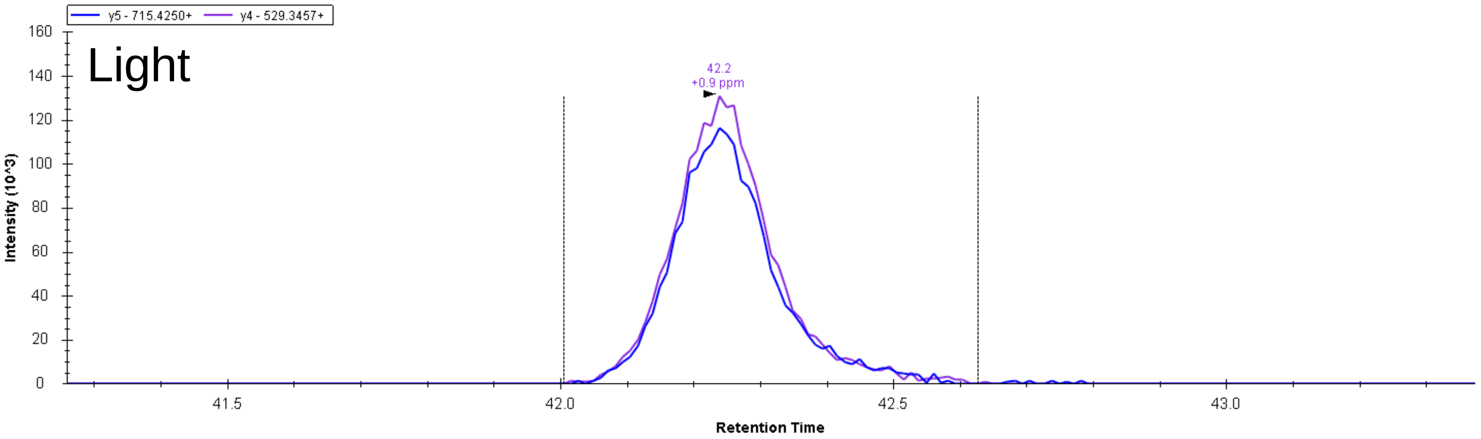

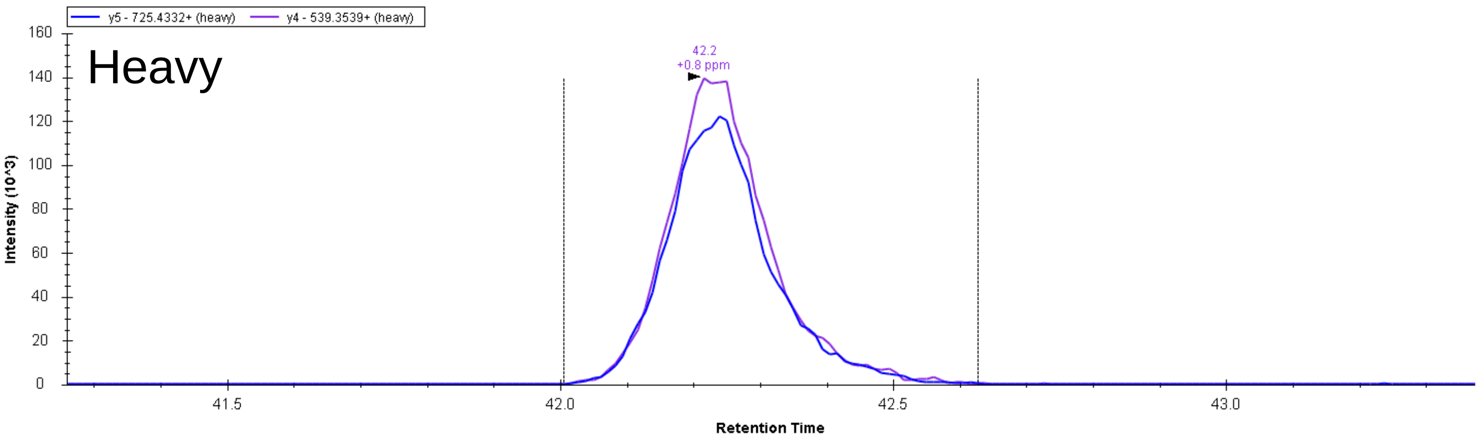

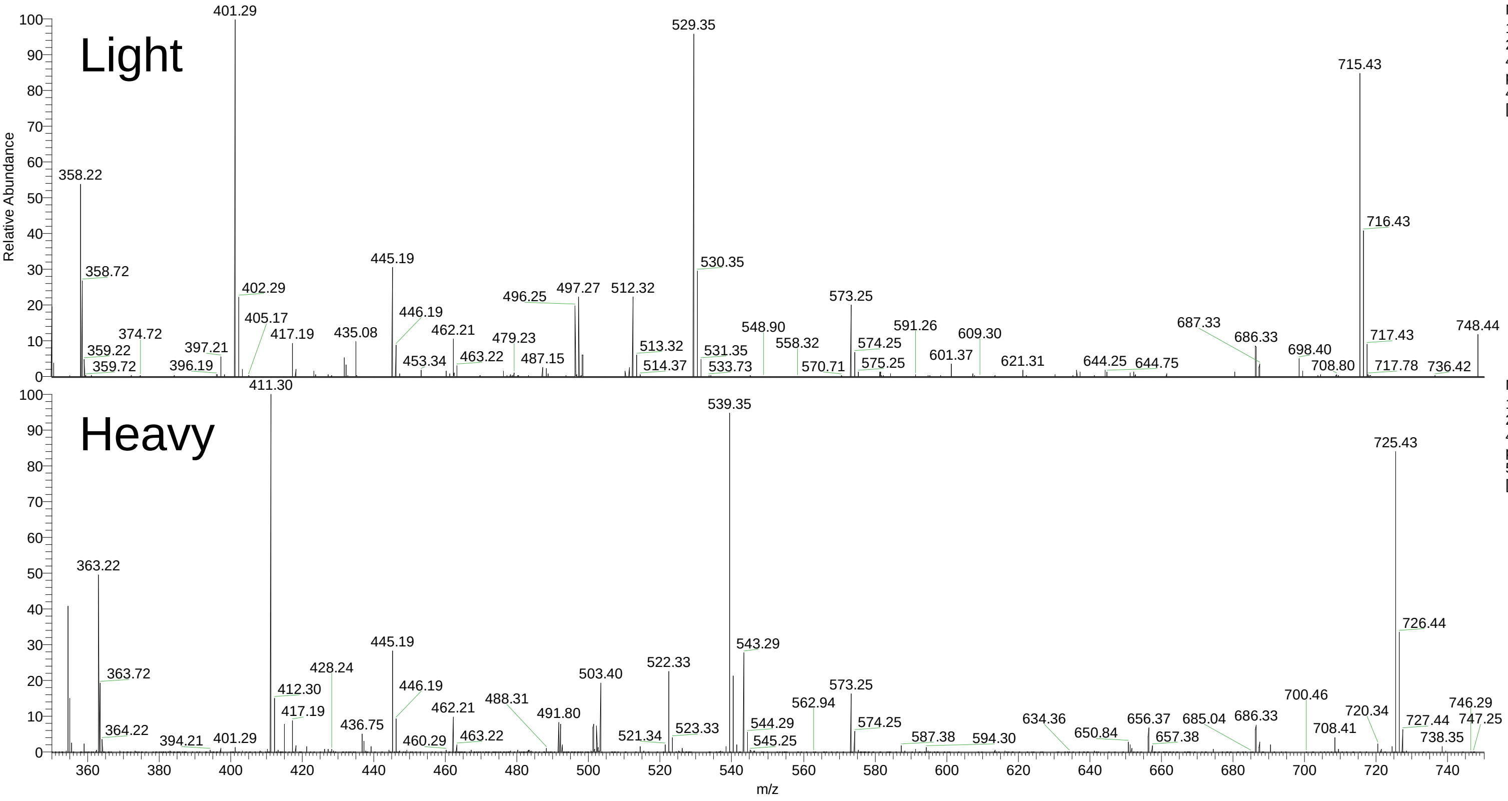

Au; TPP1_61-78; LSELVQAVSDPSSPQYGK

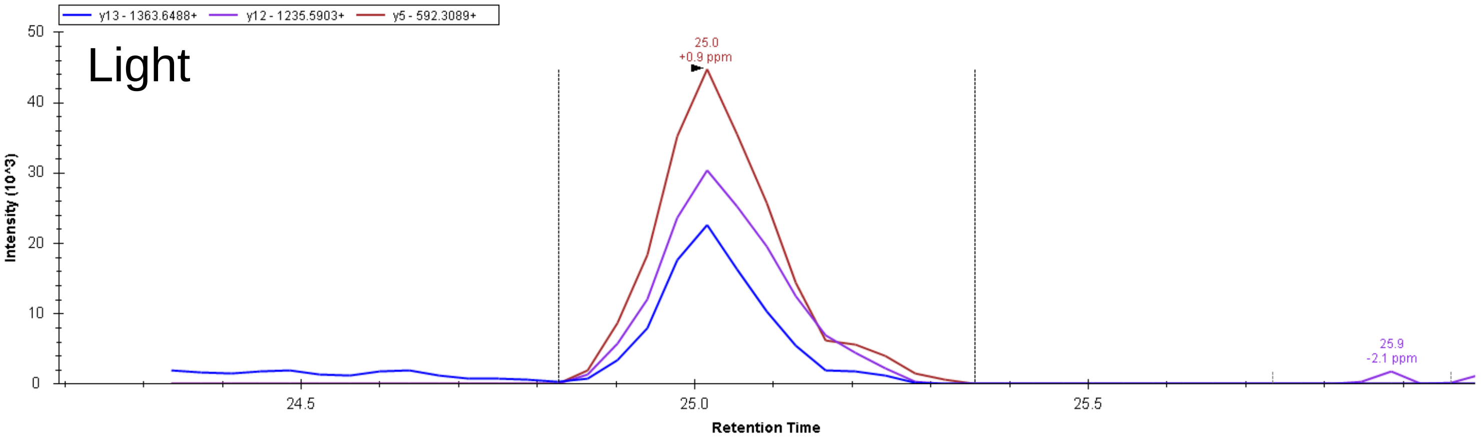

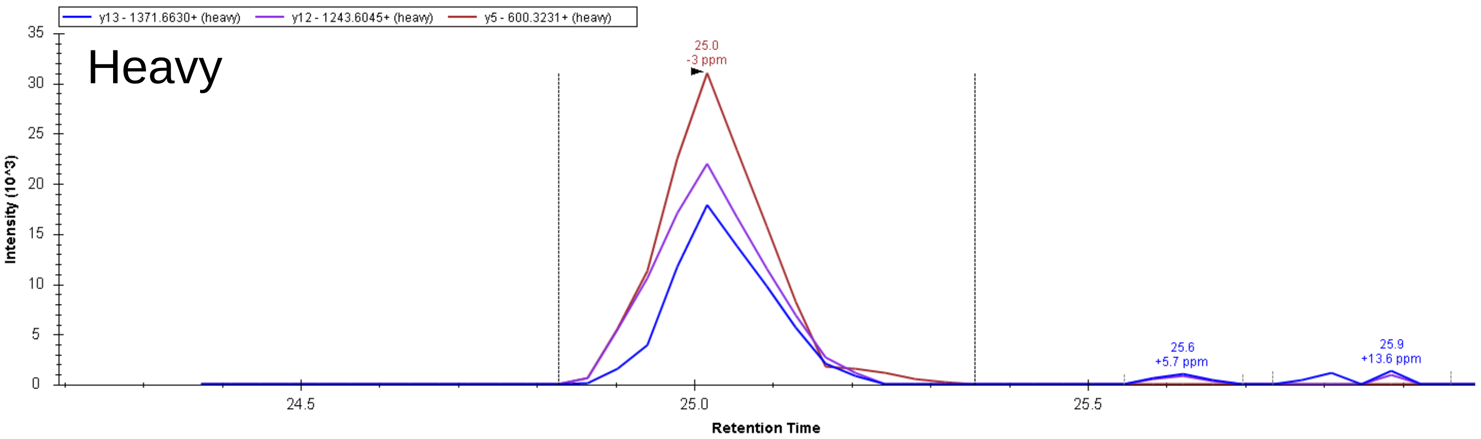

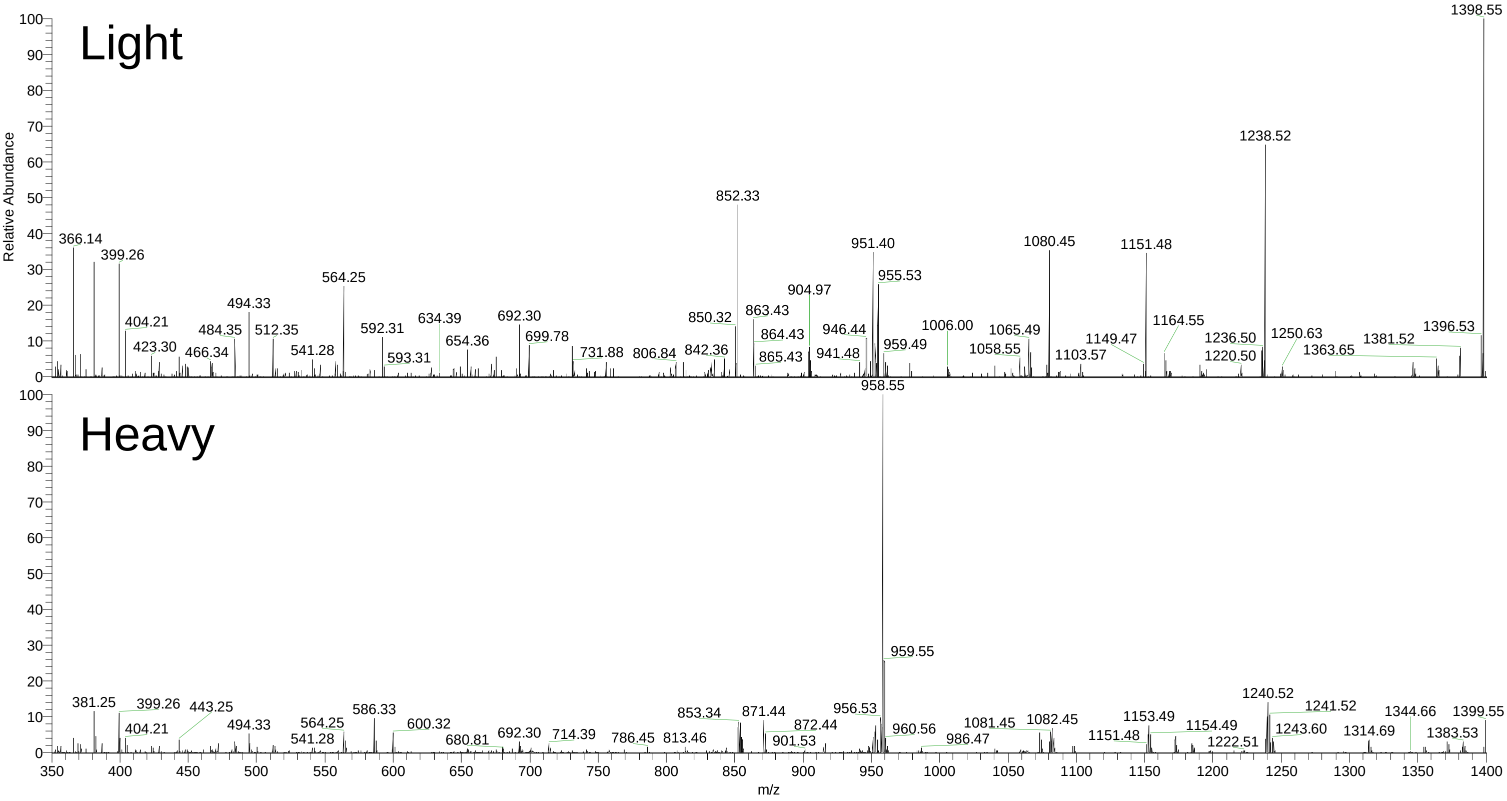

Av; TPP1_246-259; LFGGNFAHQASVAR

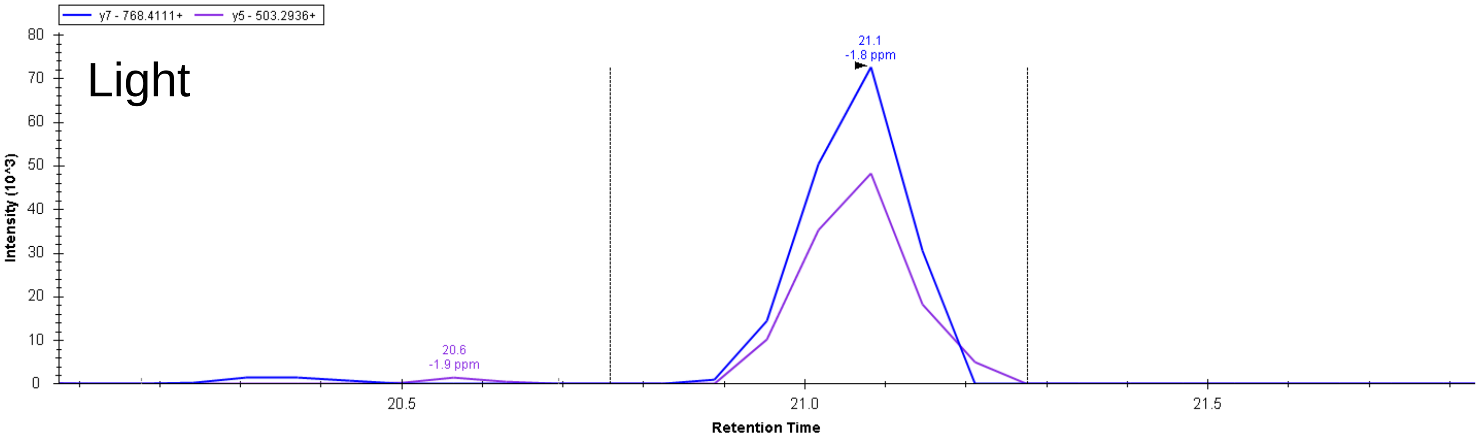

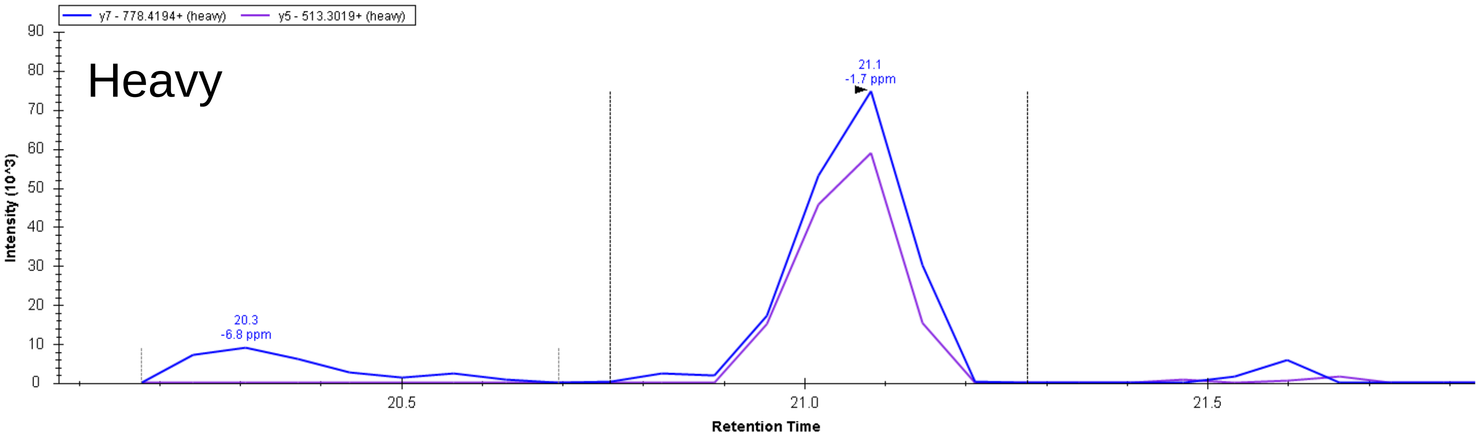

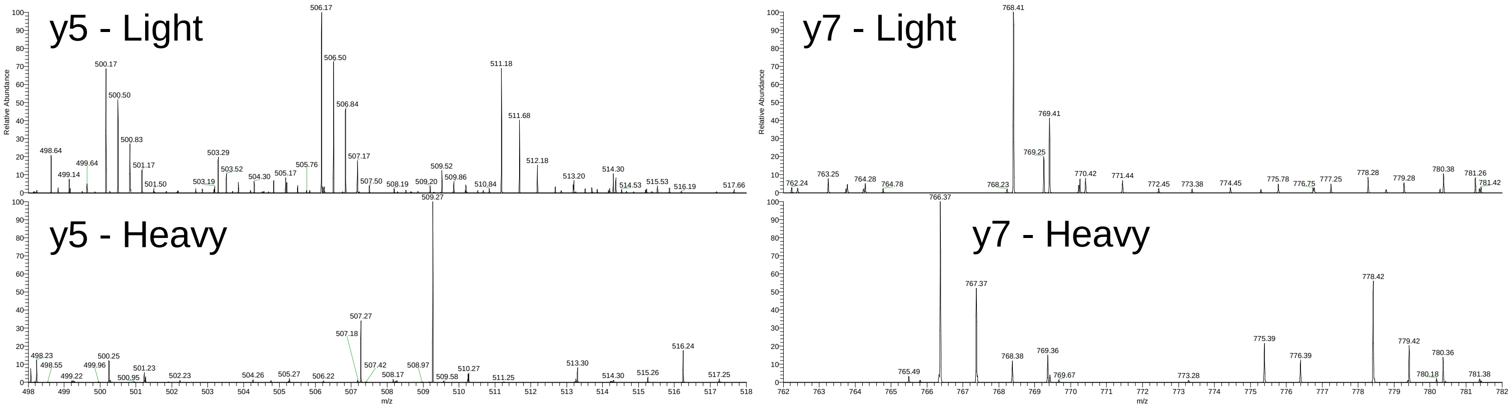

Aw; TPP1_507-520; LYQQHGAGLFDVTR

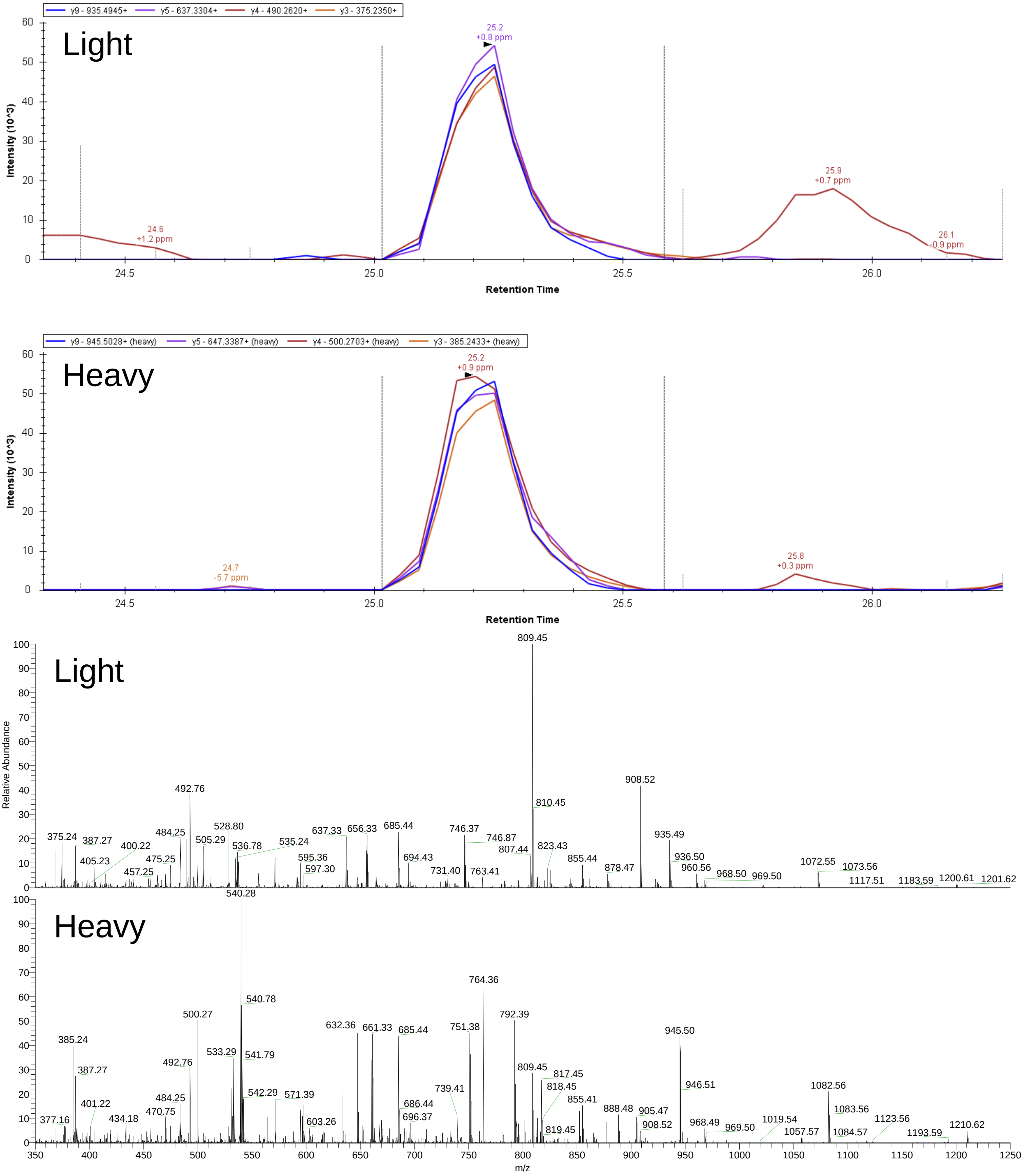

Ax; Ubiquitin_12-27; TITLEVEPSDTIENVK

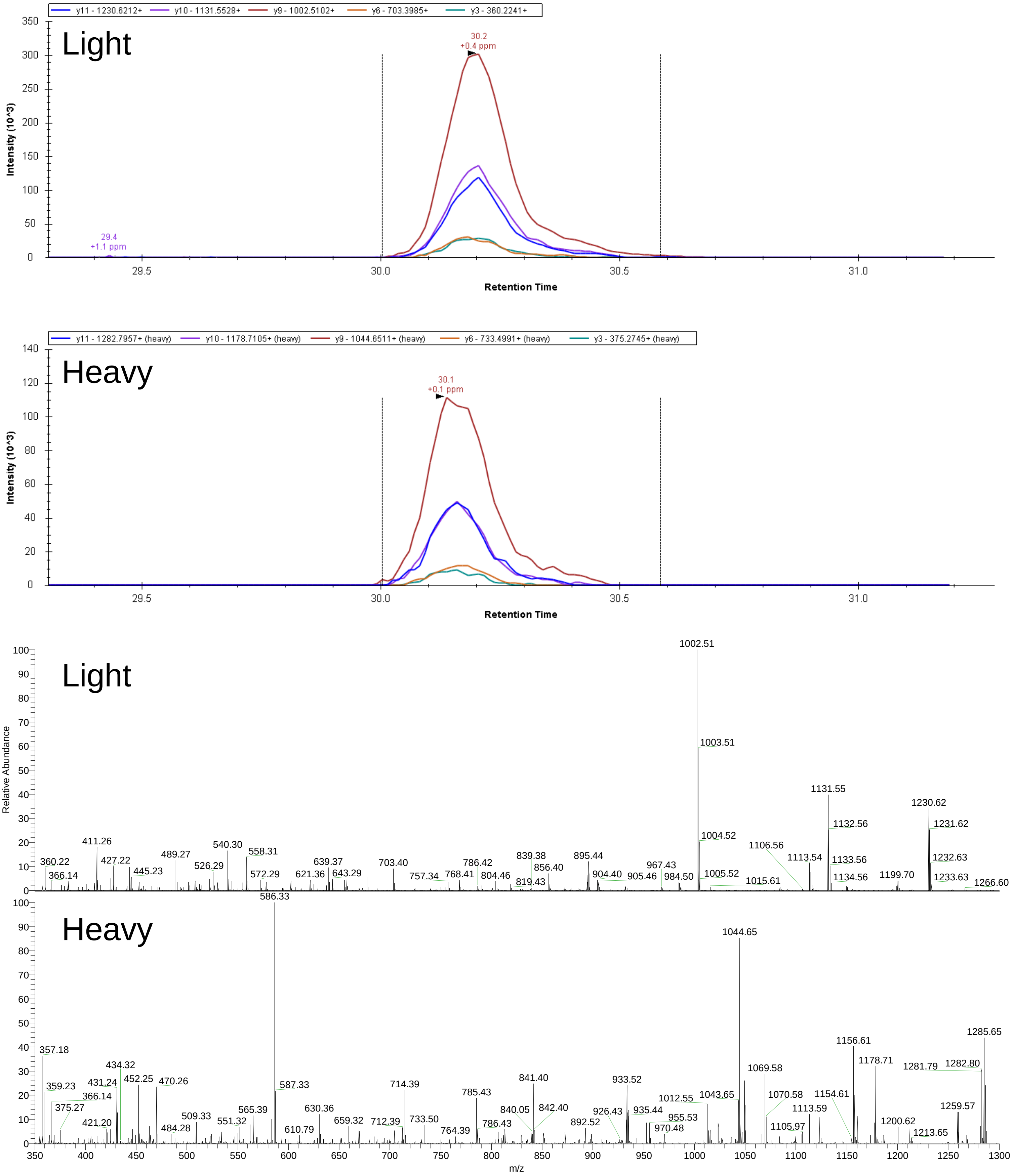

Ay; Ubiquitin_64-72; ESTLHLVLR

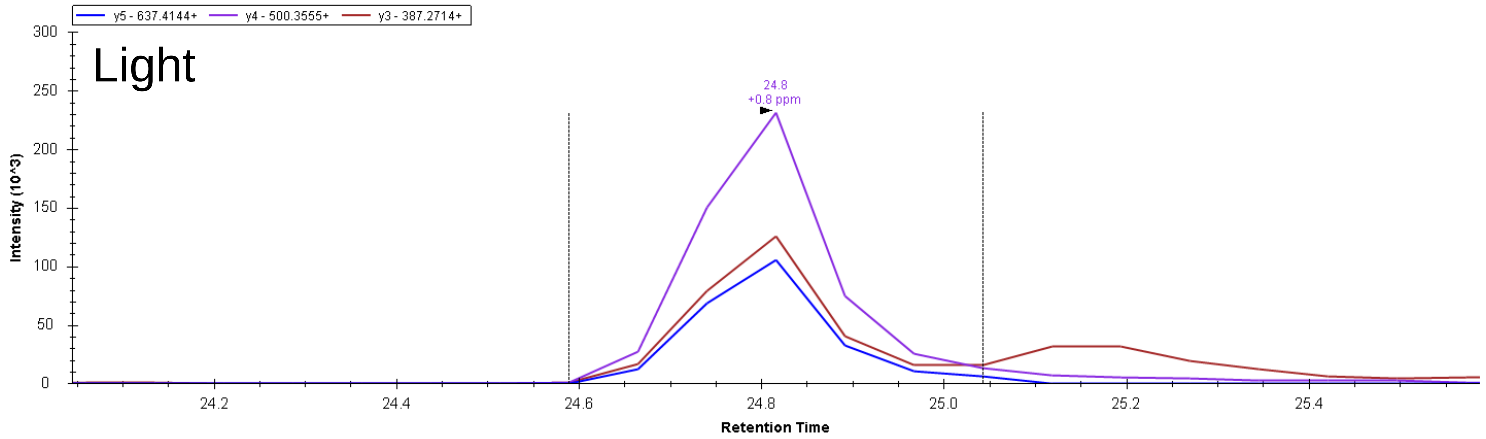

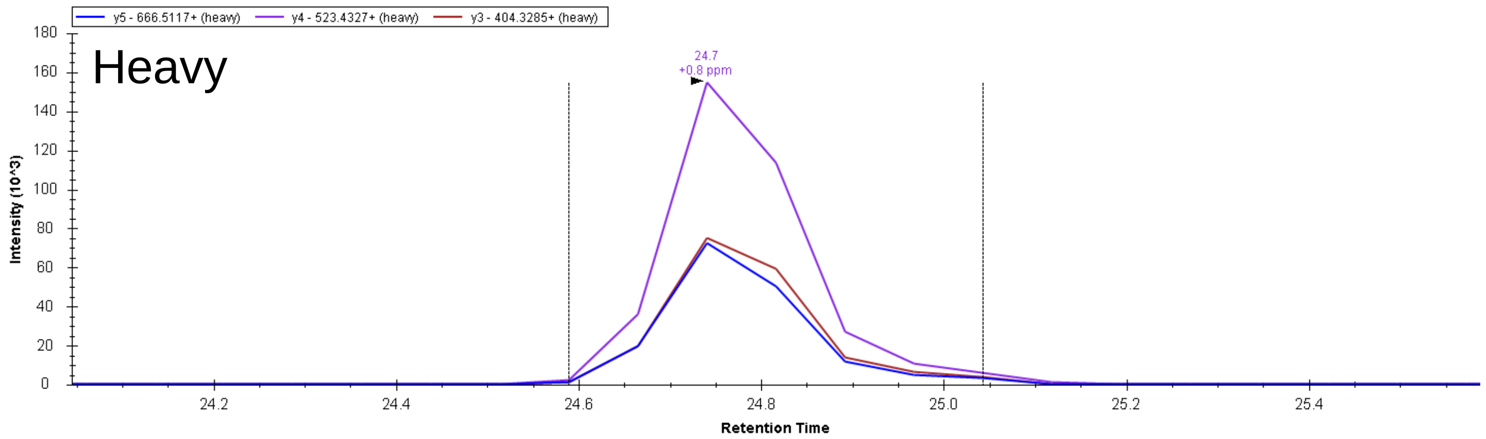

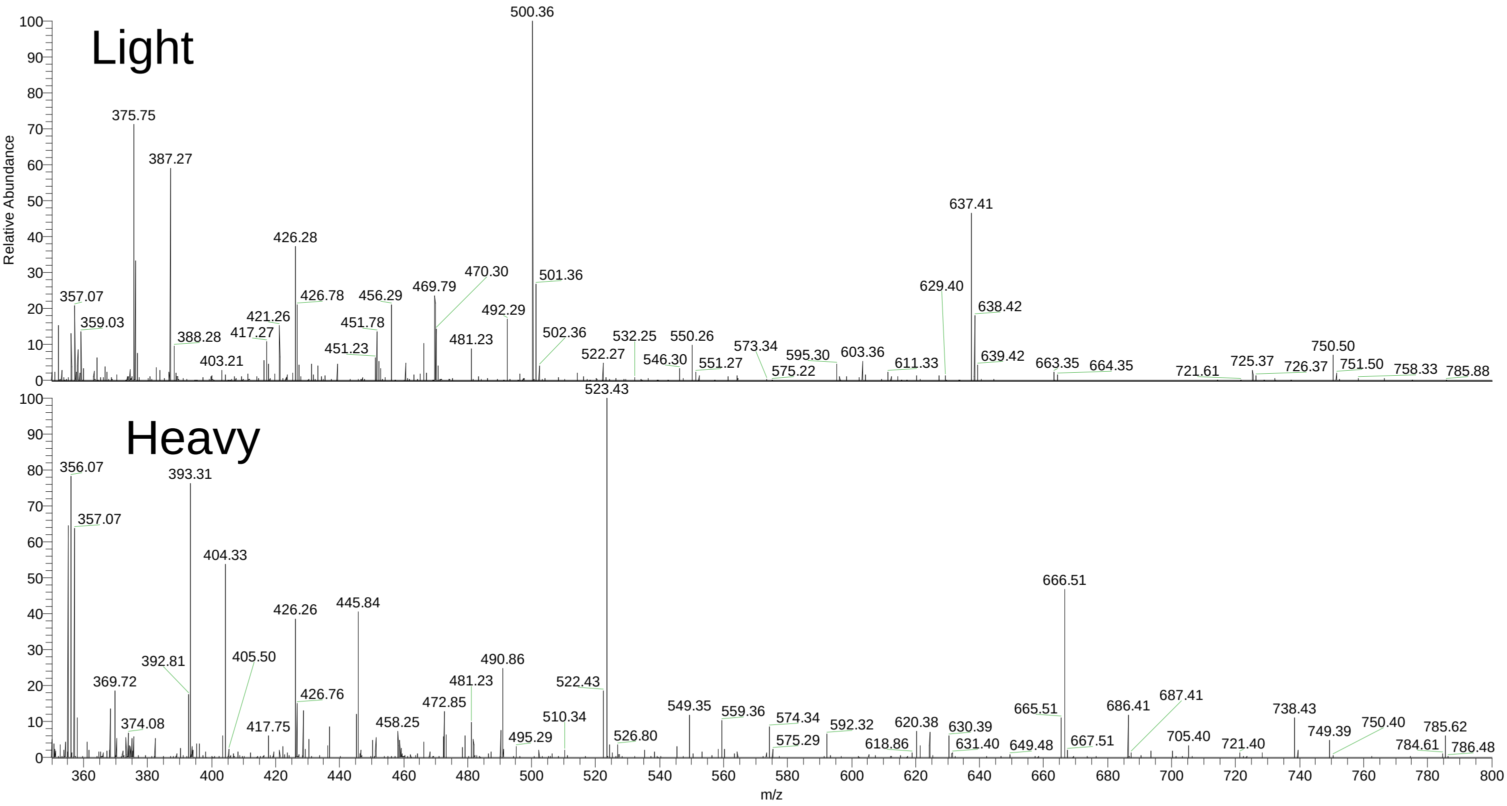

Supplement: Supplementary file 5 — Figure S2. Extracted ion chromatograms and MS/MS spectra. (PDF 4086 kb) [file 13195_2019_533_MOESM5_ESM.pdf]
