# Supplementary material for: Endo-lysosomal proteins and ubiquitin CSF concentrations in Alzheimer’s and Parkinson’s disease
Source: Alzheimers Res Ther. 2019 Sep 14;11:82. doi: 10.1186/s13195-019-0533-9 (PMC6745076; doi:10.1186/s13195-019-0533-9)
Supplement: Supplementary file 8 — Figure S3. Significant peptides in clinical studies I and II. (PDF 218 kb) [file 13195_2019_533_MOESM8_ESM.pdf]

Additional File 8

Endo-Lysosomal Proteins and Ubiquitin CSF Concentrations in Alzheimer's and Parkinson's Disease

Simon Sjödin[1,2], Gunnar Brinkmalm[1,2], Annika Öhrfelt[1,2], Lucilla Parnetti[3], Silvia Paciotti[4], Oskar Hansson[5,6], John Hardy[7], Kaj Blennow[1,2], Henrik Zetterberg[1,2,7,8], Ann Brinkmalm[1,2]

[1]Department of Psychiatry and Neurochemistry, Institute of Neuroscience and Physiology, the Sahlgrenska Academy at the University of Gothenburg, Mölndal, Sweden

[2]Clinical Neurochemistry Laboratory, Sahlgrenska University Hospital, Mölndal, Sweden

[3]Neurology Clinic, University of Perugia, Perugia, Italy

[4]Department of Pharmaceutical Sciences, University of Perugia, Perugia, Italy

[5]Clinical Memory Research Unit, Department of Clinical Sciences Malmö, Lund University, Lund, Sweden

[6]Memory Clinic, Skåne University Hospital, Malmö, Sweden

[7]Department of Molecular Neuroscience, University College London Institute of Neurology, Queen Square, London, UK

[8]UK Dementia Research Institute at UCL, London, United Kingdom

Corresponding Author: Simon Sjödin, Department of Psychiatry and Neurochemistry, Institute of Neuroscience and Physiology, the Sahlgrenska Academy at the University of Gothenburg, House V3, SU/Mölndal, SE-43180, Mölndal, Sweden. simon.sjodin@neuro.gu.se.

Content

Figure S3

Figure S3. Significant peptides in Clinical Studies I and II. Peptides identified with a significantly different CSF concentration in either Clinical Study I or Clinical Study II, or both, are shown; (A) AP2B1_712-719, (B) AP2B1_835-842, (C) AP2B1_868-878 (the peptide was not measured in Clinical Study II), (D) C9_232-242, (E) CTSB_80-87, (F) CTSF_103-116, (G) CTSF_236-245, (H) CTSF_266-278, (I) CTSF_442-450, (J) GM2A_89-96, (K) GM2A_170-179, (L) LAMP1_357-363, (M) LAMP2_133-144, (N) LAMP2_153-161, (O) LAMP2_281-289, (P) TCN2_45-59, (Q) TCN2_300-313, (R) Ubiquitin_12-27, and (S) Ubiquitin_64-72. Clinical Study I (filled symbols) included cross-sectional samples from clinically characterized subjects with AD (N = 6) and PD (N = 10) as well as longitudinal samples from subjects with prodromal AD (N = 10) and stable MCI (N = 15). Clinical Study II (open symbols) included cross-sectional samples from clinically characterized subjects with AD (N = 36) and PD (N = 11) as well as cognitively normal healthy controls (N = 44). Statistics were calculated using Kruskal-Wallis test with Dunn's test for multiple comparisons comparing groups within the studies, not between, and the graphs show Tukey boxplots.

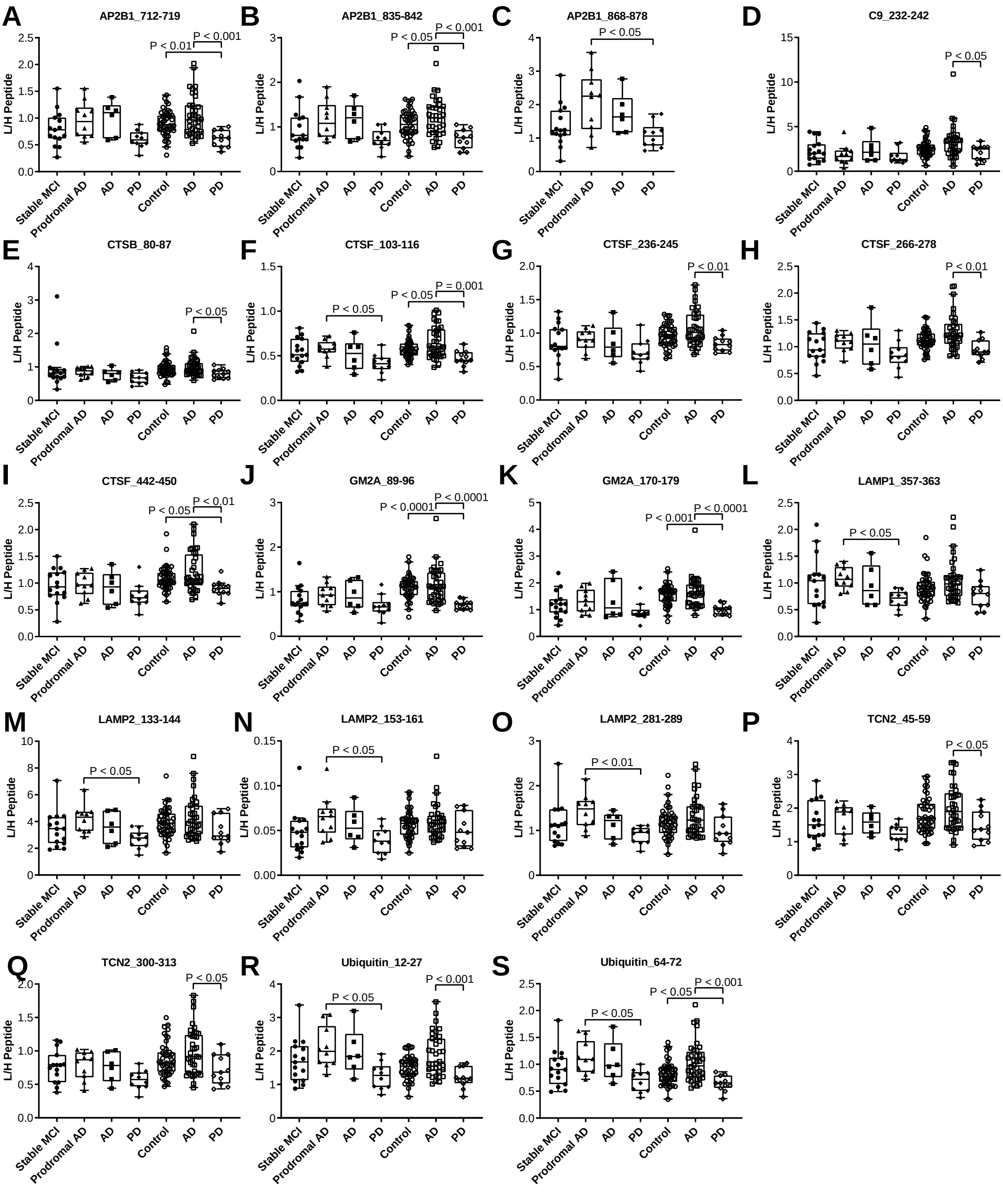

A
AP2B1_712-719
P < 0.01
P < 0.001
B
AP2B1_835-842
P < 0.05
P < 0.001
C
AP2B1_868-878
P < 0.05
D
C9_232-242
P < 0.05
E
CTSB_80-87
P < 0.05
F
CTSF_103-116
P < 0.05
P < 0.05
P = 0.001
G
CTSF_236-245
P < 0.01
H
CTSF_266-278
P < 0.01
I
CTSF_442-450
P < 0.05
P < 0.01
J
GM2A_89-96
P < 0.0001
P < 0.0001
K
GM2A_170-179
P < 0.001
P < 0.0001
L
LAMP1_357-363
P < 0.05
M
LAMP2_133-144
P < 0.05
N
LAMP2_153-161
P < 0.05
O
LAMP2_281-289
P < 0.01
P
TCN2_45-59
P < 0.05
Q
TCN2_300-313
P < 0.05
R
Ubiquitin_12-27
P < 0.05
P < 0.001
S
Ubiquitin_64-72
P < 0.05
P < 0.05
P < 0.001
L/H Peptide
Stable MCI
Prodromal AD
AD
PD
Control
AD
PD
