# Supplementary material for: Endo-lysosomal proteins and ubiquitin CSF concentrations in Alzheimer’s and Parkinson’s disease
Source: Alzheimers Res Ther. 2019 Sep 14;11:82. doi: 10.1186/s13195-019-0533-9 (PMC6745076; doi:10.1186/s13195-019-0533-9)
Supplement: Supplementary file 9 — Figure S4. CSF protein concentrations in APOE ε4 carriers. (PDF 193 kb) [file 13195_2019_533_MOESM9_ESM.pdf]

Additional File 9

Endo-Lysosomal Proteins and Ubiquitin CSF Concentrations in Alzheimer's and Parkinson's Disease

Simon Sjödin[1,2], Gunnar Brinkmalm[1,2], Annika Öhrfelt[1,2], Lucilla Parnetti[3], Silvia Paciotti[4], Oskar Hansson[5,6], John Hardy[7], Kaj Blennow[1,2], Henrik Zetterberg[1,2,7,8], Ann Brinkmalm[1,2]

[1]Department of Psychiatry and Neurochemistry, Institute of Neuroscience and Physiology, the Sahlgrenska Academy at the University of Gothenburg, Mölndal, Sweden
[2]Clinical Neurochemistry Laboratory, Sahlgrenska University Hospital, Mölndal, Sweden
[3]Neurology Clinic, University of Perugia, Perugia, Italy
[4]Department of Pharmaceutical Sciences, University of Perugia, Perugia, Italy
[5]Clinical Memory Research Unit, Department of Clinical Sciences Malmö, Lund University, Lund, Sweden
[6]Memory Clinic, Skåne University Hospital, Malmö, Sweden
[7]Department of Molecular Neuroscience, University College London Institute of Neurology, Queen Square, London, UK
[8]UK Dementia Research Institute at UCL, London, United Kingdom
Corresponding Author: Simon Sjödin, Department of Psychiatry and Neurochemistry, Institute of Neuroscience and Physiology, the Sahlgrenska Academy at the University of Gothenburg, House V3, SU/Mölndal, SE-43180, Mölndal, Sweden. simon.sjodin@neuro.gu.se.

Content

Figure S4

Figure S4. CSF protein concentrations in *APOE* ε4 carriers. In Clinical Study II a subset of the participants had known *APOE* genotype. *APOE* ε4 carriers (*APOE* ε4 +), having one or two ε4 alleles, were compared to non-carriers (*APOE* ε4 –). The carriers vs. non-carriers where compared within the groups, controls (carriers, N = 10, non-carriers, N = 17) and AD (carriers, N = 25, non-carriers, N =10), respectively. Significant different CSF concentrations were identified between control carriers and control non-carriers for peptides (A) C9_232-242 ($P < 0.05$), (B) C9_497-508 ($P < 0.05$), (C) CTSF_442-450 ($P < 0.05$), (D) DPP7_113-123 ($P < 0.05$) and (E) GM2A_89-96 ($P < 0.05$). Statistics were calculated using Wilcoxon 2-sample rank sum test and the graphs show Tukey boxplots.

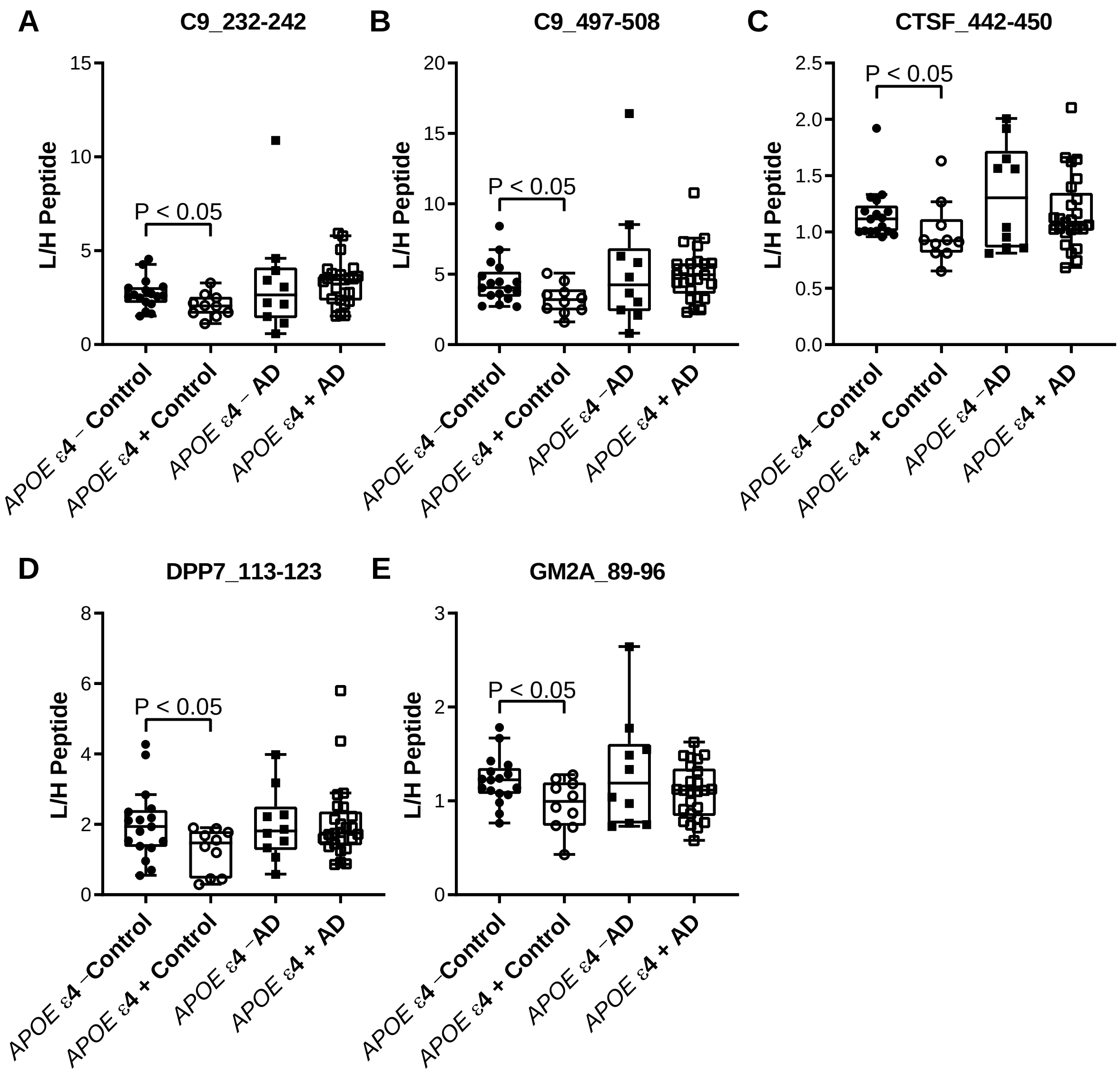
A
C9_232-242
B
C9_497-508
C
CTSF_442-450
D
DPP7_113-123
E
GM2A_89-96
L/H Peptide
P < 0.05
APOE ε4 − Control
APOE ε4 + Control
APOE ε4 − AD
APOE ε4 + AD
